# Sublingual Edaravone Dexborneol for the Treatment of Acute Ischemic Stroke: The TASTE-SL Randomized Clinical Trial

**DOI:** 10.1001/jamaneurol.2023.5716

**Published:** 2024-02-19

**Authors:** Yu Fu, Anxin Wang, Renhong Tang, Shuya Li, Xue Tian, Xue Xia, Jinsheng Ren, Shibao Yang, Rong Chen, Shunwei Zhu, Xiaofei Feng, Jinliang Yao, Yan Wei, Xueshuang Dong, Yun Ling, Fei Yi, Qian Deng, Cunju Guo, Yi Sui, Shugen Han, Guoqiang Wen, Chuanling Li, Aiqin Dong, Xin Sun, Zhimin Wang, Xueying Shi, Bo Liu, Dongsheng Fan

**Affiliations:** 1 Department of Neurology, Peking University Third Hospital, Beijing, China; 2 Beijing Neurosurgical Institute, Capital Medical University, Beijing, China; 3 State Key Laboratory of Neurology and Oncology Drug Development, Nanjing, China; 4 Department of Neurology, Beijing Tiantan Hospital, Capital Medical University, Beijing, China; 5 China National Clinical Research Center for Neurological Diseases, Beijing Tiantan Hospital, Capital Medical University, Beijing, China; 6 Department of Clinical Epidemiology and Clinical Trial, Capital Medical University, Beijing, China; 7 Beijing Municipal Key Laboratory of Clinical Epidemiology, Beijing, China; 8 Simcere Pharmaceutical Group Limited, Nanjing, China; 9 Neurodawn Pharmaceutical Co Ltd, Nanjing, China; 10 Harrision International Peace Hospital, Hengshui, China; 11 Daqing Oilfied General Hospital, Daqing, China; 12 Nanshi Hospital of Nanyang, Nanyang, China; 13 Pingxiang People’s Hospital, Pingxiang, China; 14 The First Affiliated Hospital of Nanyang Medical College, Nanyang, China; 15 Liaocheng People’s Hospital, Liaocheng, China; 16 The First People’s Hospital of Shenyang, Shenyang, China; 17 Mei He Kou Central Hospital, Jilin, China; 18 Hainan General Hospital, Hainan, China; 19 Xuzhou Central Hospital, Jiangsu, China; 20 Cangzhou Central Hospital, Hebei, China; 21 The First Hospital of Jilin University, Jilin, China; 22 Taizhou First People’s Hospital, Zhejiang, China; 23 Anqing Municipal Hospital, Anhui, China; 24 The First Affiliated Hospital Baotou Medical College, Baotou, China

## Abstract

**Importance:**

Sublingual edaravone dexborneol, which can rapidly diffuse and be absorbed through the oral mucosa after sublingual exposure, is a multitarget brain cytoprotection composed of antioxidant and anti-inflammatory ingredients edaravone and dexborneol.

**Objective:**

To investigate the efficacy and safety of sublingual edaravone dexborneol on 90-day functional outcome in patients with acute ischemic stroke (AIS).

**Design, Setting, and Participants:**

This was a double-blind, placebo-controlled, multicenter, parallel-group, phase 3 randomized clinical trial conducted from June 28, 2021, to August 10, 2022, with 90-day follow-up. Participants were recruited from 33 centers in China. Patients randomly assigned to treatment groups were aged 18 to 80 years and had a National Institutes of Health Stroke Scale score between 6 and 20, a total motor deficit score of the upper and lower limbs of 2 or greater, a clinically diagnosed AIS symptom within 48 hours, and a modified Rankin Scale (mRS) score of 1 or less before stroke. Patients who did not meet the eligibility criteria or declined to participate were excluded.

**Intervention:**

Patients were assigned, in a 1:1 ratio, to receive sublingual edaravone dexborneol (edaravone, 30 mg; dexborneol, 6 mg) or placebo (edaravone, 0 mg; dexborneol, 60 μg) twice daily for 14 days and were followed up until 90 days.

**Main Outcomes and Measures:**

The primary efficacy outcome was the proportion of patients with mRS score of 1 or less on day 90 after randomization.

**Results:**

Of 956 patients, 42 were excluded. A total of 914 patients (median [IQR] age, 64.0 [56.0-70.0] years; 608 male [66.5%]) were randomly allocated to the edaravone dexborneol group (450 [49.2%]) or placebo group (464 [50.8%]). The edaravone dexborneol group showed a significantly higher proportion of patients experiencing good functional outcomes on day 90 after randomization compared with the placebo group (290 [64.4%] vs 254 [54.7%]; risk difference, 9.70%; 95% CI, 3.37%-16.03%; odds ratio, 1.50; 95% CI, 1.15-1.95, *P* = .003). The rate of adverse events was similar between the 2 groups (89.8% [405 of 450] vs 90.1% [418 of 464]).

**Conclusion and Relevance:**

Among patients with AIS within 48 hours, sublingual edaravone dexborneol could improve the proportion of those achieving a favorable functional outcome at 90 days compared with placebo.

**Trial Registration:**

ClinicalTrials.gov Identifier: NCT04950920

## Key Points

**Question:**

Does sublingual edaravone dexborneol improve functional outcome in patients with acute ischemic stroke?

**Findings:**

In this randomized clinical trial including 914 patients who received sublingual edaravone dexborneol or placebo, the proportion of patients achieving a favorable outcome defined as a 90-day modified Rankin Scale score of 1 or less was 64.4% in the sublingual edaravone dexborneol group, which was significantly higher than 54.7% in the placebo group. The rate of adverse events was similar between the 2 groups.

**Meaning:**

Sublingual edaravone dexborneol, as a fast-acting and convenient agent, could improve the proportion of patients who achieve good clinical outcomes at 90 days compared with placebo among patients with acute ischemic stroke.

## Teaser

This randomized clinical trial investigates if sublingual edaravone dexborneol improves functional outcome in patients with acute ischemic stroke.

## Introduction

The major goal of intervention in acute ischemic stroke (AIS) is to salvage the ischemic penumbra.^1^ Brain cytoprotection, as proposed by Stroke Treatment Academic Industry Roundtable (STAIR), has the capability of reducing ischemic brain injury by antagonizing detrimental molecular events in all brain components.^2,3^ Given the intended goal for broad protection, cytoprotective approaches that exert pleiotropic effects on multiple targets of the ischemic cascade, including excitotoxicity, oxidative stress, inflammation, apoptosis, etc, are the priority recommendation.^2^ Recently, the Efficacy and Safety of Nerinetide for the Treatment of Acute Ischemic Stroke (ESCAPE-NA1) trial reported that eicosapeptide nerinetide had a potentially cytoprotective effect via inhibition of neuronal excitotoxicity and reduction of the production of nitric oxide among patients with AIS who were not treated with alteplase after endovascular thrombectomy. The findings suggested that brain cytoprotection in human stroke might be possible and promising, although further confirmations are still required.^4^

Edaravone dexborneol is another intravenously administered multitarget brain cytoprotective agent composed of edaravone and dexborneol.^5^ The 2 components of edaravone dexborneol were shown to be effective in the prevention and treatment of AIS.^6,7^ The Treatment of Acute Ischemic Stroke with Edaravone Dexborneol (TASTE) trial has indicated that, compared with edaravone alone, intravenous edaravone dexborneol could improve 90-day functional outcomes in patients with AIS.^8,9^ However, intravenous drug administration largely depends on health care resources, which may be delayed or limited during busy periods (ie, times during which there are many hospital admissions) and thereby cause a significant barrier in terms of time delay to protect neuronal function. Sublingual edaravone dexborneol, an innovative, multitarget drug composed of edaravone and dexborneol, can rapidly diffuse and be absorbed through the oral mucosa after sublingual exposure, without interfering with disposition and elimination properties and significantly alter the total bioavailability of edaravone and dexborneol. Whether it could provide a rapid brain cytoprotective effect for patients with AIS remains unclear. Therefore, the Treatment of Acute Ischemic Stroke with Sublingual Edaravone Dexborneol (TASTE-SL) trial was designed to investigate the effects of sublingual edaravone dexborneol on 90-day functional outcome in patients with AIS.

## Methods

### Study Design and Patients

This was a phase 3, double-blind, placebo-controlled, multicenter, parallel-group, randomized clinical trial conducted in 33 centers in China between June 28, 2021, and August 10, 2022. The ethics committee from each study center approved this study, and all patients or their legally acceptable surrogates had given informed consent before they were assigned to a treatment group. The study was registered with ClinicalTrials.gov. Written informed consent for participation in the trial was provided by the patients or their legal representative. Details of the trial rationale, design, and methods have been described previously and are provided in the protocol (Supplement 1).^10^ Patients were eligible if they were aged from 18 to 80 years old, had a National Institutes of Health Stroke Scale (NIHSS) score between 6 and 20, had a total motor deficit score of the upper and lower limbs of 2 or greater, had a clinically diagnosed AIS symptom within 48 hours, and had a modified Rankin Scale (mRS) score of 1 or less before the stroke. All patients included in the study were of Chinese Han ethnicity. Coordinating centers and clinical site information are available in eAppendix 1 and 2 in Supplement 2. Detailed exclusion criteria are shown in eTable 1 in Supplement 2. The study was conducted in accordance with the principles of the Declaration of Helsinki and followed the Consolidated Standards of Reporting Trials (CONSORT) reporting guidelines.

### Randomization and Masking

Within 48 hours after symptom onset, eligible patients were randomly assigned in a 1:1 ratio to receive sublingual edaravone dexborneol or placebo. The randomization was stratified by clinical centers and time onset of AIS (≤24 hours and >24 hours). Randomization was implemented via a centralized, interactive web-based response system.

The packaged, labeled, and assigned progressions were conducted by an independent clinical research organization. The 2 drugs were identical in color and shape. All drugs were concealed in uniform packages with the same label, which did not indicate the contents of the package, and 1 single-use drug tablet was packed in a small box, with each large box containing 30 small boxes (including 2 small boxes of backup drugs). Drug management personnel distributed the study drugs according to the requirements of the clinical trial protocol and recorded in a timely fashion the distribution and recovery of each study drug on a special record sheet. The blinding and allocation concealment were guaranteed.

### Treatment

Patients in the sublingual edaravone dexborneol group received a sublingual dose of edaravone dexborneol, 36 mg (edaravone, 30 mg; dexborneol, 6 mg), twice a day for 14 consecutive days. Patients in the placebo group received a sublingual placebo drug (edaravone, 0 mg; dexborneol, 60 μg [simulated the cool taste of sublingual edaravone dexborneol]) twice a day for 14 consecutive days. All patients were followed up to day 90 after randomization.

### Outcomes

The primary efficacy outcome was the proportion of patients with an mRS score of 1 or less on day 90 after randomization. The secondary outcomes included mRS score on day 90, the proportion of patients with an mRS score of 2 or less on day 90, the change in NIHSS score from baseline to day 14, and the proportion of patients with an NIHSS score of 1 or less on day 14, 30, and 90 after randomization. Safety outcomes included adverse events, treatment-related adverse events within 90 days, and changes in vital signs and laboratory data before and after treatment.

### Statistical Analysis

Assuming that the proportion of patients with an mRS score of 1 or less on day 90 was 50% in the sublingual edaravone dexborneol group and 40% in the placebo group, a 2-sided α of .05, power of 80%, and dropout rate of 15% were used to estimate the total sample size. One prespecified interim analysis for sample size re-estimation by an independent data monitoring committee was conducted according to conditional power calculations as indicated by the promising zone method when 50% of patients were enrolled, and no need for an adaptive increase in sample size was found.

All the analyses were performed in the intention-to-treat population. Baseline characteristics were presented as median with IQR for continuous variables and frequency with proportion for categorical variables. Missing data on the primary outcome were handled with treatment policy strategy, composite variable strategies, and while on treatment strategies.^11^ Group difference in the primary efficacy outcome was calculated, and the corresponding 95% CIs of the difference between proportions were estimated based on normal approximation. Odds ratios (ORs) with 95% CIs were calculated using logistic regression.

Similar approaches were used for binary secondary outcomes, including mRS score of 2 or less on day 90; NIHSS score of 1 or less on day 14, 30, and 90; and safety outcomes on adverse events and treatment-related adverse events. For mRS score on day 90, an ordinal logistic regression analysis was performed, with the results presented as common OR and 95% CI, where a common OR in favor of sublingual edaravone dexborneol was greater than 1.0. For changes in NIHSS score from baseline to day 14, mean values with 95% CIs were calculated for each group, and mean differences with 95% CI between the groups were estimated by analysis of covariance. In addition, a post hoc sensitivity analysis was performed using different approaches to impute missing data on the primary efficacy outcome and complete case analysis. Finally, the heterogeneity of treatment effects on the primary outcome among several prespecified subgroups was evaluated by including the interaction term between treatment and subgroup effect into the logistic regression model.

All tests were 2-sided, and *P* < .05 was considered statistically significant. Statistical analyses were performed with SAS software, version 9.4 (SAS Institute).

## Results

### Baseline Characteristics

**Figure 1.**
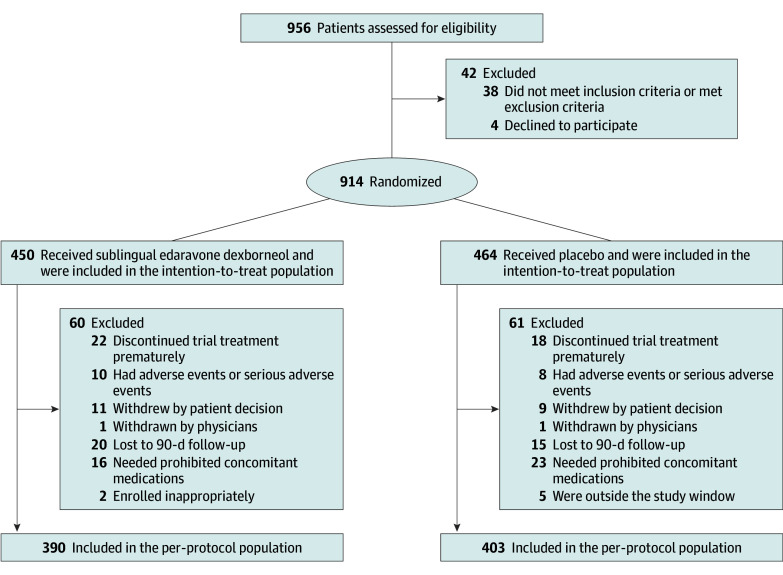
Enrollment and Randomization of the Patients The prohibited concomitant medications included drugs with indications of neuroprotection or cerebral infarction and unmarketed drugs or other drugs for clinical trials.

Among 956 patients screened, 914 patients (median [IQR] age, 64.0 [56.0-70.0] years; 608 male [66.5%]; 306 female [33.5%]) were randomized, among whom 450 patients (49.2%) received sublingual edaravone dexborneol, and 464 patients (50.8%) received placebo (Figure 1). Baseline characteristics of the patients were similar in the 2 groups (Table 1). A total of 838 patients (91.7%) had an mRS score of 0. Concomitant treatment taken during the treatment period is reported in eTable 2 in Supplement 2. Among the treated patients, 793 patients (390 [49.2%] in the edaravone dexborneol group and 403 [50.8%] in the placebo group) did not have major violations of the study protocol (eg, inappropriate enrollment, premature discontinuation of trial treatment, concomitant use of prohibited medications [drugs that may interact with the trial drug as described in the protocol], or were outside of the study window or lost to 90-day follow-up) and therefore were included in the per-protocol analysis (Figure 1).

**Table 1.** Baseline Characteristics

Characteristics	Edaravone dexborneol group (n = 450)	Placebo group (n = 464)
Age, median (IQR), y	64.1 (56.0-69.8)	64.6 (57.1-71.4)
Sex, No. (%)		
Female	141 (31.3)	165 (35.6)
Male	309 (68.7)	299 (64.4)
BMI, median (IQR)^a^^,^^b^	24.6 (22.5-26.6)	24.7 (22.5-26.8)
Blood pressure, median (IQR), mm Hg		
Systolic	147 (135-162)	147 (136-160)
Diastolic	86 (79-95)	86 (80-94)
Medical history, No. (%)		
Stroke	124 (27.6)	143 (30.8)
Hypertension	367 (81.6)	366 (78.9)
Diabetes	184 (40.9)	187 (40.3)
Dyslipidemia	217 (48.2)	221 (47.6)
Coronary heart disease	132 (29.3)	136 (29.3)
NIHSS score, median (IQR)	7 (7-8)	7 (7-8)
mRS score before onset, No. (%)		
0	415 (92.2)	423 (91.2)
1	35 (7.8)	41 (8.8)
Time to randomization, h		
≤24	196 (43.6)	203 (43.8)
>24	254 (56.4)	261 (56.3)
TOAST subtype, No. (%)^a^		
Large-artery atherosclerosis	247 (55.8)	245 (53.5)
Cardioembolism	10 (2.3)	16 (3.5)
Small-vessel occlusion	178 (40.2)	185 (40.4)
Other determined etiology	7 (1.6)	11 (2.4)
Undetermined etiology	1 (0.2)	1 (0.2)
Kidney function^c^		
Normal	399 (88.7)	414 (89.2)
Mildly impaired	47 (10.4)	48 (10.3)
Moderately to severely impaired	4 (0.9)	2 (0.4)

Abbreviations: BMI, body mass index; mRS, modified Rankin Scale; NIHSS, National Institutes of Health Stroke Scale; TOAST, Trial of Org 10172 in Acute Stroke Treatment.

^a^ The number of patients with missing data on BMI was 20 in the edaravone dexborneol group and 17 in the placebo group; missing data on TOAST subtypes was 7 in the edaravone dexborneol group and 6 in the placebo group.

^b^ Calculated as weight in kilograms divided by height in meters squared.

^c^ Kidney function was defined according to estimated glomerular filtration rate (eGFR, calculated as milliliters per minute per 1.73 m^2^) as follows: normal kidney function (baseline eGFR ≥90), mild kidney impairment (60 ≤ baseline eGFR <90), and moderate to severe kidney impairment (baseline eGFR <60).

### Efficacy Outcomes

In the primary analysis population, missing data on mRS was considered to be 6 for patients without at least 1 postbaseline measurement of mRS (8 patients in the edaravone dexborneol group and 8 in the placebo group) or discontinued treatment or follow-up due to adverse events (8 and 14), and the last observation carried forward approach was used for patients who had at least 1 valid postbaseline measurement (24 and 10). A favorable outcome of an mRS score of 1 or less on day 90 occurred in 290 of 450 patients (64.4%) in the edaravone dexborneol group and in 254 of 464 patients (54.7%) in the placebo group (risk difference, 9.70%; 95% CI, 3.37%-16.03%; OR, 1.50; 95% CI, 1.15-1.95, *P* = .003) (Table 2). Sensitivity analysis with different approaches to imputation on missing primary data (eTable 3 in Supplement 2) and the per-protocol analysis (eTable 4 in Supplement 2) showed similar results.

**Table 2.** Efficacy and Safety Outcomes

Outcomes	Edaravone dexborneol group (n = 450)	Placebo group (n = 464)	Effect size (95% CI)	*P* value
Primary outcome				
mRS score ≤1 on day 90, No. (%)^a^^,^^b^	290 (64.4)	254 (54.7)	RD 9.70 (3.37 to 16.03) OR 1.50 (1.15 to 1.95)	.003
Secondary outcomes				
mRS score on day 90, median (IQR)	1 (0-2)	1 (1-3)	Common OR 1.33 (1.05 to 1.68)	.02
mRS score ≤2 on day 90, No. (%)	342 (76.0)	337 (72.6)	RD 3.37 (−2.29 to 9.03) OR 1.19 (0.89 to 1.61)	.24
Changes of NIHSS score from baseline to day 14, mean (95% CI)^a^	−3 (−4 to −3)	−3 (−4 to −3)	MD −0.11 (−0.49 to 0.27)	.58
NIHSS score ≤1 on day 14, No. (%)^a^	63 (15.0)	71 (16.2)	RD −1.25 (−6.10 to 3.60) OR 0.91 (0.63 to 1.32)	.62
NIHSS score ≤1 on day 30, No. (%)^a^	146 (35.9)	136 (32.2)	RD 3.64 (−2.80 to 10.09) OR 1.18 (0.88 to 1.57)	.27
NIHSS score ≤1 on day 90, No. (%)^a^	230 (57.2)	215 (51.6)	RD 5.66 (−1.16 to 12.47) OR 1.26 (0.95 to 1.65)	.11
Safety outcomes				
AE within 90 d, No. (%)	405 (89.8)	418 (90.1)	RD −0.31 (−4.21 to 3.59) OR 0.97 (0.63 to 1.49)	.88
TRAE within 90 d, No. (%)	61 (13.6)	50 (10.8)	RD 2.78 (−1.46 to 7.02) OR 1.30 (0.87 to 1.93)	.20
SAE within 90 d, No. (%)	42 (9.3)	47 (10.1)	−0.80 (−4.64 to 3.05) 0.91 (0.59 to 1.42)	.69

Abbreviations: AE, adverse event; MD, mean difference; mRS, modified Rankin Scale; NIHSS, National Institutes of Health Stroke Scale; OR, odds ratio; RD, risk difference; SAE, severe adverse event; TRAE, treatment-related adverse event.

^a^ Missing data on mRS was considered to be 6 for patients without at least 1 postbaseline measurement of mRS (8 patients in the edaravone dexborneol group and 8 in the placebo group) or discontinued treatment or follow-up due to adverse events (8 and 14), and the last observation carried forward approach was used for patients who had at least 1 valid postbaseline measurement (24 and 10). The number of patients with missing data on NIHSS score on day 14 was 29 in the edaravone dexborneol group and 26 in the placebo group; missing data on NIHSS score on day 30 was 43 in the edaravone dexborneol group and 42 in the placebo group; missing data on NIHSS score on day 90 was 48 in the edaravone dexborneol group and 47 in the placebo group.

^b^ A total of 20 patients had telephone assessment of the mRS, and 64 had video assessment of the mRS.

**Figure 2.**
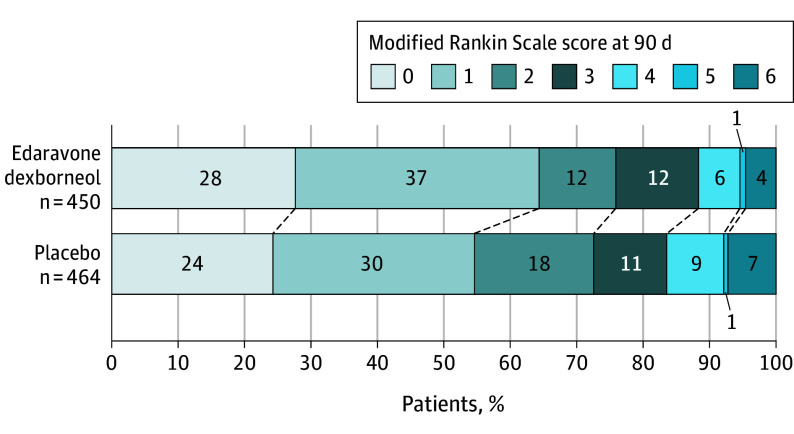
Distribution of Functional Outcomes at 90 Days in the Intention-to-Treat Population Shown are scores on the modified Rankin Scale (mRS) for the patients in the 2 treatment groups. Scores range from 0 to 6, with 0 indicating no symptoms; 1, no clinically significant disability; 2, slight disability (patients are able to look after their own affairs without assistance but are unable to carry out all previous activities); 3, moderate disability (patients require some help but are able to walk unassisted); 4, moderately severe disability (patients are unable to attend to bodily needs without assistance and are unable to walk unassisted); 5, severe disability (patients require constant nursing care and attention); and 6, death. Missing data on mRS was considered to be 6 for patients without at least 1 postbaseline measurement of mRS (8 patients in the edaravone dexborneol group, and 8 in the placebo group), or discontinued treatment or follow-up due to adverse events (8 and 14), and the last observation carried forward approach was used for patients who had at least 1 valid postbaseline measurement (24 and 10).

**Figure 3.**
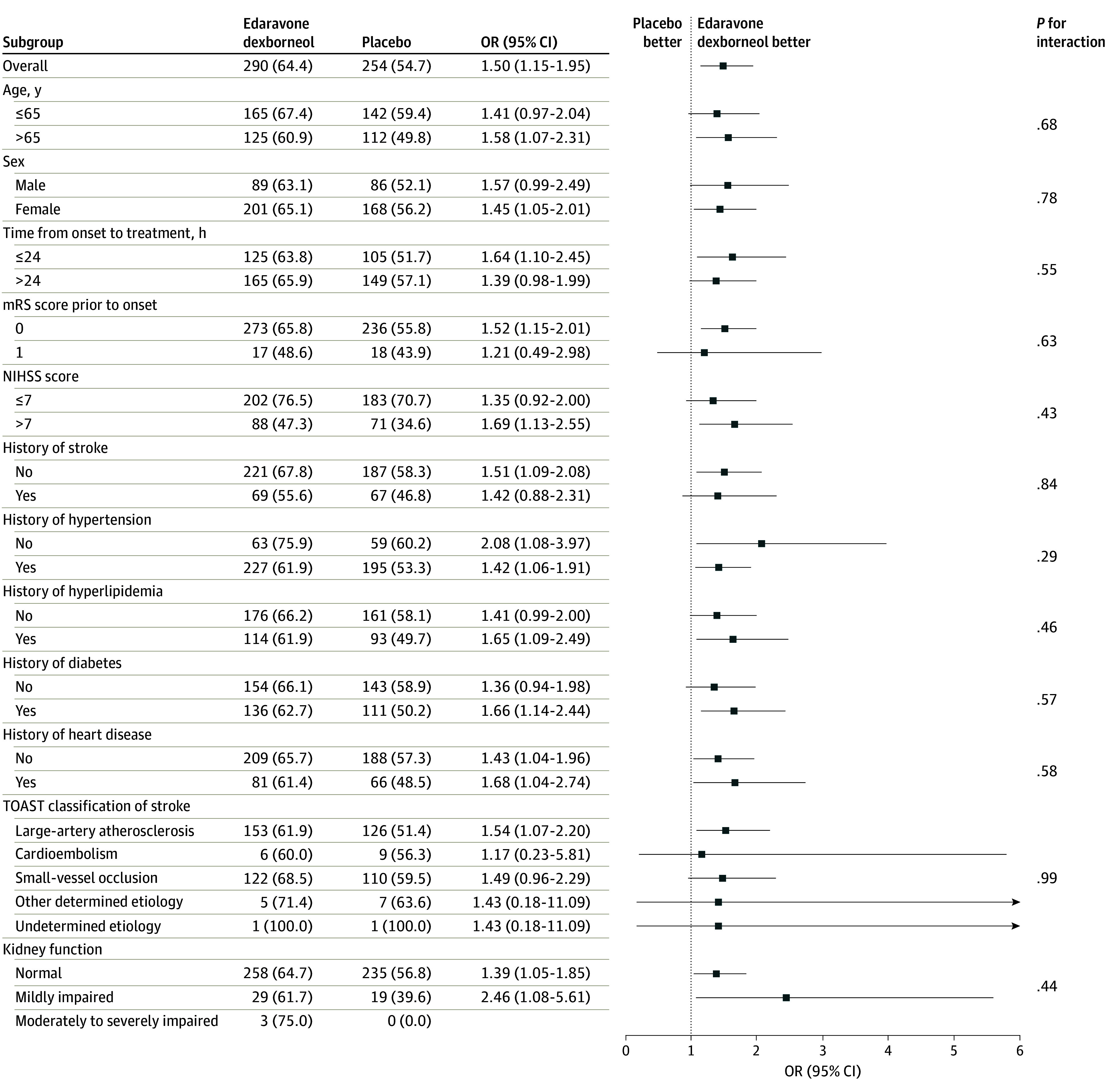
Subgroup Analysis for the Primary Analysis mRS indicates modified Rankin Scale; NIHSS, National Institutes of Health Stroke Scale; OR, odds ratio; TOAST, Trial of Org 10172 in Acute Stroke Treatment.

For the secondary outcomes, an ordinal comparison of the distribution of patients across mRS categories showed that the good function outcome favored the edaravone dexborneol group (common OR, 1.33; 95% CI, 1.05-1.68; *P* = .02) (Table 2 and Figure 2). However, edaravone dexborneol had no effect on other prespecified secondary outcomes, including the proportion of patients with an mRS score of 2 or less, the difference between the groups in the change of NIHSS score from baseline to day 14, and the proportion of NIHSS score of 1 or less on day 14, 30, and 90 (Table 2 and eTable 3 in Supplement 2). The results of the subgroup analyses for the primary outcome suggest that the effect of sublingual edaravone dexborneol was consistent across multiple subgroups (Figure 3).

### Safety Outcomes

Adverse events occurred in 405 patients (89.8%) in the edaravone dexborneol group and in 418 patients (90.1%) in the placebo group (risk difference, −0.31%; 95% CI, −4.21% to 3.59%; OR, 0.99; 95% CI, 0.64-1.53) (Table 2). Treatment-related adverse events occurred in 61 patients (13.6%) in the edaravone dexborneol group and in 50 patients (10.8%) in the placebo group (risk difference, 2.78%; 95% CI, −1.46% to 7.02%; OR, 1.30; 95% CI, 0.87-1.93) (Table 2). Detailed information on adverse events is presented in eTables 5 to 12 in Supplement 2.

## Discussion

In this double-blind, placebo-controlled, randomized clinical trial involving patients with AIS within 48 hours after symptom onset, sublingual edaravone dexborneol could improve the proportion of patients achieving a good functional outcome on day 90 after randomization without increasing risks of any adverse events.

Despite compelling evidence for the potential benefit of brain cytoprotection observed in the preclinical animal models, most of the previously published clinical trials targeting brain cytoprotection failed to show significant clinical efficacy for patients with AIS during the last decade. The Stroke-Acute Ischemic NXY Treatment (SAINT) I and II trials, for instance, suggested that NXY-059, a free radical–trapping agent, was ineffective for the treatment of AIS within 6 hours of symptom onset.^12^ Similar nonsignificant efficacy was also found for uric acid in the Safety and Efficacy of Uric Acid in Patients with Acute Stroke (URICO-ICTUS) trials,^13^ for magnesium sulfate in the Field Administration of Stroke Therapy-Magnesium (FAST-MAG) trial,^14^ for intravenous albumin in the Albumin in Acute Ischemic Stroke (ALIAS) trials,^15^ and for natalizumab in the Safety and Efficacy of Natalizumab in Patients with Acute Ischemic Stroke (ACTION) trial,^16^ respectively. However, the ESCAPE-NA1^4^ and TASTE trials reported a better 90-day good functional outcome for patients treated with brain cytoprotective agents.^8^ These trials indicated that although no significant clinical benefits were found for most brain cytoprotective agents developed based on the single-pathway strategy, treatments targeting several pathways of ischemic injury seemed to be more promising due to the complicated mechanism of injury in ischemic stroke.

Sublingual edaravone dexborneol is a brain cytoprotective agent composed of edaravone and dexborneol, 2 active ingredients with synergistic antioxidant and anti-inflammatory effects. Edaravone is an effective oxygen radical scavenger that has long been recommended for AIS therapy in China and Japan.^17,18,19^ Laboratory and clinical evidence have indicated that edaravone could lessen neuronal damage and endothelial cell injury through inhibition of neurotoxicity, reduction of chronic inflammation, and regulation of the expression of endothelial and neuronal protein in the ischemic cascade progress.^6,20,21,22,23,24,25^ Dexborneol, the other ingredient of sublingual edaravone dexborneol, serves as an important component of proprietary Chinese medicine for the treatment of stroke. It was proved to prevent neuronal injury after cerebral ischemia via multiple action mechanisms, including improvement of cerebral blood flow, inhibition of neuronal excitotoxicity, resistance to reactive oxygen species injury, and inhibition of inflammatory processes and caspase-related apoptosis. On the other hand, the high permeability of dexborneol could promote other agents to pass through the blood-brain barrier to exert synergistic therapeutic effects.^26^ Accordingly, the preclinical trial has shown that edaravone dexborneol was more effective in protecting the brain from ischemic and/or ischemic reperfusion injury, thereby avoiding the potential deficiency of those single-target agents.^5^ Through these mechanisms, our study showed that sublingual edaravone dexborneol improved the favorable outcome, defined as an mRS score of 0 to 1 after 90 days of randomization, for patients with AIS.

Sublingual agents can quickly disintegrate once in contact with the saliva and before being swallowed, which leads to a high drug concentration in situ and rapid absorption through the highly permeable sublingual mucosa.^27,28^ Pharmacokinetic studies have shown that sublingual formulation rapidly diffused and was absorbed across the buccal membrane without interfering with disposition and elimination properties and without significantly altering the total bioavailability of edaravone and dexborneol. Consequently, the time to peak plasma concentration and the area under the curve remain largely unchanged. Sublingual edaravone dexborneol is the first, to our knowledge, sublingual brain cytoprotective agent administered to patients with AIS and provides several clinical advantages compared with intravenous drugs, including faster onset of action, lower dose requirement, better patient compliance and convenience, and increased bioavailability. As acknowledged, time to treatment is a criteria factor in the odds of achieving good outcomes after stroke; acute-stage therapies should be administered immediately once a stroke has been identified. Sublingual edaravone dexborneol, as a rapidly acting, patient-friendly brain cytoprotection, showed good efficacy on functional outcomes in patients with AIS in this trial. Taken together, sublingual edaravone dexborneol could be administrated earlier to patients with AIS in future clinical practice regardless of health care resources, even for those who are in a coma, experience dysphagia, are at home, or are en route to the hospital in the ambulance.

### Limitations

There were several limitations in our study. First, we did not compare the effect of sublingual edaravone dexborneol and intravenous edaravone dexborneol. However, the total bioavailability of sublingual edaravone dexborneol and the edaravone dexborneol injection was similar according to pharmacokinetic data. In addition, considering the same components of the drugs and the blinding settings, sublingual placebo as the control group may be a better choice. Second, patients who received endovascular therapy were excluded from this study. In recent years, given the rapid development progress in the field of endovascular therapy, the effect of sublingual edaravone dexborneol in patients receiving endovascular therapy should be investigated in future investigations. Third, our study enrolled a substantial number of patients with a relatively low NIHSS score (mild stroke), which resulted in nonsignificant results for the secondary outcomes. Fourth, some information was not available in our trial, such as results of the EuroQol Health Questionnaire and hemorrhagic event data; therefore, further investigations are warranted to investigate the effect of sublingual edaravone dexborneol on these outcomes. Finally, this trial was conducted among Chinese patients, thus the findings should be validated in other ethnic groups.

## Conclusions

In this randomized clinical trial involving patients with AIS treated within 48 hours after symptom onset, sublingual edaravone dexborneol provided brain cytoprotection by improving functional outcomes after 90 days of randomization.

## Notes

Supplement 1.
Trial Protocol.

Supplement 2. **eAppendix 1.** Committees and Coordinating Centers in the TASTE-SL Trial **eAppendix 2.** Clinical Sites in Order of Participants Enrolled **eTable 1.** Inclusion and Exclusion Criteria **eTable 2.** Concomitant Medication Use Within 90 Days **eTable 3.** Sensitivity Analyses for the Primary Analysis **eTable 4.** Efficacy and Safety Outcomes in the Per-Population Analysis **eTable 5.** Number of Patients With Adverse Events (by System Organ Class ) During 90 Days of Follow-Up **eTable 6.** Number of Patients With Severe Adverse Events (by System Organ Class ) During 90 Days of Follow-Up **eTable 7.** Number of Patients With Treatment Related Adverse Events During 90 Days of Follow-Up **eTable 8.** Number of Patients With Clinically Significant Changes in Vital Signs and Laboratory Data on Day 90 of Follow-Up **eTable 9.** Laboratory Data on Day 90 After Randomization **eTable 10.** Laboratory Data on Day 30 After Randomization **eTable 11.** Laboratory Data on Day 14 After Randomization **eTable 12.** Laboratory Data on Day 7 After Randomization

Supplement 3.
Data Sharing Statement.
