# Supplementary material for: Sublingual Edaravone Dexborneol for the Treatment of Acute Ischemic Stroke: The TASTE-SL Randomized Clinical Trial
Source: JAMA Neurol. 2024 Feb 19;81(4):319–26. doi: 10.1001/jamaneurol.2023.5716 (PMC10877503; doi:10.1001/jamaneurol.2023.5716)
Supplement: Supplement 1. — Trial Protocol. [file jamaneurol-e235716-s001.pdf]

Phase III Clinical Trial of Treatment of Acute Ischemic STroke with Sublingual Edaravone Dexborneol (TASTE-SL)

Protocol

Research team: Peking University Third Hospital

Principal investigator: Dongsheng Fan

Protocol version **3.0**

Date **June 20, 2022**

Catalog

Catalog 2
Abstract of research protocol 5
Abrreviations 11
1. Introduction 13
  1.1. Background 13
  1.2. Background of the investigational product 15
    1.2.1. Overview 15
    1.2.2. Mechanism of action 16
    1.2.3. Preclinical study 16
      1.2.3.1. Preclinical pharmacodynamics of Y-2 Sublingual Tablets 16
      1.2.3.2. Preclinical toxicology of Y-2 18
      1.2.3.3. Toxicology 19
      1.2.3.4. Non-clinical pharmacokinetics of Y-2 Sublingual Tablets 26
      1.2.3.5. Clinical pharmacokinetics 30
  1.3. Risk 34
2. Objective and Outcomes 36
  2.1. Objective 36
  2.2. Outcomes 36
    2.2.1. Primary efficacy outcomes 36
    2.2.2. Secondary efficacy outcomes 37
    2.2.3. Safety outcomes 37
  2.3. Main estimated target 37
3. Study design 38
  3.1. General design 38
  3.2. Sample size 39
  3.3. Scientific basis for trial design 40
  3.4. Hospitalization time 41
  3.5. Definition of end of study 41
4. Population 42
  4.1. Inclusion criteria 42
  4.2. Exclusion criteria 42
  4.3. Definition of onset time 43
5. Study treatment 44
  5.1. Study drug 44
  5.2. Management of study drug 44
    5.2.1. Packaging and labeling of study drug 44
    5.2.2. Receipt and storage of study drug 46
    5.2.3. Distribution, recovery, and destruction of study drug 47
  5.3. Randomization and blinding 47
  5.4. Unblinding 48
    5.4.1. Common unblinding regulations 48
    5.4.2. Emergency unblinding 48

5.5. Treatment protocol ... 49
5.6. Treatment adjustment ... 49
5.7. Criteria for discontinuation of treatment or withdrawal ... 50
5.7.1. Criteria for discontinuation of or withdrawal from the treatment ... 50
5.7.2. Handling of discontinuation of or withdrawal from the treatment ... 51
5.7.3. Criteria for early discontinuation ... 51
5.8. Previous/concomitant medication and therapy ... 52
5.8.1. Allowed concomitant medication/therapy ... 52
5.8.2. Prohibited concomitant medication/therapy ... 53
6. Study process ... 55
6.1. Screening period (visit 1) ... 58
6.2. Treatment period ... 58
6.2.1. D7 (±1) (visit 2) ... 58
6.2.2. D14 (+3) (visit 3) ... 59
6.3. Follow-up period ... 59
6.3.1. D30 (±7) (visit 4) ... 59
6.3.2. D90 (±7) (visit 5) ... 59
6.3.3. Follow-up endpoint ... 60
7. Study measures ... 60
7.1. Efficacy measures ... 60
7.2. Safety measures ... 60
7.2.1. Safety evaluation indexes ... 60
7.2.2. Laboratory test and evaluation on safety ... 61
7.3. Safety report and evaluation ... 62
7.3.1. Definition of adverse event, serious adverse event and suspected unexpected serious adverse event ... 62
7.3.2. Time period and frequency of collecting information related to adverse events and serious adverse events ... 64
7.3.3. Methods for collecting adverse events and serious adverse events ... 65
7.3.4. Follow-up visit of adverse events and serious adverse events ... 65
7.3.5. Record and evaluation of adverse events and serious adverse events ... 66
7.3.6. Requirements for regulatory reporting of serious adverse events ... 68
7.3.7. Serious adverse event reporting to the Sponsor ... 69
7.3.8. Pregnancy ... 69
8. Statistical analysis ... 70
8.1. Determination of sample size ... 70
8.2. Analysis sets ... 70
8.3. Baseline analysis ... 71
8.4. Efficacy analysis ... 71
8.5. Safety analysis ... 72
8.6. Interim analysis ... 73
9. Supporting Documents and Relevant Considerations for Clinical Practice ... 74
9.1. Data management ... 74

9.1.1. Data acquisition 74
9.1.2. Data recording 75
9.1.3. Database locking 76
9.1.4. Data archiving 76
9.2. Ethics 76
9.2.1. Responsibilities of the investigator 76
9.2.2. Ethical requirements 77
9.2.3. Ethical norms for the study 77
9.2.4. Informed consent of subjects 78
9.2.5. Confidentiality of subject information 79
9.3. Quality assurance and quality control 79
9.3.1. Quality control of the study implemented by the Sponsor 79
9.3.2. Quality control of the study implemented by the investigator 80
9.3.3. Quality control monitoring 81
9.3.4. Data quality control 82
9.4. Data preservation and confidentiality 83
9.5. Protocol revision 83
9.6. Protocol deviation 84
9.7. Publication of study results 84
10. References 84
11. Appendices 85
Appendix 1 Key Points in Diagnosis of Cerebrovascular Diseases (Excerpt) 85
Appendix 2 Modified Rankin Scale 89
Appendix 3 NIH Stroke Scale (NIHSS) 91
Appendix 4 SSS TOAST Classification 101

Abstract of research protocol

**Title**	Phase III Clinical Trial of Treatment of Acute Ischemic Stroke with Sublingual Edaravone Dexborneol (TASTE-SL)
**Research Team**	Peking University Third Hospital
**Research Center**	About 40 centers in China
**Indication**	Acute ischemic stroke
**Study Objective**	To evaluate the efficacy and safety of sublingual edaravone dexborneol (also name Y-2 Sublingual Tablets as below) in the treatment of acute ischemic stroke.
**Study Design**	A randomized, double-blind, parallel, placebo-controlled, multicenter clinical trial.
**Sample Size**	A total sample size of 914 patients (457 patients in each group) with an interim analysis performed after approximately 50% of patients complete the visit on Day 90.
**Patients**	**Inclusion criteria**<br>Those who meet all the following requirements:<br>• Age ≥ 18 years old and ≤ 80 years old, regardless of gender;<br>• After the onset of the disease, the National Institutes of Stroke Scale score: 6 ≤ NIHSS ≤ 20, and the sum of the fifth upper limb score and the sixth lower limb score was ≥2;<br>• The onset time is within 48 hours (including 48 hours);<br>• Patients diagnosed as ischemic stroke according to "key points for diagnosis of all kinds of major cerebrovascular diseases in China 2019", with good prognosis after the first attack or the last attack (MRS score ≤ 1 before this attack);<br>• The informed consent approved by the ethics committee was voluntarily signed by the patient or his legal representative.

	**Exclusion criteria**<br><br>Those who meet any of the following items:<br>• Intracranial hemorrhagic diseases seen in head imaging: hemorrhagic stroke, epidural hematoma, intracranial hematoma, intraventricular hemorrhage, subarachnoid hemorrhage, etc; If it is only oozing blood, the researcher can judge whether it is suitable for inclusion;<br>• Severe disturbance of consciousness: the item score of La consciousness level of NIHSS was more than 1;<br>• Transient ischemic attack (TIA);<br>• Systolic blood pressure was still higher than 220mmhg or diastolic blood pressure was higher than 120mmhg after blood pressure control;<br>• Patients with severe mental disorders and dementia;<br>• Severe active liver diseases have been diagnosed, such as acute hepatitis, chronic active hepatitis, cirrhosis, etc; Or ALT or AST > 2.0 × ULN;<br>• Severe active kidney disease and renal insufficiency have been diagnosed; Or serum creatinine > 1.5 × ULN;<br>• After the onset of the disease, the drugs with neuroprotective effect in the manual have been used;<br>• Embolectomy or interventional therapy has been used or planned after the onset of the disease;<br>• Complicated with malignant tumor or undergoing anti-tumor treatment; For the subjects diagnosed with malignant tumor after enrollment, whether to continue to participate in the study can be judged by the researcher and the willingness of the subjects;<br>• Suffering from severe systemic diseases, the estimated survival time is less than 90 days;<br>• Allergic to d-borneol or edaravone or excipients;

	• Patients during pregnancy, lactation and planned pregnancy;<br>• Major operation history within 4 weeks before enrollment;<br>• Have participated in other clinical studies or are participating in other clinical studies within 30 days before randomization;<br>• The researcher thinks that it is not suitable to participate in this clinical study.
**Drugs**	**Intervention drug**<br>• Y-2 sublingual tablets: Edaravone 30mg and dexborneol 6mg.<br>• Manufacturer: Jiangsu Simcere Pharmaceutical Group Limited<br>**Control drug**<br>• Y-2 placebo, 60 μg dexborneol<br>• Manufacturer: Jiangsu Simcere Pharmaceutical Group Limited
**Treatment Protocol**	The study drug was given within 48 hours after symptom onset and patients were randomized to two groups in a 1:1 ratio, stratified by clinical centers and time onset of AIS (≤24 hours and >24 hours).<br>**Intervention Group**<br>• 36 mg dose of Y-2 sublingual tablet (edaravone 30 mg, dexborneol: 6 mg), twice a day for 14 consecutive days;<br>**Placebo Group**<br>• Y-2 sublingual placebo (edaravone 0 mg, dexborneol: 60 μg), twice a day for 14 consecutive days.
**Randomization / Blinding**	To ensure blinding in trials, the allocation of drugs was conducted by professional nurses independently, and blinding was performed by statisticians who were not related to this clinical trial. Y-2 sublingual tablet and placebo were distributed in pre-randomized and the sealed treatment packs are externally indistinguishable.
**Efficacy Measures**	**Primary efficacy measures**<br>• The proportion of patients with mRS score ≤ 1 on Day 90 after randomization.

	**Secondary efficacy measures**<br>• mRS score on day 90.<br>• the proportion of patients achieving a good functional outcome (mRS ≤2) on day 90.<br>• the change in NIHSS score from baseline to 14 days.<br>• the proportion of patients achieving an NIHSS score ≤1 on day 14, 30 and 90 days after randomization.
**Safety measures**	• adverse events within day 90.<br>• treatment related adverse events within day 90.<br>• changes in vital signs and laboratory data before and after treatment.
**Statistical analysis**	**Sample size**<br>The objective of this study is to evaluate the efficacy and safety of Y-2 Sublingual Tablets in the treatment of acute ischemic stroke. The primary efficacy index is the proportion of patients with mRS score ≤ 1 on Day 90 of treatment. The sample size is calculated based on the primary efficacy index. According to the previous clinical studies, it is expected that the proportion of patients with mRS score ≤ 1 on the Day 90 of treatment is 50% in the test group and 40% in the control group. Taking bilateral α as 0.05, the power as 80% and the random ratio as 1:1, the sample size for each group is calculated to be 388. Considering the 15% dropout rate, then it is planned to enroll 457 cases in each group, with a total sample size of 914 cases.<br>An interim analysis is scheduled to be performed after approximately 50% of patients complete the visit on Day 90. The purpose of interim analysis is to reestimate the sample size based on primary efficacy indexes. It is conducted by the Independent Data Monitoring Committee (IDMC) and whether the sample size should be increased or remain unchanged is recommended according to the regulations of the IDMC. The sample size may be increased to 1.5 times the scheduled sample size

	at most, and won't be reduced. If the sample size is adjusted, the method proposed by Cui et al. 1999 (hereinafter referred to as the CHW method) will be used to control type I errors in the final analysis.<br>**Analysis sets**<br>• **Full Analysis Set (FAS):** According to the basic principles of intention-to-treatment (ITT), all randomized patients are included in the full analysis set. The primary efficacy evaluation of this study is based on the full analysis set.<br>• **Per Protocol Set (PPS):** All patients who have completed the treatment prescribed by the protocol or who have not significantly violated the protocol are included in the per protocol set. The exact definition of a serious protocol deviation will be finalized at the time of data auditing. PPS is a secondary analysis set for efficacy evaluation.<br>• **Safety Set (SS):** All randomized patients receiving more than one dose of the investigational product and one safety evaluation are included in the safety set. The safety evaluation of this study is based on the safety set.<br>**Statistical analysis**<br>Continuous variables were presented as median with interquartile range (IQR) and were compared with Wilcoxon test. Categorical variables were presented as frequency with proportion and were compared with chi-square test. Missing data on the primary outcome was imputed with treatment policy strategy, composite variable strategies, and while on treatment strategies accounting for intercurrent events.15 Group difference in the primary efficacy outcome was examined using chi-square test or Fisher exact test, and the corresponding 95% confidence intervals (CIs) of the difference between proportions were estimated based on the normal-approximation. Odds ratios (ORs) with 95% CIs were calculated

	using logistic regression.<br>Similar approaches were used for binary secondary outcomes, including mRS score ≤2 on day 90, NIHSS score ≤1 on day 14, 30, and 90, and safety outcomes on adverse events and treatment related adverse events. For mRS score on day 90, an ordinal logistic regression analysis was performed, with the results presented as common OR and 95% CI, where a common OR in favor of Y-2 sublingual tablet was >1.0. For changes in NIHSS score from baseline to day 14, means with 95% CIs were calculated for each group, and the mean differences with 95% CI between the groups were estimated by generalized linear regression. In addition, a post hoc sensitivity analysis was performed using different approaches to impute missing data on the primary efficacy outcome. Finally, the treatment effects on the primary outcome were analyzed among several prespecified subgroups by including the interaction between treatment and subgroup effect into the logistic regression model.<br>All the tests were 2 sided, and $P<0.05$ was considered statistically significant. Statistical analyses were performed with use of SAS software, version 9.4 (SAS Institute).

Abrreviations

**Abbreviations and terms**	**Interpretation**
AE	Adverse Events
BDNF	Brain-Derived Neurotrophic Factor
CRF	Case Report Form
CK	Creatine Kinase
CK-MB	Creatine Kinase-MB
DRQ	Date Rating Questionnaire
EC	Ethics Committees
ECG	Electrocardiogram
FAS	Full Analysis Set
FIB	Fibrinogen
GCP	Good Clinical Practice
HCG	Human Chorionic Gonadotropin
ICAM-1	Intercellular Adhesion Molecule 1
ICH	The International Council for Harmonisation of Technical Requirements for Pharmaceuticals for Human Use
IDMC	Independent Data Monitoring Committee
IL-6	Interleukin-6
IL-1β	Interleukin-1beta
iNOS	Inducible Nitric Oxide Synthase
ITT	Intention-To-Treat
MMP9	Matrix Metalloproteinase-9
mRS	Modified Rankin Scale
NDS	Neurological Disability Score
NIHSS	NIH Stroke Scale
PPS	Per Protocol Set
SAE	Serious Adverse Event
SAS	Statistical Analysis Software

SIS	Stroke Impact Scale
SS	Safety Set
TNF-α	Tumor Necrosis Factor-alpha
TC	Total Cholesterol
TG	Triglyceride

1. Introduction

1.1. Background

According to the report on stroke prevalence in China released by the Chinese Stroke Association for the first time in 2015, there are currently about 2.7 million new patients with cerebrovascular diseases in China every year, and about 1.3 million patients die of cerebrovascular diseases every year, namely, 1 new case with stroke every 12 seconds, and 1 death case of stroke every 21 seconds. Stroke is the first cause of death in China. The economic burden caused by stroke in China is up to 40 billion yuan every year, showing a rising trend[1]. According to the report of the China Stroke Conference in May 2016, stroke causes a disability rate of up to 75% in China. Stroke is characterized by high morbidity, high mortality and high disability rate. In addition, post-stroke cognitive dysfunction is also an important factor that seriously affects the quality of life and survival of patients. The incidence of stroke in China is increasing at an annual rate of 8.7%. It seriously endangers people's life, health and quality of life, and brings heavy disease burden to patients, their families and the society, which has become a major public health issue in China[2].

The existing therapies for acute ischemic stroke include venous thrombolytic therapy, endovascular interventional therapy (including intra-arterial thrombolysis, mechanical thrombectomy, angioplasty, and stent implantation), antiplatelet therapy, anticoagulant therapy, defibrinogen therapy, volume expansion therapy, and neuroprotective therapy[3].

Intravenous administration of recombinant tissue plasminogen activator (rt-PA) is the most well-documented in the treatment of acute ischemic stroke within 4.5 hours after attack. However, due to its relatively strict time window and the requirements for indications and contraindications, the proportion of patients receiving thrombolytic therapy is relatively small. In addition, a considerable number of patients with stroke due to large vessel occlusion are not sensitive to rt-PA, and only 6%-30% can achieve recanalization of occluded vessels, with limited benefit for these patients.

In recent years, large-scale international clinical studies have confirmed the efficacy and safety of endovascular thrombectomy in the treatment of acute stroke due to large vessel occlusion. However, the benefit of endovascular thrombectomy initiated 6 hours after the

attack is still uncertain. In addition, domestic and foreign guidelines still emphasize the importance of venous thrombolysis, and recommend venous thrombolysis in combination with endovascular thrombectomy. Endovascular intervention should be performed in hospitals with available conditions and low perioperative complications. Due to the great differences in medical conditions and levels in different regions in China, there are currently many restrictions on the promotion of equipment and techniques of mechanical thrombectomy, resulting in relatively large differences in treatment effects.

For most patients with acute ischemic stroke, clinical guidelines do not recommend indiscriminate anticoagulant therapy at early stage, as the efficacy of thrombin inhibitors in the treatment of acute ischemic stroke requires further confirmation by more studies, and it should only be used in clinical studies or on an individualized basis. Defibrinogen therapy is only used for cerebral infarction patients who are not suitable for thrombolysis and have been strictly screened, especially for patients with hyperfibrinogenemia. Volume expansion therapy is not recommended for patients with general ischemic stroke, while it can be considered for acute cerebral infarction caused by hypotension or cerebral hypoperfusion such as watershed infarction. However, volume expansion therapy should be noted that it may aggravate complications such as cerebral edema and heart failure, and it is not recommended for such patients. The efficacy and safety of neuroprotective agents such as edaravone, citicoline and piracetam still require further confirmation by more high-quality clinical trials. For ischemic stroke patients with no indications for thrombolysis and no contraindications, guidelines recommend antiplatelet therapy such as oral aspirin or clopidogrel as soon as possible after attack, but the therapeutic effect is limited. Although antiplatelet drugs can improve blood status and reduce the possibility of thrombosis, the effect on delaying neuronal apoptosis is limited.

In conclusion, thrombolytic therapy and endovascular therapy in acute ischemic stroke enable to restore blood flow and transport oxygen and nutrients to ischemic brain tissue, thereby reducing mortality and improving the prognosis of neurological function. However, due to its strict selection criteria, such as the time of onset of stroke, numerous contraindications in use and adverse reactions such as hemorrhagic transformation, there is

still a huge unmet clinical need for stroke treatment. It is necessary to conduct in-depth studies on the treatment measures and methods of stroke and develop safer and more effective new drugs for the stroke treatment. Drugs that directly target the brain parenchyma (such as neuroprotective agents) may be one of the feasible measures[4].

1.2. Background of the investigational product

1.2.1. Overview

Generic name: Y-2 Sublingual Tablets

English name: Y-2 Sublingual Tablets

Pinyin: Y-2 Shexiapian

Chinese chemical name: 3-甲基-1-苯基-2-吡唑啉-5-酮: (1R,2S,4R) -1,7,7-三甲基-二环[2.2.1]庚- 2-醇舌下片

English chemical name: 3-methyl-1- phenyl -2-pyrazolin-5-one (1R,2S,4R)-1,7,7-trimethylbicyclo[2.2.1]heptan-2-ol Sublingual Tablet

Chemical structural formula:

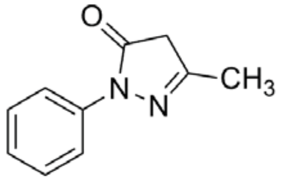

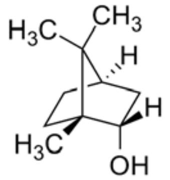

Molecular formula: $C_{10}H_{10}N_2O$ $C_{10}H_{18}O$

Molecular weight: 174.20 154.25

Description: A white or off-white tablet, with slight cryptic spot on the surface.

Dosage form and strength: Sublingual tablet; each contains 30 mg of edaravone and 6 mg of dexborneol.

Composition: Y-2 Sublingual Tablets is composed of edaravone, dexborneol and mannitol, microcrystalline cellulose, copovidone, polyvinylpolypyrrolidone, silicon dioxide, magnesium stearate and other excipients.

R&D progress: Y-2 Sublingual Tablets is an innovative drug developed by Yantai YenePharma Co., Ltd. with independent intellectual property rights. This product has applied for international patent PCT/CN2017/098620, and the patent has been authorized in China

(ZL 201780048512.7), Russia, Australia and Japan. Y-2 Sublingual Tablets was approved by the FDA for clinical trials in human (IND 138332) in March 2018, and the phase I clinical trial is underway (ClinicalTrials.gov Identifier: NCT03495206). The notice on clinical trials in China (CXHL1800183) was obtained on January 31, 2019, and a phase I clinical study of Y-2 Sublingual Tablets was carried out in Jiangsu Province Hospital. The results of single and multiple administration of Y-2 Sublingual Tablets in 50 healthy volunteers preliminarily demonstrated good clinical safety and tolerability of this product, and clarified the pharmacokinetics (PK) of the product in humans.

1.2.2. Mechanism of action

This product is a free radical scavenger and inflammatory protein expression inhibitor, which can scavenge hydroxyl free radicals (· OH), nitric oxide free radicals (NO·) and peroxynitrite ions (ONOO-), inhibit the expression of inflammation-related proteins such as tumor necrosis factor-α (TNF-α), interleukin-1β (IL-1β), cyclooxygenase-2 (COX-2) and inducible nitric oxide synthase (iNOS), reduce the damage of free radicals and inflammatory responses to brain tissues by scavenging excessive free radicals produced by brain tissues during ischemia and reperfusion and inhibiting secondary inflammatory responses, block the pathological changes caused by cerebral ischemia from two pathways, thereby exerting a synergistic therapeutic effect on cerebral ischemic injury. Preclinical efficacy studies in animals showed that the combination of edaravone and dexborneol could significantly improve acute brain injury in focal cerebral ischemia (reperfusion, permanent) and global cerebral ischemia-reperfusion model in rats, with good dose-effect relationship. In the focal cerebral ischemia-reperfusion model, the treatment time window was 8 h after ischemia, and the administration for 14 consecutive days could significantly improve the deficits of sensory, motor, learning and memory functions of animals after cerebral ischemia-reperfusion.

1.2.3. Preclinical study

1.2.3.1. Preclinical pharmacodynamics of Y-2 Sublingual Tablets

Pharmacodynamic study of Y-2 Sublingual Tablets in cerebral ischemia model

In the MCAO cerebral ischemia-reperfusion rat model, a single sublingual administration of Y-2 solution (edaravone: dexborneol = 5:1) at doses of 1, 3 and 9 mg/kg immediately after

2 h of ischemia significantly improved the score of neurological deficits and reduced the volume of cerebral infarction in rats 24 h after ischemia. The results of PK-PD study in the MCAO cerebral ischemia-reperfusion rat model showed that the edaravone and dexborneol exposure (C_{max} and $AUC_{0\text{-}inf}$) increased proportionally with dose increase after sublingual administration of Y-2 solution. The effective dose of Y-2 in the MCAO rat model was 1 mg/kg, and the $AUC_{0\text{-}inf}$ of edaravone and dexborneol corresponding to the dose was 1050 h·ng/mL and 54.2 h·ng/mL, respectively. For human with an average body weight of 70 kg, the effective human equivalent dose (HED) of Y-2 Sublingual Tablets for ischemic stroke is approximately 12 mg.

<u>Pharmacodynamic study of Y-2 Sublingual Tablets in intracerebral hemorrhage model</u>

In the collagenase IV-induced intracerebral hemorrhage (ICH) rat model, Y-2 solutions (edaravone : dexborneol = 5:1) at doses of 3 and 6 mg/kg were administered sublingually immediately after injection of collagenase IV, and repeated doses were given 6 h, 24 h, 30 h, 48 h and 54 h after surgery. The cerebral water content in the hemisphere on the bleeding side was reduced significantly. Sublingual administration of Y-2 solution at doses of 1, 3, and 6 mg/kg showed significant protection against ICH-induced blood-brain barrier damage. The results of PK-PD study in the ICH rat model showed that the edaravone and dexborneol exposure (C_{max} and $AUC_{0\text{-}inf}$) increased proportionally with dose increase after sublingual administration of Y-2 solution. The effective dose of Y-2 Sublingual Tablets in the ICH rat model was 3 mg/kg (containing 2.4 mg/kg edaravone and 0.6 mg/kg dexborneol), and the $AUC_{0\text{-}inf}$ of edaravone corresponding to the dose was 2420 h·ng/mL (first dose) and 2680 h·ng/mL (last dose, 54 h), the $AUC_{0\text{-}inf}$ of dexborneol corresponding to the dose was 77.8 h·ng/mL (first dose) and 34.8 h·ng/mL (last dose, 54 h). For human with an average body weight of 70 kg, the effective human equivalent dose (HED) of Y-2 Sublingual Tablets for hemorrhagic stroke is approximately 36 mg.

In the long-term efficacy study in ICH-induced brain injury rat model, sublingual administration of 1, 3 and 6 mg/kg of Y-2 solution (edaravone: dexborneol = 5:1) for 14 consecutive weeks significantly improved ICH-induced motor and sensory dysfunction and had a protective effect on the survival of striatal neurons.

1.2.3.2. Preclinical toxicology of Y-2

Safety pharmacology

The safety pharmacology of Y-2 Sublingual Tablets was evaluated by the effect of intravenous administration of edaravone dexborneol on the central nervous system, cardiovascular and respiratory systems, and the changes in body temperature during intravenous injection in mice or beagles.

The effect of intravenous administration of edaravone dexborneol on the central nervous system in mice was evaluated by exploring the general behaviors, spontaneous activities, synergistic effect of subliminal hypnotic dose of pentobarbital, and rotational coordination function. No general behavioral abnormalities were found in all dose groups (normal saline, edaravone dexborneol low-dose group (LD, 6.4 mg/kg edaravone and 1.6 mg/kg dexborneol), medium-dose group (MD, 20 mg/kg edaravone and 5 mg/kg dexborneol), and high-dose group (HD, 60 mg/kg edaravone and 15 mg/kg dexborneol) and solvent control group after single single intravenous dose. There was a slight dose-dependent reduction in spontaneous activities in all dose groups. Except for a slight decrease in movement time and distance in the solvent control group, no abnormalities in rotational coordination were observed in other dose groups. Prolonged sleep duration or shortened sleep latency was observed in the HD group, while shortened sleep duration or prolonged sleep latency was observed in the LD, MD and solvent control groups. Positive or negative synergistic effects of subthreshold hypnotic dose of pentobarbital were observed.

The safety of edaravone dexborneol in dogs was evaluated by observing cardiovascular and respiratory function and body temperature. A slight, transient dose-dependent increase in blood pressure was observed after single intravenous injection of edaravone dexborneol 25 mg/kg (20 mg/kg edaravone and 5 mg/kg dexborneol), 50 mg/kg (40 mg/kg edaravone and 10 mg/kg dexborneol), 100 mg/kg (80 mg/kg edaravone and 20 mg/kg dexborneol) or control solvent, and the blood pressure returned to normal within 60 minutes. No significant changes in heart rate, P, R, T wave, PR, QRS and QT interval, ST segment, respiratory rate, tidal volume and body temperature in each group were observed compared with those before administration.

In conclusion, decreased spontaneous activities in mice, positive or negative synergistic effects of subthreshold hypnotic dose of pentobarbital and slight transient increase in blood pressure in dogs were observed after single intravenous injection of edaravone dexborneol, and no significant respiratory or temperature risks were observed in dogs.

1.2.3.3. Toxicology

Summary of toxicology of edaravone dexborneol injection

The results of bridging pharmacokinetic study in dogs and tissue distribution study in rats indicated that after sublingual administration of Y-2 Sublingual Tablets and intravenous injection of edaravone dexborneol, the systematic exposure of edaravone and dexborneol was similar, and there was no significant difference in tissue distribution between the two. However, due to the dose limitation of sublingual administration, toxicology of Y-2 Sublingual Tablets was mainly evaluated by toxicological studies of intravenous administration of edaravone dexborneol. At the same time, tests of 2-week repeated-dose oral irritation of Y-2 Sublingual Tablets in dogs and 4-week repeated-dose toxicity in dogs (maximum dose: 3 tablets/dog) were conducted.

The species of animals used in toxicological studies (rats and dogs) are selected based on their similarity to human metabolic characteristics. Metabolites sulfate and glucuronic acid conjugates of edaravone have also been detected in the plasma of rats, dogs and humans (FDA, NDA209176). In *in vitro* liver microsomal studies, the metabolite of dexborneol, glucuronic acid conjugate, is identical in all species (mice, rats, dogs, monkeys, and humans).

In SD rats, the NOAEL of single intravenous injection of 25, 50, or 100 mg/kg edaravone dexborneol (4:1) was considered to be 25 mg/kg (20 mg/kg edaravone and 5 mg/kg dexborneol); the NOAEL of 28-day (4-week) repeated intravenous injection of 10, 20, or 40 mg/kg/day edaravone dexborneol (4:1) followed by a 2-week recovery period was 20 mg/kg/day (16 mg/kg edaravone and 4 mg/kg dexborneol); $AUC_{0\text{-}inf}$ of edaravone and dexborneol after the last dose on Day 28 was 6600 ng·h/mL (♂) and 485 ng·h/mL (♂), respectively, equivalent to ~6 and ~9 times of the expected exposure of 12 mg of Y-2 Sublingual Tablets in human for the treatment of ischemic stroke, respectively, and ~3 and ~6

times of the expected exposure of 36 mg (recommended initial dose for human) of Y-2 Sublingual Tablets in humans for the treatment of hemorrhagic stroke, respectively.

In dogs, the NOAEL of single intravenous injection of 50, 100, or 200 mg/kg edaravone dexborneol (4:1) was considered to be 50 mg/kg (40 mg/kg edaravone and 10 mg/kg dexborneol); the NOAEL of 28-day (4-week) repeated intravenous injection of 25, 50, or 100 mg/kg/day edaravone dexborneol (4:1) followed by a 2-week recovery period was 50 mg/kg/day (40 mg/kg edaravone and 10 mg/kg dexborneol); $AUC_{0\text{-inf}}$ of edaravone and dexborneol after the last dose on Day 28 was 89400 ng·h/mL (♂) and 1170 ng·h/mL (♂), respectively, equivalent to ~85 and ~21 times of the expected exposure of 12 mg of Y-2 Sublingual Tablets in humans for the treatment of ischemic stroke, respectively, and ~37 and ~15 times of the expected exposure of 36 mg (recommended initial dose for human) of Y-2 Sublingual Tablets in humans for the treatment of hemorrhagic stroke, respectively. Microscopic abnormalities of the kidneys were observed in dose groups above 100 mg/kg (edaravone at a dose of 80 mg/kg and dexborneol at a dose of 20 mg/kg) in acute and repeated-dose toxicity studies. These abnormalities were reversible during the 2-week recovery period in repeated-dose toxicity studies.

No abnormal clinical symptoms were observed after single intravenous injection of dexborneol at doses of 5, 10, or 20 mg/kg in rats or after acute intravenous injection of dexborneol at doses of 12.5, 25, or 50 mg/kg in dogs. No abnormal clinical symptoms were observed during the 28-day repeated intravenous administration of dexborneol at a dose of 8 mg/kg/day in rats or 28-day repeated intravenous administration of dexborneol at a dose of 20 mg/kg/day in dogs, which was equivalent to the high dose level of edaravone dexborneol used in rats and dogs.

In multiple toxicity studies, no significant toxicities, including acute toxicity, repeated-dose toxicity, reproductive toxicity, genetic toxicity, and local toxicity, were observed in any target organ after intravenous administration of edaravone dexborneol. Toxicological test results suggested that intravenous edaravone dexborneol was safe and well tolerated in selected animals.

Edaravone dexborneol (4:1) did not cause gene mutation or chromosome damage in two *in vitro* tests (microbial mutation test, lung fibroblast test in Chinese hamsters) and a micronucleus test of intravenous administration in mice *in vivo*.

Reproductive and developmental toxicity of Y-2 Sublingual Tablets was assessed via the study of reproductive toxicity of intravenous administration of edaravone dexborneol (4:1) in rats and study of embryonic developmental toxicity of intravenous administration of edaravone dexborneol (4:1) in pregnant rats and rabbits. Edaravone dexborneol at doses of 6.25, 12.5 or 25 mg/kg/day was administrated intravenously in SD rats. Male mice were administered for 28 days and female mice were administered for 14 days before being put in the same cage, then male mice were administered continuously until successful mating and female mice were administered continuously until Day 6 of pregnancy after being put in the same cage. No obvious abnormalities or toxicities in fertility and early embryonic development were observed. Similar body weight and slight reduction in food intake were observed in animals in all dose groups of intravenous edaravone dexborneol. Pregnant SD rats on Day 6 to Day 15 of pregnancy were intravenously injected with edaravone dexborneol at doses of 6.25, 12.5 or 25 mg/kg/d for 10 consecutive days, and were dissected for autopsy before delivery. No embryo-fetal development toxicity was observed. Pregnant rabbits on Day 6 to Day 18 of pregnancy were intravenously injected with normal saline or edaravone dexborneol at doses of 2.5, 5 or 10 mg/kg/d for 13 consecutive days. No parental or embryo-fetal developmental toxicity was observed in all dose groups.

In addition, Y-2 Sublingual Tablets were sublingually administered in beagles for 4 consecutively weeks followed by a 28-day recovery period, qd, 3 tablets/time (36 mg/tablet, i.e., edaravone at a dose of 90 mg/day and dexborneol at a dose of 18 mg/day) based on the dosage form of Y-2 Sublingual Tablets, and no obvious toxic reactions were observed; In a test of oral mucosal irritation of Y-2 Sublingual Tablets in dogs, no significant irritation was observed after 14 consecutive days of sublingual administration of Y-2 Sublingual Tablets (qd, 3 tablets/time) in beagles. In conclusion, Y-2 Sublingual Tablets is safe and well tolerated in dogs.

Systematic exposure

The detailed critical results of the study of 4-week repeated intravenous administration of edaravone dexborneol in SD rats and beagles, as well as systematic exposure to edaravone and dexborneol, are shown in Tables 1.2-1 and 1.2-2. The detailed key findings of the study of 4-week repeated administration of Y-2 Sublingual Tablets in beagles, as well as systematic exposure to edaravone and dexborneol, are shown in 1.2-3.

Table 1.2- 1 Key findings and systematic exposure in the study of 4-week repeated-dose toxicity in SD rats

Daily dose (mg/kg)		Normal saline (0.9% NaCl)		Solvent (6.4% propylene glycol)		Dexborneol		Edaravone dexborneol (Edaravone: dexborneol = 4:1)					
						8		10		20 (NOAEL)		40	
Number of animals		M 15	F 15	M 15	F 15	M 15	F 15	M 15	F 15	M 15	F 15	M 15	F 15
Toxicokinetics: AUC (ng·h/mL)													
Day 1	Edaravone	/		/	/	/	/	3210	4260	7490	9390	16400	18300
	Dexborneol	/	/	/	/	682	711	175	201	400	452	855	1030
Day 28	Edaravone	/	/	/	/	/	/	4440	5390	6600	9790	21500	25000
	Dexborneol	/	/	/	/	966	779	282	204	485	398	1120	852
Significant findings		/	/	Decreased RBC and HGB, increased reticular cells		/	/	/	/	/	/	Decreased RBC and HGB, increased reticular cells	
Near death or death		N/A											

Decreased body weight gain	No significant changes were observed in all groups compared with the negative control group.
Food intake	No significant changes in food intake were observed in all groups in the study.

Table 1.2- 2 Key findings and systematic exposure in the study of 4-week repeated-dose toxicity in beagles

Daily dose (mg/kg)		Normal saline (0.9%NaCl)		Solvent (8% propylene glycol)		Dexborneol		Edaravone dexborneol (Edaravone: dexborneol = 4:1)					
						20		25		50 (NOAEL)		100	
Number of animals		M15	F15	M15	F15	M15	F15	M15	F15	M15	F15	M15	F15
Toxicokinetics: AUC (ng·h/mL)													
Day 1	Edaravone	/	/	/	/	/	/	39700	39800	70100	90800	124000	232000
	Dexborneol	/	/	/	/	2550	2160	353	351	1090	1020	3130	3170
Day 28	Edaravone	/	/	/	/	/	/	41800	58900	89400	92000	153000	194000
	Dexborneol	/	/	/	/	1870	2250	455	408	1170	1170	3020	2870

Significant findings	Slightly reduced body weight gain and/or food intake in solvent control (8% propylene glycol) group or 100 mg/kg edaravone dexborneol group; decreased RBC and HGB, and decreased ratio of granulocytes to red blood cells; increased reticular cells and/or PLT.<br>All these symptoms were reversible within 2 weeks of recovery after medication (not noticeable or undetected in animals).										
Near death or death	/	/	/	/	/	/	/	/	/	/<br>/	One animal was euthanized on Day 14.
Decreased body weight gain	Compared with the negative control group, no significant changes in body weight in animals in the 25, 50, or 100 mg/kg/day test groups.<br>The animals in the 100 mg/kg group showed a slight trend of decreased body weight gain during the medication.										
Food intake	Except for a significant reduction in food intake related to the treatment (recovered during the subsequent 2-week drug discontinuation period) in the 100 mg/kg group during the treatment, no food intake related to the treatment was found in other animals.										

Table 1.2- 3 Key findings and systematic exposure in the study of 4-week repeated-dose toxicity of Y-2 Sublingual Tablets in beagles

Group	Sublingual blank tablets		Y-2 Sublingual Tablets		Edaravone dexborneol	
Dose	0		108mg<br>(90 mg of edaravone and 18 mg of dexborneol)		112.5mg<br>(90 mg of edaravone and 22.5 mg of dexborneol)	
	0		11.21 mg/kg edaravone	1.87 mg/kg dexborneol	11.60 mg/kg edaravone	2.9 mg/kg dexborneol
Method of administration	Sublingual administration		Sublingual administration		Intravenous infusion	
Number of animals	M	F	M	F	M	F

		5	5	5	5	5	5
Toxicokinetics: AUC0-t (ng·h/mL)							
Day 1	Edaravone	/	/	24830	36180	41073	49880
	Dexborneol	/	/	140	130	549	584
Day 28	Edaravone	/	/	24300	41350	32620	41070
	Dexborneol	/	/	93	127	536	575
Significant findings		None		None		After the first dose, ALT in 7/10 dogs exceeded the overall fluctuation range of the adaptive phase, AST in 6/10 dogs and T.BIL in 3/10 dogs with T. BIL exceeded the upper limit of the overall fluctuation range of the adaptive phase. After the completion of administration, AST in 2/10 and T.BIL in 5/10 animals exceeded the upper limit of the overall fluctuation range of their adaptive phases. The ALT, AST, and T.BIL levels did not continue to increase with the prolonged administration time (repeated dose for 28 consecutive days), but increased and then decreased and remained at a relatively high level, and recovered after 2 weeks.	

1.2.3.4. Non-clinical pharmacokinetics of Y-2 Sublingual Tablets

To systematically elucidate the ADME of Y-2 Sublingual Tablets, pharmacokinetic studies of intravenous injection of edaravone, dexborneol, edaravone dexborneol, and sublingual administration of Y-2 Sublingual Tablets (or Y-2 solution) are summarized in this Section, including absorption, distribution, metabolism and excretion of intravenous injection of edaravone dexborneol, edaravone and dexborneol in rats; distribution and excretion of Y-2 solution (Considering that it was not easy to administrate Y-2 Sublingual Tablets in rats, sublingual administration of Y-2 solution was given for the study of tissue distribution and excretion) in rats; pharmacokinetic study of single dose or 7-day repeated doses of intravenous administration of edaravone dexborneol, edaravone, dexborneol and sublingual administration of Y-2 Sublingual Tablets in dogs; bridging pharmacokinetic study of intravenous administration of edaravone dexborneol and sublingual administration of Y-2 Sublingual Tablets in dogs; plasma protein binding rate; metabolism of edaravone and dexborneol; inhibition of edaravone dexborneol and dexborneol on major hepatic CYPs in humans and induction on hepatic CYPs in rats.

PK and absorption

In rats, after single intravenous injection of edaravone at doses of 0.6, 1.2, 2.4, or 7.2 mg/kg, the edaravone $t_{1/2}$ was 0.57, 1.54, 1.49, or 1.76 h, respectively, and the edaravone $AUC_{0\text{-}inf}$ was 827.7, 2,383.5, 3,878.7, or 10,759.9 ng·h/mL, respectively. After single intravenous injection of dexborneol at dose of 0.15, 0.3, 0.6, or 1.8 mg/kg, the dexborneol $t_{1/2}$ in all dose groups was less than 30 minutes, and the dexborneol $AUC_{0\text{-}inf}$ was 12.5, 31.1, 65.9, or 194.0 ng·h/mL, respectively. After single intravenous injection of edaravone dexborneol (4:1) at doses of 0.75, 1.5, 3.0, or 9.0 mg/kg, the edaravone $t_{1/2}$ was 0.59, 1.48, 1.42, or 1.71 h, respectively, and the edaravone $AUC_{0\text{-}inf}$ was 784.6, 2,103.4, 3,636.2, or 11,743.0 ng·h/mL, respectively; the dexborneol $t_{1/2}$ in all dose groups was less than 30 minutes, and the dexborneol $AUC_{0\text{-}inf}$ was 18.8, 38.1, 73.3, or 246.6 ng·h/mL, respectively. The edaravone and dexborneol exposure ($AUC_{0\text{-}inf}$) following the intravenous injection of edaravone, dexborneol, or edaravone dexborneol was proportional to dose increase. The dexborneol and edaravone in edaravone dexborneol had no effect on the PK properties of either dexborneol or edaravone.

In dogs, after single intravenous injection of edaravone at doses of 2.4, 4.8, or 9.6 mg/kg, the edaravone $t_{1/2}$ was 8.79, 9.63, or 9.57 h, respectively, and the edaravone $AUC_{0\text{-}inf}$ was 30,977.6, 51,648.3, or 65,479.8 ng·h/mL, respectively. After single intravenous injection of dexborneol at doses of 0.6, 1.2, or 2.4 mg/kg, the dexborneol $t_{1/2}$ was 0.64, 0.90, or 0.92 h, respectively, and the dexborneol $AUC_{0\text{-}inf}$ was 99.8, 197.9, or 501.2 ng·h/mL, respectively. After single intravenous injection of edaravone dexborneol (4:1) at doses of 0.75, 1.5, 3.0, or 9.0 mg/kg, the edaravone $t_{1/2}$ was 9.48, 9.55, or 9.97 h, respectively, and the edaravone $AUC_{0\text{-}inf}$ was 28,341.4, 48,593.2, or 60,077.6 ng·h/mL, respectively; the dexborneol $t_{1/2}$ was 0.67, 0.86, or 1.05 h, respectively, and the dexborneol $AUC_{0\text{-}inf}$ was 114.9, 207.3, or 432.1 ng·h/mL, respectively. After 7-day repeated intravenous injection of edaravone (4.8 mg/kg), dexborneol (1.2 mg/kg), or edaravone dexborneol (6 mg/kg, 4:1), the edaravone $t_{1/2}$ was 8.65, N/A, or 8.13 h, respectively, and the edaravone $AUC_{0\text{-}inf}$ was 45,906.9, N/A, or 41,614.2 ng·h/mL, respectively; the dexborneol $t_{1/2}$ was N/A, 1.33, or 1.4 h, and the dexborneol $AUC_{0\text{-}inf}$ was N/A, 298.8, or 248.2 ng·h/mL, respectively. The cumulative coefficient ($AUC_{0\text{-}inf}$ ratio from Day 7 to Day 1) of dexborneol in dexborneol and edaravone dexborneol was 1.51 and 1.20, respectively, and the cumulative coefficient of edaravone in edaravone and edaravone dexborneol was 0.89 and 0.86, respectively. A dose-dependent increase in mean dexborneol $AUC_{0\text{-}inf}$ was observed in dogs following single or repeated 7-day administration, whereas not detected in the high-dose edaravone group. In the experiment of single dose or 7-day repeated doses, there was no significant difference in the PK properties of edaravone and dexborneol in edaravone, or dexborneol and edaravone dexborneol. The dexborneol and edaravone in edaravone dexborneol had no effect on the PK properties of either dexborneol or edaravone.

In the bridging pharmacokinetic study of sublingual administration of edaravone dexborneol (5:1) and intravenous injection of edaravone dexborneol (4:1), after intravenous injection of edaravone dexborneol (30 mg, including 24 mg edaravone and 6 mg dexborneol) or sublingual administration of Y-2 Sublingual Tablets (1 tablet. 36 mg, including 30 mg edaravone and 6 mg dexborneol), edaravone and dexborneol showed similar pharmacokinetic parameters (C_{max}, $AUC_{0\text{-}t}$, $AUC_{0\text{-}inf}$, $t_{1/2}$, MRT) in male and female dogs. After single

administration of Y-2 Sublingual Tablets (1 tablet), the t_{max} of edaravone and dexborneol was 0.63 h and 0.35 h, respectively, and the bioavailability was 70% and 85%, respectively.

After single administration of 36 mg (1 tablet) of Y-2 Sublingual Tablets, 72 mg (2 tablets), or 108 mg (3 tablets), the edaravone exposure (C_{max} and $AUC_{0\text{-}inf}$) increased in a dose-dependent manner. No gender differences were observed for any of the pharmacokinetic parameters, except for inter-individual variations in C_{max} (♂89.5 ng/mL vs ♀211 ng/mL) and $AUC_{0\text{-}inf}$ (♂58.2 ng·h/mL vs ♀110 ng·h/mL) of dexborneol in the 36 mg Y-2 (1 tablet) dose group, which may be due to the limited number of animals (3 males and 3 females). After 7-day repeated doses of 72 mg (2 tablets) of Y-2 Sublingual Tablets, the mean $AUC_{0\text{-}inf}$ of edaravone and dexborneol on Day 7 was approximately 1.17 and 1.05 times that on Day 1, respectively, indicating that multiple doses had no accumulation potential.

Distribution

Plasma protein binding rate

The in vitro plasma protein binding rates of edaravone and dexborneol were approximately 70% and 69% in rats, 35% and 77% in dogs, and 75.5% and 68% in humans, respectively. The plasma protein binding rate of edaravone showed species difference and reached saturation at 50,000 ng/mL. No drug interactions were found in the plasma protein binding rates of edaravone and dexborneol.

Tissue distribution

Tissues were collected 5 min, 20 min, or 2 h after intravenous injection of edaravone (2.4 mg/kg), dexborneol (0.6 mg/kg), or edaravone dexborneol (3.0 mg/kg, 4:1) for a comparative study of tissue distribution. The study showed that edaravone or dexborneol had no effect on the tissue distribution of each other in rats.

In SD rats, after single intravenous injection of edaravone dexborneol (6 mg/kg, 4:1) or single sublingual administration of dexborneol (6 mg/kg, 5:1, i.e., Y-2 solution), edaravone and dexborneol were rapidly distributed and cleared in each tissue. After intravenous or sublingual administration, edaravone was higher in liver and kidney, while dexborneol was higher in kidney, brain, and adipose tissue. Edaravone and dexborneol had no significant accumulation in important tissues. There was no significant distribution difference in

important tissues such as heart, liver, spleen, lung, kidney, and brain after intravenous injection of edaravone dexborneol (4:1) and sublingual administration of edaravone dexborneol (5:1).

Metabolism

In vitro biotransformation

In vitro primary biotransformation of dexborneol to glucuronic acid conjugation and forming of glucuronic acid conjugate (B-M1) is identical in all species (mice, rats, dogs, monkeys, and humans), which can also be detected in vivo. None of the recombinant human CYPs or UGTs systems constructed in vitro, including rCYP1A2, 2B6, 2C8, 2C9, 2C19, 2D6 or 3A4 and rUGT1A1, 1A3, 1A4, 1A6, 1A9, 2B7 or 2B15, and other metabolic enzymes were involved in the glucuronic acid conjugation of dexborneol.

In vivo metabolism

The Phase II metabolites of edaravone, glucuronic acid conjugate (E-M1) and sulfate conjugate (E-M2), were detected in urine and bile samples of rats after intravenous injection of edaravone dexborneol (edaravone (2.4 mg/kg) and dexborneol (0.6 mg/kg)). Glucuronic acid conjugate (B-M1) of dexborneol was detected in urine and bile samples, and traces of camphor (B-M2) were detected in plasma in toxicokinetic study. No other metabolites of edaravone or dexborneol were found in rats. Data from the clinical review of Radicava® (edaravone injection, FDA, NDA 209176) showed that the metabolic properties of edaravone were similar in Japanese and Caucasian populations, with sulfate followed by glucuronic acid conjugate being major metabolites in plasma and glucuronic acid conjugate followed by sulfate being major metabolites in urine.

Excretion

In rats, after single intravenous injection of edaravone (2.4 mg/kg) or edaravone dexborneol (edaravone (2.4 mg/kg) and dexborneol (0.6 mg/kg)), the 72-hour cumulative urinary excretion rate of edaravone was 51.8% and 47.9%, respectively. The 24-hour cumulative bile excretion rate of edaravone and glucuronic acid conjugate was 28.6% and 34.6%, respectively. No edaravone or glucuronic acid conjugate was detected in fecal samples. There was no significant difference in the excretion rate of edaravone after administration of

edaravone and edaravone dexborneol. Dexborneol had no significant effect on the excretion of edaravone.

After single intravenous injection of dexborneol (0.6 mg/kg) or edaravone dexborneol (3 mg/kg, including edaravone (2.4 mg/kg) and dexborneol (0.6 mg/kg)), the 72-hour cumulative urinary excretion rate of dexborneol was 12.1% and 15.1%, respectively; the 4-hour cumulative bile excretion rate was 0.043% and 0.029%, respectively, and the 72-hour cumulative fecal excretion rate of was 8.3% and 6.1% There was no significant difference in the excretion rate of dexborneol in animals administered with dexborneol or edaravone dexborneol.

After single sublingual administration of edaravone dexborneol (6.0 mg/kg, including edaravone (5 mg/kg) and dexborneol (1 mg/kg)), edaravone was excreted mainly in the urine in the form of proto type and glucuronic acid conjugate (E-M1). The 72-hour total cumulative excretion rate of edaravone and glucuronic acid conjugate was about 29%. Dexborneol was excreted mainly in urine and bile as glucuronic acid conjugate (B-M1) and in feces as proto type. The total cumulative excretion rate of dexborneol and B-M1 was about 35.7%.

Pharmacokinetic drug interactions

CYP inhibition and induction

The results of in vitro enzymatic study showed that edaravone, dexborneol, and edaravone dexborneol (4:1) had no significant inhibitory effect on five major CYP450 subtypes (CYP1A2, CYP2C9, CYP2C19, CYP2D6, and CYP3A4). The IC50 of the three drugs was all ≥100 μM. At the animal level, respective intravenous injection of the three drugs did not induce CYP1A2, CYP2C9, CYP2C19, and CYP3A4 in SD rats.

1.2.3.5. Clinical pharmacokinetics

The Phase I clinical study of Y-2 Sublingual Tablets was conducted in healthy adult participants. The study was divided into single-ascending dose trial (1 tablet, 2 tablets, and 3 tablets, 10 participants in each group) and multiple-ascending dose trial (1 tablet, 2 tablets, 10 participants in each group) to evaluate the safety, tolerability and pharmacokinetic profile of Y-2 Sublingual Tablets in healthy adult male and female participants.

After single sublingual administration of Y-2 Sublingual Tablets at dose of 1 tablet (containing 30 mg of edaravone + 6 mg of dexborneol), 2 tablets (containing 60 mg of edaravone + 12 mg of dexborneol), and 3 tablets (containing 90 mg of edaravone + 18 mg of dexborneol), edaravone and dexborneol were absorbed rapidly in the body. With the increase of dose, the C_{max} and exposure AUC of edaravone in plasma increased accordingly; The increase of C_{max} showed a rough but atypical dose-proportional increase, and the exposure AUC_{last} and AUC_{inf} did not show a typical dose-exposure proportional relationship, with an increase slightly greater than the proportional increase in dose. With the increase of the administered dose, dexborneol in plasma did not increase significantly, showing a saturated trend; In the dose range of 6-18 mg, C_{max} and AUC did not show a dose-exposure proportional relationship, both of which were less than the proportional increase of dose.

After Y-2 Sublingual Tablets was administered twice a day (BID) for 9 consecutive days at dose of 1 tablet (containing 30 mg of edaravone + 6 mg of dexborneol) and 2 tablets (containing 60 mg of edaravone + 12 mg of dexborneol), edaravone and dexborneol in plasma basically reached a stable state. After continuous administration, there was no significant accumulation of edaravone and dexborneol in plasma, and the AUC accumulation ratios of edaravone and dexborneol exposure were 1.05±0.0995 (0.708~1.19) and 1.35±0.364 (0.596~1.85), respectively.

A total of 33 adverse events and 4 adverse reactions occurred during the single-dose and multiple-dose trial. The incidence of adverse events and adverse reactions was not dose-dependent, and the outcome of adverse reactions was either remission of symptoms or return to baseline levels.

Table 1.2-4. Pharmacokinetic Parameters of Edaravone in Healthy Adult Participants after Single Administration of Y-2 Sublingual Tablets

PK parameter	1 tablet	2 tablets	3 tablets
	N=10	N=10	N=10
$AUC_{0-\infty}$ (h*ng/mL)	5290±2080	13200±3970	20500±3900
AUC_{0-t} (h*ng/mL)	5160±2040	13000±3970	20300±3910

C_{max} (ng/mL)	2110±756	4270±1320	6330±1070
$t_{1/2}$ (h)	2.87±0.67	3.64±0.61	3.67±0.51
T_{max} (h)（Min，Max）	1.00（0.75, 1.00）	1.00（1.00, 2.00）	1.25 (0.75, 1.50)
V_z/F (L)	25.8±8.08	25.3±5.85	23.9±5.69
CL/F（L/h）	6.54±2.51	5.00±1.68	4.55±0.95

Table 1.2-5. Pharmacokinetic Parameters of Dexborneol in Healthy Adult Participants after Single Administration of Y-2 Sublingual Tablets

PK parameter	1 tablet	2 tablets	3 tablets
	N=10	N=10	N=10
$AUC_{0-\infty}$ (h*ng/mL)	30.8±12.1	36.1±9.89	49.6±15.9
AUC_{0-t} (h*ng/mL)	26.7±12.9	31.9±12.4	46.5±15.0
C_{max} (ng/mL)	20.0±8.61	24.4±11.5	35.1±19.9
$t_{1/2}$ (h)	2.40±1.71	1.64±0.537	3.16±0.895
T_{max} (h)（Min，Max）	0.50（0.25, 0.75）	0.50（0.50, 1.00 ）	0.625 (0.167, 0.75)
V_z/F (L)	738±561	888±504	1720±382
CL/F（L/h）	226±95.2	366±145	399±128

Table 1.2-6 Pharmacokinetic Parameters of Edaravone in Healthy Adult Participants after Multiple Administration

PK parameter	1 tablet (first time)	1 tablet (last time - 9th time)
	N=10	N=10
AUC_{0-t} (h*ng/mL)	6170±2550	7000±3330
$AUC_{0-\infty}$ (h*ng/mL)	6500±2810	7130±3360
AUC_{0-12h} (h*ng/mL)	6170±2550	6510±2830
C_{max} (ng/mL)	2470±840	2520±721
$t_{1/2}$ (h)	2.89±0.46	3.76±1.03
T_{max} (h)（Min，Max）	1.00（0.75, 2.00）	1.00 (0.50, 1.00)
V_z/F (L)	21.8±7.72	27.0±8.26
CL/F（L/h）	5.41±2.16	5.32±1.91

AUC accumulation index		1.06
PK parameter	2 tablets (first time)	2 tablets (last time - 9th time)
	N=10	N=10
AUC_{0-t} (h*ng/mL)	11200±2130	12600±2690
$AUC_{0-\infty}$ (h*ng/mL)	11600±2270	12800±2720
AUC_{0-12h} (h*ng/mL)	11200±2130	11700±2390
C_{max} (ng/mL)	4210±733	3950±686
$t_{1/2}$ (h)	2.49±0.24	4.36±0.34
T_{max} (h)（Min，Max）	1.00（0.75, 2.00）	1.50 (1.00, 2.00)
V_z/F (L)	19.1±2.93	33.6±8.75
CL/F（L/h）	5.36±1.05	5.31±1.10
AUC accumulation index		1.04

Table 1.2-7 Pharmacokinetic Parameters of Dexborneol in Healthy Adult Participants after Multiple Administration

PK parameter	1 tablet (first time)	1 tablet (last time - 9th time)
	N=10	N=10
AUC_{0-t} (h*ng/mL)	22.5±8.47	42.7±14.2
$AUC_{0-\infty}$ (h*ng/mL)	26.0±9.27	39.5±18.3
AUC_{0-12h} (h*ng/mL)	24.1±9.02	37.1±10.5
C_{max} (ng/mL)	22.5±19.4	25.9±10.7
$t_{1/2}$ (h)	2.72±0.887	4.02±1.13
T_{max} (h)（Min，Max）	0.50（0.50, 0.75）	0.25 (0.25, 0.50)
V_z/F (L)	988±396	1210±829

CL/F（L/h）	260±98.2	176±61.1
AUC accumulation index		1.54
PK parameter	2 tablets (first time)	2 tablets (last time - 9th time)
	N=10	N=10
$AUC_{0\text{-}t}$ (h*ng/mL)	36.6±11.4	51.9±18.8
$AUC_{0\text{-}\infty}$ (h*ng/mL)	40.2±12.9	64.8±28.5
$AUC_{0\text{-}12h}$ (h*ng/mL)	39.0±13.1	45.3±14.3
C_{max} (ng/mL)	23.7±13.3	26.2±15.2
$t_{1/2}$ (h)	4.01±1.66	7.90±4.12
T_{max} (h)（Min，Max）	0.75（0.25， 1.00）	0.50 (0.50, 0.75)
V_z/F (mL)	1710±376	2727±1169
CL/F（mL/h）	325±93.1	284±71.6
AUC accumulation index		1.25

1.3. Risk

Based on the available evidence, this product has potential clinical efficacy in improving prognostic neurological function in patients with acute ischemic stroke. It was confirmed in the phase III trial (SIM-23-02 trial) that the clinical efficacy of concentrated solution of edaravone dexborneol for injection, which has similar active ingredients of this product, was significantly superior to that of edaravone injection. The results of the SIM-23-02 trial showed that the proportion of participants with mRS score ≤ 1 on Day 90 in the group of concentrated solution of edaravone dexborneol for injection (65.61%) was much higher than that in the group of edaravone injection (57.48%). The difference between the two groups was 8.13%, and the 95% CI was (2.63% ~ 13.63%, P = 0.0039). The mean differences from baseline in NIHSS scores were -2.97, -3.99, and -4.81 respectively on days 14, 30, and 90 after treatment in the edaravone dexborneol group, and -2.56, -3.60, and -4.49 respectively in the edaravone group. The differences from baseline were statistically significant in both

groups. The mean differences of decrease in NIHSS scores between the edaravone dexborneol group and the edaravone group on days 14, 30, and 90 were -0.41 (95%CI: -0.73~ -0.09, P = 0.0097), -0.39 (95%CI: -0.71~ -0.07, P = 0.0151), and -0.32 (95%CI: -0.64 ~ -0.01, P = 0.0370), respectively. The differences between the two groups were statistically significant.

When edaravone was approved for marketing in Japan, clinical trials showed that there were 26 cases (4.57%) of adverse reactions in 569 cases in total, and the main adverse reactions were liver dysfunction in 16 cases (2.81%), allergy in 6 cases (1.05%), and rash in 4 cases (0.70%). Among the 569 cases, 122 (21.4%) were considered to have laboratory abnormalities, mainly including increased AST (GOT) (43/558 cases: 7.71%), increased ALT (GPT) (46/559 cases: 8.23%), and other abnormal liver function test.

In the more than 2 years since the launch of edaravone in Japan, the number of patients treated with edaravone in Japan was about 310,000, and 162 cases of acute renal insufficiency (0.052% of users) were reported, including cases with unknown causality. The occurrence time of adverse reactions showed that 155 of 162 cases had clear occurrence time, of which 85 cases occurred within 7 days after administration and reached the peak 4 days after administration. Hishida reported that among 207 patients with renal dysfunction during the administration of edaravone, 8.2% of the cases with renal insufficiency was highly related to the administration of edaravone and 34.8% of that was possibly related.

A monitoring study of adverse reactions conducted by the regulatory authority of Mitsubishi Pharmaceutical found that the incidence of edaravone related liver dysfunction was 4.3% and edaravone was considered a possible cause of renal dysfunction. Fulminant hepatitis was a serious drug-related adverse reaction of edaravone recently observed. According to statistics, 7 cases of fulminant hepatitis reported in Japan from April 2003 to February 2007 were related to the clinical application of edaravone.

According to all serious adverse reactions reported, although some serious adverse reactions are extremely rare, edaravone still adds new safety warnings, including granulocytopenia, acute lung disease, rhabdomyolysis, and hepatitis. In the revision of the 14th edition in 2009, the instructions for edaravone injection in Japan listed 7 serious adverse reactions: 1. acute renal failure, nephrotic syndrome; 2. fulminant hepatitis, liver failure,

jaundice; 3. thrombocytopenia, granulocytopenia; 4. diffuse intravascular coagulation (DIC); 5. acute lung injury; 6. rhabdomyolysis; 7. shock. allergic reactions.

Wang Wei et al. analyzed the adverse reactions of 8,645 patients by using computers to search Pubmed, SinoMed and references of the literature obtained, and comprehensively analyzed the safety of edaravone by applying meta-analysis in randomized controlled trials of edaravone in the treatment of acute cerebral infarction. The results showed that the most common adverse reactions of edaravone were mildly increased aminotransferase and abnormal renal function, with adverse reactions (283 cases) accounting for 3.27% of the total number (8,645 cases); A total of 10 randomized controlled trials of edaravone in the treatment of acute cerebral infarction was included in the meta-analysis, and the results showed that there was no statistically significant difference in the incidence of adverse events, increased aminotransferase, and abnormal renal function between the edaravone + conventional treatment group and the conventional treatment group. It has been proved that the incidence of adverse reactions of edaravone is very low in China and its clinical application is safe.

According to the current reports of serious adverse reactions, there are also some extremely rare serious adverse reactions, including granulocytopenia, acute lung disease, rhabdomyolysis, and hepatitis. In the revision of the 14th edition in 2009, the instructions for edaravone injection in Japan listed 7 serious adverse reactions: 1. acute renal failure, nephrotic syndrome; 2. fulminant hepatitis, liver failure, jaundice; 3. thrombocytopenia, granulocytopenia; 4. diffuse intravascular coagulation (DIC); 5. acute lung injury; 6. rhabdomyolysis; 7. shock. allergic reactions.

2. Objective and Outcomes

2.1. Objective

To evaluate the efficacy and safety of Y-2 Sublingual Tablets in the treatment of acute ischemic stroke.

2.2. Outcomes

2.2.1. Primary efficacy outcomes

- The proportion of subjects with mRS score ≤ 1 on Day 90 after randomization.

2.2.2. Secondary efficacy outcomes

- mRS score on Day 90;
- The proportion of subjects with mRS score ≤ 2 on Day 9;
- Change in NIHSS score from baseline to Day 14;
- The proportion of subjects with NIHSS score of 0-1 on Day 14, Day 30, and Day 90 after randomization.

2.2.3. Safety outcomes

- Incidence of adverse events (AEs) in each group;
- Incidence of treatment-related adverse events (TEAEs) in each group;
- Changes in vital signs and laboratory data in each group before and after treatment.

2.3. Main estimated target

The main clinical concerns of this study: Based on treatment policy strategy, the efficacy of Y-2 Sublingual Tablets versus placebo in patients with acute ischemic stroke was assessed by the proportion of participants with an mRS score ≤1 at Day 90 after treatment, regardless of whether the participants had delayed or discontinued the administration, or used protocol-allowed concomitant medications (as specified in Section 5.8.1) due to adverse events.

The main estimated target consists of the following attributes:

- Population: adult patients with acute ischemic stroke. The study population in this trial is determined by the inclusion and exclusion criteria defined in Section 4;
- Treatment: Y-2 Sublingual Tablets 36 mg (containing 30 mg of edaravone and 6 mg of dexborneol) or Y-2 placebo control (containing 60 μg of dexborneol to simulate the cool taste of sublingual administration of the investigational product) BID for 14 consecutive days, 28 times in total;
- Endpoint: treatment compliance status derived from the mRS score at Day 90 after treatment (compliance: mRS score ≤ 1);
- Handling of other concomitant events:
    - o Use of concomitant medications/therapies during the treatment period but continued with investigational product treatment and follow-up after

investigator assessment; or delay, interruption of administration, or discontinuation of the treatment due to AEs, but no early withdrawal from the study; or mRS scores at required time points could only be obtained through remote visit due to the impact of COVID-19: Use of treatment policy strategy (regardless of the occurrence of such events);

  - No mRS score after initiation of the treatment for any reason; or early discontinuation of treatment or withdrawal from the study due to death; or early withdrawal from protocol-specified follow-up due to AEs: use of a composite strategy (occurrence of such events was counted as a substandard mRS score);
  - Early discontinuation of the treatment or withdrawal from the study for reasons other than those mentioned above: use of the while-on-treatment strategy (i.e., mRS compliance status was evaluated based on the mRS score closest to the withdrawal from the study).

- Population-level summary: The proportion of patients whose mRS score ≤1 in the Y-2 Sublingual Tablets treatment group and the placebo treatment group was summarized based on the mRS score at Day 90 after the treatment for 14 days in line with the established treatment protocol, and the rate difference between the two groups was calculated.

3. Study design

3.1. General design

It is designed as a multicenter, randomized, double-blind, parallel, and placebo-controlled trial.

The trial is divided into three phases: screening/baseline period, treatment period, and follow-up period.

Screening/baseline period: The subjects enter the screening/baseline period after signing the informed consent form.

Prior to random grouping, at least the information and examinations involved in the inclusion and exclusion criteria should be completed and confirmed by the authorized

investigator. The rest of the baseline tests only require the collection of test samples prior to the first dose.

If the subject has completed the examinations required by the protocol within 24 hours after this onset and admission, and investigators consider it sufficient to support the diagnosis of the subject's condition, the examination may not be conducted again, as repeated examinations in a short time may impact the health of the subject or seriously affect the treatment time of the subject. The subject can directly reimburse the corresponding expenses to the Sponsor with the examination bills and details, and the specific expenses will be reimbursed according to the policies of the site.

Treatment period: Eligible subjects are randomly assigned to receive Y-2 Sublingual Tablets or Y-2 placebo for 14 consecutive days, during which the relevant tests as required by the protocol are performed and safety is assessed.

Follow-up period: Subjects after the treatment are followed up until Day 90 of treatment. Stroke Scale is used for scoring on Day 14, Day 30, and Day 90 after the first dose of the investigational product, and adverse events are recorded during follow-up periods to further assess safety.

The detailed study process and observation items are shown in the flow chart.

3.2. Sample size

It is preliminarily planned to enroll 914 subjects in this trial, Y-2 Sublingual Tablets group: Y-2 placebo control group = 1:1, Y-2 Sublingual Tablets group: 457 subjects, and placebo control group: 457 subjects. An interim analysis is scheduled to be performed after approximately 50% of subjects complete the visit on Day 90. The purpose of interim analysis is to reestimate the sample size based on primary efficacy indexes. It is conducted by the Independent Data Monitoring Committee (IDMC) and whether the sample size should be increased or remain unchanged is recommended according to the regulations of the IDMC. The sample size may be increased to 1.5 times the scheduled sample size at most, and won't be reduced.

3.3. Scientific basis for trial design

Since the 20th century, except edaravone, almost all neuroprotective agents targeting a single target or a single pathway have been clinically proven ineffective[4]. Studies have confirmed that acute ischemia leads to abnormal cellular energy metabolism and a series of ischemic cascade responses. In the very early stage after cerebral ischemia, local neuronal protein synthesis stops, membrane ion transport stops, neuronal depolarization occurs, and calcium influx leads to the release of a large amount of excitatory amino acid-glutamate. Cellular damage increases as such release aggravates calcium influx and neuronal depolarization; A large number of calcium ions enter the cell through the damage of NMDA/AMPA receptors, metabolic glutamate receptors and voltage-dependent calcium channels, and activate proteases, lipases, various kinases, nucleases and nitric oxide (NO) synthases, resulting in dysfunction of cell stability and destruction of cytoskeleton, mitochondria and cell membrane; Subsequently, the formation of free radicals and NO synthesis exacerbate further neuronal damage; In addition, the inflammatory reaction, leukocyte adhesion and infiltration, and cytokine action accompanying reperfusion will further enhance the destruction of nerve cells after ischemia and aggravate microcirculatory disorders. In the end, the activation of apoptotic genes leads to programmed cell death, resulting in the fusion of the ischemic penumbra with necrosis. It is analyzed that the clinical failure of neuroprotective drugs is largely related to the inconsistency between the action mode of neuroprotective agents and the mechanism of ischemic brain injury. Due to the complexity of ischemic mechanism, the single action mode of a single drug cannot fully protect other mechanisms of ischemic brain injury, such as apoptosis, glial cells, inflammation and intracellular mechanisms; Early and multipathway (cocktail) therapy may be a reasonable direction for neuroprotective therapy for the cascade reaction mechanism of the ischemic injury.For example, experiments with a combination use of excitatory amino acid antagonists and free radical scavengers have shown to be more effective than their respective use. Therefore, the development of multi-target or multi-pathway compound preparations is a hot trend in the international research on effective treatment of stroke.

Ischemic cerebrovascular diseases are acute and need to be relieved quickly, so injection

is the first choice for emergency treatment. However, intramuscular injection or intravenous injection can cause pain and irritation at the injection site, so it requires professional medical personnel to operate and inject products. Due to certain medical restrictions in application, it is difficult to apply to patients with out-of-hospital onset. Therefore, there is also an urgent clinical need for effective neuroprotective agents that are administered in different non-injectable modes to benefit more patients. Sublingual preparations are absorbed directly from the sublingual mucosa. The sublingual mucosa has a large surface area and strong osmotic capacity. In addition, there are numerous capillaries under the mucosa that are pooled into the internal jugular vein and directly enter the blood circulation through the superior vena cava. Therefore, drugs can be quickly absorbed after administration, with rapid onset of action, accurate quantification, and convenient use, thus avoiding the first pass effect of oral drugs. Compared with injections, sublingual preparations, especially sublingual tablets, can greatly improve the convenience of medication and the compliance of clinical patients for cerebrovascular diseases. At the same time, many stroke patients have dysphagia, so sublingual tablets are more advantageous than oral preparations.

The main components of Y-2 Sublingual Tablets are edaravone (30mg) and dexborneol (6mg). Edaravone injection, as a neuroprotective agent, is one of the drugs recommended in the Chinese Guidelines for Diagnosis and Treatment of Acute Ischemic Stroke [5] for the treatment of acute ischemic cerebral infarction. Its clinical dose is 30 mg. The phase I clinical PK study of Y-2 Sublingual Tablets has demonstrated that the exposure of edaravone in human body after the administration of 1 tablet of Y-2 Sublingual Tablets is similar to that of intravenous drip of edaravone 30 mg solution, so the recommended dose of Y-2 Sublingual Tablets is 1 tablet.

3.4. Hospitalization time

The trial has no mandatory requirement for the hospitalization time of subjects. The hospitalization time is subject to the treatment time required for the condition of subjects during the baseline period.

3.5. Definition of end of study

- Determine the final sample size according to the interim results, complete the

enrollment and visit of all sample sizes, and end the trial;

- The Sponsor and investigator jointly decide to discontinue the trial based on the risk and benefit of patients.

4. Population

4.1. Inclusion criteria

Those who meet all the following requirements:

- Age ≥ 18 years old and ≤ 80 years old, regardless of gender;
- After the onset of the disease, the National Institutes of Stroke Scale score: 6 ≤ NIHSS ≤ 20, and the sum of the fifth upper limb score and the sixth lower limb score was ≥2;
- The onset time is within 48 hours (including 48 hours);
- Patients diagnosed as ischemic stroke according to "key points for diagnosis of all kinds of major cerebrovascular diseases in China 2019", with good prognosis after the first attack or the last attack (MRS score ≤ 1 before this attack);
- The informed consent approved by the ethics committee was voluntarily signed by the patient or his legal representative.

4.2. Exclusion criteria

Those who meet any of the following items:

- Intracranial hemorrhagic diseases seen in head imaging: hemorrhagic stroke, epidural hematoma, intracranial hematoma, intraventricular hemorrhage, subarachnoid hemorrhage, etc; If it is only oozing blood, the researcher can judge whether it is suitable for inclusion;
- Severe disturbance of consciousness: the item score of La consciousness level of NIHSS was more than 1;
- Transient ischemic attack (TIA);
- Systolic blood pressure was still higher than 220mmhg or diastolic blood pressure was higher than 120mmhg after blood pressure control;
- Patients with severe mental disorders and dementia;

- Severe active liver diseases have been diagnosed, such as acute hepatitis, chronic active hepatitis, cirrhosis, etc; Or ALT or AST > 2.0 × ULN;
- Severe active kidney disease and renal insufficiency have been diagnosed; Or serum creatinine > 1.5 × ULN;
- After the onset of the disease, the drugs with neuroprotective effect in the manual have been used;
- Embolectomy or interventional therapy has been used or planned after the onset of the disease;
- Complicated with malignant tumor or undergoing anti-tumor treatment; For the subjects diagnosed with malignant tumor after enrollment, whether to continue to participate in the study can be judged by the researcher and the willingness of the subjects;
- Suffering from severe systemic diseases, the estimated survival time is less than 90 days;
- Allergic to d-borneol or edaravone or excipients;
- Patients during pregnancy, lactation, and planned pregnancy;
- Major operation history within 4 weeks before enrollment;
- Have participated in other clinical studies or are participating in other clinical studies within 30 days before randomization;
- The researcher thinks that it is not suitable to participate in this clinical study.

If a subject is found to have violated the inclusion criteria or met the exclusion criteria after enrollment, investigators may judge whether the subject is suitable to continue the study based on the risks and benefits.

4.3. Definition of onset time

The onset time of stroke is defined as the time when the patient's symptoms start. If the onset occurs during sleep, the onset time is the time when the patient last acted normal.

The 48 hours specified in the protocol are the time from onset to the first administration of the investigational product.

5. Study treatment

5.1. Study drug

Investigational product

Y-2 Sublingual Tablets, each contains 30 mg of edaravone and 6 mg of dexborneol. Batch No.: Q0120006; Expiry date: June 16, 2022.

Control product

Placebo control was used in this study.

Y-2 is a Class I new drug, and there is no similar drug recognized and validated to be effective, so placebo is used as the control.

Y-2 placebo contains 60 μg of dexborneol to simulate the cool taste of sublingual administration of Y-2 Sublingual Tablets. This dose is verified to have no efficacy by animal experiments, and its composition of excipients is consistent with that of the Y-2 Sublingual Tablets. Batch No.: Q0120001; Expiry date: June 13, 2022.

Multiple batches of drugs may need to be prepared in this trial study, and relevant materials will be submitted for filing before the adoption of new drugs.

Storage condition

This product should be stored in a sealed and cool place (not more than 20°C), with a tentative shelf life of 2 years. In the influencing factor test, it is stable at a high temperature of 40°C with packaging for 1 month. Therefore, the drug needs to be stored in a cool place (not more than 20°C) for long-term storage, and can be exposed to the environment not more than 40°C for a short time (1 month).

5.2. Management of study drug

5.2.1. Packaging and labeling of study drug

The investigational product and the control product are identical in color and shape. In order to keep it blind, the packaging of the two groups of drugs are identical, the package batch number is uniformly marked, and the shortest shelf life of the two drugs is selected as the shelf life.

Each randomized subject will be assigned a large box of drugs with the corresponding drug number, which contains 30 small boxes (including 2 small boxes of backup drugs); each small box contains 1 tablet of drug for one use.

The specific drug filling specifications and quantity are shown inTable5.2- 1:

Table 5.2- 1 Drug Filling Specification

**Group**	**Number of large boxes**	**Number of small boxes**	**Drug in each small box Composition**
Treatment group	1	30	1 tablet
Control group	1	30	1 tablet

The contents of the package label of this clinical investigational product are as follows:

Label of large box package

Clinical investigational product of Y-2 Sublingual Tablets for the treatment of acute ischemic stroke

Drug No.: _____________

This product is for clinical trials only

[Packaging] A total of 30 small boxes with 1 tablet in each small box.

[Strength] 30 mg of edaravone and 6 mg of dexborneol; or 60 μg of dexborneol

[Administration and dosage] Take 1 tablet of the drug sublingually each time. Place the drug at the edge of the lingual frenulum, and keep it until there is no obvious foreign body sensation. Avoid chewing or swallowing undisintegrated drugs directly during administration.

[Storage] Sealed and stored in a cool place (not more than 20°C)

[Manufacturer] 2Y-Biopharma Ltd.

[Product batch No.] Q0120001\Q0120006

[Expiration date] June 13, 2022<br>[Note] **The remaining unused drugs should be recycled to the Sponsor and kept away from children.**

Label of small box package

**Clinical investigational product of Y-2 Sublingual Tablets for the treatment of acute ischemic stroke**<br>**Drug No.: _____________**<br>**This product is for clinical trials only**<br>[Packaging] 1 tablet in each box.<br>[Strength] 30 mg of edaravone and 6 mg of dexborneol; or 60 μg of dexborneol<br>[Storage] Sealed and stored in a cool place (not more than 20°C)<br>[Manufacturer] 2Y-Biopharma Ltd.<br>[Product batch No.] Q0120001\Q0120006<br>[Expiration date] June 13, 2022<br>[Note] **The remaining unused drugs should be recycled to the Sponsor and kept away from children.**

Note: To keep it blind, the product batch number on the label includes the batch number of the investigational product and the control product, and the shortest shelf life of the two drugs is selected as the shelf life.

5.2.2. Receipt and storage of study drug

All study drugs in this study are supplied by the Sponsor in a unified manner. the sites

should assign special personnel to receive and store the study drugs. It is the responsibility of the site to receive and store the study drugs in accordance with applicable regulatory requirements and the Sponsor's designated requirements.

All study drugs should be stored in a safe area until dispensed to the subjects, and are accessible only to those involved in the clinical study. The study drug management personnel should fully supervise the storage environment, and keep relevant temperature logs as source documents. All study drugs must be used in this study and cannot be used for other purposes.

The investigators should ensure that all study drugs are used as specified in this protocol.

5.2.3. Distribution, recovery, and destruction of study drug

In order to strictly manage and use the study drugs, the sites should have dedicated personnel responsible for the management of study drugs, including distribution, recovery, and destruction, and ensuring that all documents related to the study drugs generated during the study are recorded in a timely and accurate manner.

Each site should assign special personnel to manage and distribute study drugs. Drug management personnel should distribute the study drugs to subjects according to the requirements of the clinical trial protocol, and timely record the distribution and recovery of each study drug in a special record sheet.

Study drugs may not be used for any purpose other than that described in the clinical trial protocol. Investigators should ensure that all investigational drugs are only used for subjects involved in the clinical trial with the administration and dosage specified in the clinical trial protocol. Study drugs may not be transferred to any non-clinical trial subjects.

Monitors are responsible for overseeing the management process of study drugs.

All unused study drugs should be recovered and destroyed by the Sponsor. The Sponsor will provide destruction records.

5.3. Randomization and blinding

It is designed as a multicenter, randomized, double-blind, parallel, and placebo-controlled trial. Subjects are randomized in a 1:1 ratio. Random number table is generated by simulation using standardized software.

The trial adopts a randomized method of centralized competitive enrollment. Each subject will be assigned a "screening number" after signing the informed consent form and before conducting study-related examinations. The screening numbers of subjects should not be reused.

After qualified subjects are selected after the screening, investigators or their designated personnel obtain the randomization number and corresponding drug number through the randomization system, and distribute corresponding study drugs according to the randomization number and drug number.

Stratified randomization factors include:

Onset time: ≤24 hours VS >24 hours;

Sites.

The color, description, and packaging (including batch number and expiration date) of the investigational product (Y-2 Sublingual Tablets) and placebo control (Y-2 placebo) are the same. The blinding process is completed by statisticians unrelated to this clinical trial. After the blinding, blind codes will be sealed in duplicate and stored in the leading unit and the Sponsor respectively.

5.4. Unblinding

5.4.1. Common unblinding regulations

The one-time unblinding method is adopted in this trial. After blind verification, the data will be locked and unblinded, and the corresponding group of each subject will be defined.

For interim analysis, the unblinded data will be centrally reviewed by IDMC members according to IDMC regulations. Statistical analysis of the data will be completed by third-party unblinded statisticians independent of the Sponsor, so as to keep blinding during the trial.

5.4.2. Emergency unblinding

Emergency unblinding is allowed only in the following circumstances: in case of a serious adverse event suspected to be related to the investigational product, the principal investigator of the site should report to the Sponsor to decide whether to initiate the emergency unblinding procedure. Once the emergency unblinding is conducted, the case is

considered dropped out. If the emergency unblinding happens during the treatment period, the medication should be stopped.

This double-blind trial will be invalid if all blind codes are disclosed or the emergency unblinding rate exceeds 20% during the study.

5.5. Treatment protocol

Take 1 tablet of the drug sublingually each time. Place the drug at the edge of the lingual frenulum, and keep it until there is no obvious foreign body sensation. Avoid chewing or swallowing undisintegrated drugs directly during administration.

The first dose should be administered as soon as possible after randomization; According to the results of human pharmacokinetic study, the plasma concentration has decreased to 1/10 of Cmax 6 hours after administration. The second dose should be not less than 6 hours but not more than 12±1 hours from the first dose. The drug should not be administered more than 2 times a natural day; Each subsequent dose should be taken for 14 consecutive days (28 doses) at an interval of 12 ± 1 h (calculated with the fixed administration time as the baseline point).

Each subject should only take up to 28 doses. If the medication is missed or suspended halfway, the subject should continue to take the drug after 14 days, but not more than 28 doses or 14+3 days. Backup drugs should only be used in the following cases, otherwise they should be uniformly recovered to the Sponsor.

1) Loss of drugs;
2) Breakage of drugs.

If all 30 tablets are taken, it is inferred from the available toxicological evidence that there will be no significant risks in theory, but such incidents still need to be truthfully recorded.

5.6. Treatment adjustment

No gastrointestinal safety problem was observed during the animal test of Y-2 Sublingual Tablets. Even if the drug is taken by mistake, it will not cause serious consequences, but may weaken the drug effect. Therefore, in the case of medication by mistake, the investigator should still be informed, and this medication will not be repeated.

5.7. Criteria for discontinuation of treatment or withdrawal

This Section describes the circumstances under which a subject should be withdrawn from the study prematurely at the discretion of the investigator or the Sponsor. A distinction should be made between the concepts of discontinuation of treatment and withdrawal from the entire study. The procedures (including follow-up) to be performed and the data to be collected in different situations should also be defined. If resumption of treatment is allowed after drug suspension, relevant criteria should be clearly defined. If it is allowed to replace the dropout subjects, the relevant criteria should be clearly described.

5.7.1. Criteria for discontinuation of or withdrawal from the treatment

Treatment should be discontinued in the following cases:

1. Intolerable adverse reactions occur during treatment that the investigator concludes that further treatment is not appropriate;
2. Serious violation of the inclusion and exclusion criteria is found after enrollment, and both the investigator and the Sponsor think the treatment needs to be discontinued;
3. New disease changes unrelated to the investigational product occur during treatment that the investigator concludes that further treatment is not appropriate;
4. Interventional therapy (including cerebrovascular stenting) is considered necessary by the investigator during treatment;
5. The subject is unwilling to continue the treatment.

All subjects who have completed the informed consent form and are eligible for enrollment in the trial have the right to withdraw from the clinical trial at any time. The subjects can withdraw from the study if the following conditions are met during the study:

1) The subject experiences intolerable adverse reactions;
2) The subject asks to withdraw and is unwilling to continue to participate in the study;
3) The investigator thinks that the subject cannot complete this study due to poor compliance;
4) The subject is lost to follow-up;
5) The subject is pregnant;

6) The subject participates in other clinical trials;
7) The investigator considers that the subject is not appropriate to continue to participate in the study for other reasons.

For withdrawn subjects, the original adverse events will still be followed up, but no other information will be collected, including new adverse events, unless the investigator thinks such events are related to the drug.

5.7.2. Handling of discontinuation of or withdrawal from the treatment

Subjects who have withdrawn during the trial are all considered as dropout cases, and the reasons are shown in Section 5.7.1 "Reasons for withdrawal".

When a subject drops out, the investigator should contact the subject as much as possible to complete the assessment items that can be completed at visit 4 (withdrawal visit). If the subject drops out during the treatment period, the time of the last medication should be recorded. Those who drop out due to adverse reactions also need to be treated as specified in Section 7.3.

5.7.3. Criteria for early discontinuation

The study may be early discontinued at any time with the consent of the investigator and Sponsor, in the best interests of the subject and for reasonable medical or ethical reasons. During the discontinuation of the study, the Sponsor, the contract study organization, and the investigators will ensure that the interests of the subjects are fully considered.

The following is the criteria for early discontinuation of study:

1. The drug regulatory authority, the Ethics Committee, the Sponsor, or investigators believe that the study drug has major safety risks;
2. Major defects are found in the study protocol, or major deviations or human errors are found during the implementation of the study, which seriously affect the quality of the study and make it difficult to achieve the study objective;
3. The Sponsor may discontinue the study for any scientific, medical or ethical reasons, provided that the rights, safety, and health of the enrolled subjects are fully considered;
4. Other reasons that the Sponsor or the investigator consider as unsuitable for

continuing the study.

5.8. Previous/concomitant medication and therapy

5.8.1. Allowed concomitant medication/therapy

Study period refers to the period from the time of signing the informed consent form to Day 90 of medication or the last follow-up.

During the study period, therapeutic drugs excluding neuroprotective agents recommended in the *Chinese Guidelines for Diagnosis and Treatment of Acute Ischemic Stroke* 2018 and the *Chinese Guidelines for Secondary Prevention of Ischemic Stroke and Transient Ischemic Attack* 2014[6] are allowed to be used:

(I) General treatment and supportive drugs: antipyretics, dehydrating agents, hypotensors, lipid lowering, glucose lowering, nutritional support, antibiotic treatment. Mannitol is used as dehydrating agent only to reduce intracranial pressure due to its effect in free radical scavenging.

(II) Drugs for improving cerebral circulation:

(1) Antiplatelet drugs: including Aspirin, Clopidogrel, Dipyridamole;

(2) Anticoagulant drugs: including unfractionated heparin, low-molecular-weight heparin, heparinoids, oral anticoagulants (such as Warfarin) and thrombin inhibitors (such as Argatroban);

(3) Defibrinogen drug: including defibrase, batroxobin, ancrod, lumbrokinase, acutobin;

(4) Volume expansion drug: crystalloid fluids (such as glucose, sodium chloride injection, etc.), blood products (such as whole blood, plasma, albumin, etc.), artificial colloid fluids (such as gelatin, dextran, succinic acid gelatin, etc.).

(III) The subjects with underlying medical conditions that require long-term medication may continue to take it during the study period.

(IV) If the subject has adverse reactions, the investigator shall decide whether to use symptomatic drugs.

(V) Considering the treatment needs of the placebo group, it is allowed to use Shuxuetong injection and ligustrazine injection without neuroprotective mechanism for indications of cerebral infarction, and other drugs not for cerebral infarction or related

indications as mentioned in their package inserts.

(VI) Appropriate rehabilitation treatment shall be selected according to the *Chinese Guidelines for Diagnosis and Treatment of Acute Ischemic Stroke* 2018

All drugs used at the same time should be recorded and explained in detail on the CRFs.

5.8.2. Prohibited concomitant medication/therapy

The following treatment is prohibited during the study:

Concomitant medication of following drugs should be avoided during the study:

1) Drugs with indications of neuroprotective agents as mentioned in their package inserts or those with similar mechanism of action, including commercially available edaravone, edaravone dexborneol concentrated solution for injection, nimodipine, gangliosides, citicoline, piracetam, oxiracetam, butylphthalide, human urinary kallidinogenase (kallikrein), cinepazide, mouse nerve growth factor, possible neuroprotective cerebrolysin (cerebroprotein hydrolysate), deproteinised calf blood serum injection, and deproteinised calf blood extractive injection. It is also prohibited to use mannitol for the purpose of neuroprotection rather than reducing intracranial pressure.
2) Drugs with indication of cerebral infarction as mentioned in their package inserts: Breviscapine Injection, Astragali Injection, Danshen Injection, Compound Danshen Injection, Sodium Aescinate for Injection, Puerarin Injection, Angelica Injection, Compound Angelica Injection, Safflower Injection, Ginkgo Leaf Extract and Dipyridamole Injection, Extract Of Ginkgo Biloba Leaves Injection, Ciwujia Injection, Kudiezi Injection, Xingnaojing Injection, Xuesaitong Injection/Tablet/Capsule, Xueshuantong Injection, Mailuoning Injection, Compound Musk Injection, Naoxuetong Oral Liquid/Granule, Naoxuekang Tablet/Granule/Capsule/Oral Liquid, Naoluotong Capsule, Yimaikang Tablet/Capsule, Naoxintong Tablet/Capsule, Dahuoluo Pill, Xiaohuoluo Pill, Sanfeng Huoluo Pills, Huatuo Zaizao Pill, Xinnaoshutong Tablet/Capsule, Thrombolytic Capsule, Xiaoshuan Tongluo Tablet, Vinpocetine, etc.

3) It's forbidden to use unmarketed drugs or other drugs for clinical trials during the study.
4) Drugs under post-marketing investigation with neuroprotection as the main evaluation index.

The administration of all drugs during the study should be recorded.

6. Study process

Item observed \ Visit time		Screening / baseline period	Treatment period			Follow-up period	
		Visit 1<br>From onset to the first dose	The first dose	Visit 2<br>Day 7±1	Visit 3<br>Day 14+3	Visit 4<br>Day 30±7	Visit 5<br>Day 90±7
Informed consent		X					
Collection of demographics and disease characteristics		X					
Past medical history and medication history		X					
Evaluation of the inclusion/exclusion criteria		X					
Vital signs and physical examination		X		X	X	X	X
Laboratory and auxiliary	Blood routine	X		X	X	X	X
	Urine routine	X		X	X	X	X

examinations	Hepatic and renal function	X[1]		X	X	X	X
	Myocardial enzyme	X		X	X	X	X
	Electrolytes	X		X	X	X	X
	Fasting blood glucose	X[2]		X	X	X	X
	Blood lipid	X		X	X	X	X
	Coagulation function	X		X	X	X	X
	Homocysteine	X		X	X	X	X
	Electrocardiogram	X		X	X	X	X
	Pregnancy test	X[3]					X[3]
	Cranial CT	X					
	Cranial MRI	X[4]					
Score	mRS score	X[5]			X	X	X
	NIHSS score	X			X	X	X

Parallel exploratory biomarker	X		X	X		
SSS TOAST classification		X[6]				
Randomization	X					
Dosing		X[7]				
Concomitant medication	X[8]					
Adverse event assessment	X[8]					

[1] Results of ALT, AST, and serum creatinine need to be obtained prior to randomization.

[2] Non-fasting state is allowed in the tests prior to treatment, indicating whether it's fasting or not.

[3] For women of childbearing age only.

[4] Optional and completed during the study. It is not a protocol deviation when the subject is unable to complete the MRI due to his/her own reasons.

[5] mRS score before this onset.

[6] Completion after SSS TOAST classification and enrolling is acceptable.

[7] Use as soon as possible after randomization.

[8] Concomitant medications and adverse events should be recorded from the time of signing the informed consent form to Day 90 of medication or the last follow-up. If adverse event occurs, the subject will be followed up until the adverse event disappears or is restored to baseline levels or is not clinically significant.

6.1. Screening period (visit 1)

- Informed consent form
- Demographics, including age, gender, height and weight (Height and weight information of subjects with unfavorable limbs can be obtained through interrogation);
- Disease attributes;
- Past medical history and medication, disease history and medication history before enrollment;
- Vital signs and physical examination, including heart rate, blood pressure, body temperature and respiratory rate. Physical examination is evaluated by organ and system;
- Blood routine, blood biochemistry (hepatic and renal function, blood glucose (non-fasting), blood lipids, myocardial enzyme, electrolytes and homocysteine), urine routine, coagulation function and ECG examination;
- Pregnancy test (for women of childbearing age only);
- NIHSS score; (If thrombolysis subjects have completed the NIHSS for screening period before thrombolysis, the pre-thrombolysis score should be used as the basis for enrollment)
- mRS score before this onset;
- Cranial CT (plain scan);
- Cranial MRI (optional and completed during the study);
- Randomization.

6.2. Treatment period

6.2.1. D7 (±1) (visit 2)

- Vital signs and physical examination (including weight)
- Blood routine, blood biochemistry (Hepatic and renal function, blood glucose (fasting), blood lipids, myocardial enzyme, electrolytes and homocysteine), urine routine, coagulation function and ECG examination

6.2.2. D14 (+3) (visit 3)

- Vital signs and physical examination (including weight)
- Blood routine, blood biochemistry (Hepatic and renal function, blood glucose (fasting), blood lipids, myocardial enzyme, electrolytes and homocysteine), urine routine, coagulation function and ECG examination
- NIHSS score
- mRS score

Note: If the last medication is on Day 15, visit 3 shall be on D15+3

6.3. Follow-up period

6.3.1. D30 (±7) (visit 4)

- Vital signs and physical examination (including weight)
- Blood routine, blood biochemistry (Hepatic and renal function, blood glucose (fasting), blood lipids, myocardial enzyme, electrolytes and homocysteine), urine routine, coagulation function and ECG examination
- NIHSS score
- mRS score

6.3.2. D90 (±7) (visit 5)

- Vital signs and physical examination (including weight)
- Blood routine, blood biochemistry (Hepatic and renal function, blood glucose (fasting), blood lipids, myocardial enzyme, electrolytes and homocysteine), urine routine, coagulation function and ECG examination
- NIHSS score
- mRS score
- SSS TOAST classification (to be completed during the trail period after enrollment)

Note: For subjects who drop out in the midway, they should try to complete the visit (withdrawal visit) according to the requirements of visit 5.

At sites participating in the research of exploratory indicator biomarkers, a tube of venous blood (about 4mL) should be sampled from the subject before the first administration and on Day 7 (± 1) (and Day 14 (+3)) after the administration, and send it to the unified

laboratory for testing. See Section 2.2.4 for specific indicators.

6.3.3. Follow-up endpoint

All subjects who have been treated with investigational product need to be followed up until:

1. Visit 5 has been completed according to the proposal;
2. Subjects died during follow-up period;
3. Serious violation of the inclusion and exclusion criteria is found after enrollment, and both the investigator and the Sponsor think the follow-up visit needs to be discontinued;
4. If the subject withdrew from the study midway (see Section 5.7.1 for Reasons for withdrawal), the time of the last evaluation shall be the follow-up endpoint. If the subject withdrew due to intolerable adverse reactions, the follow-up visit shall be carried out according to Section 7.3.

7. Study measures

7.1. Efficacy measures

- For the overall outcome scale, it is assessed by the Modified Rankin Scale (mRS), which measures the global disability. See Appendix 2 for details.
- Neurological Disability Score (NDS) scale

  See Appendix 3 for the NIH Stroke Scale (NIHSS).

7.2. Safety measures

7.2.1. Safety evaluation indexes

- Vital signs and physical examination, including heart rate, blood pressure, body temperature and respiratory rate. Physical examination is evaluated by organ and system;
- Laboratory test, including blood routine, urine routine (qualitative or quantitative), blood biochemistry (including hepatic and renal function, blood glucose, blood lipids, myocardial enzyme, electrolyte), coagulation function, electrocardiogram, pregnancy test (women of childbearing age). See Table 7.2- 1 for detailed test items

Table 7.2- 1: Laboratory test items and observation indicators

Laboratory item	Requirement and observation indicator

<table>
<tr><td colspan="2">Blood routine</td><td>White blood cell count, neutrophil count, lymphocyte count, hemoglobin, platelet</td></tr>
<tr><td rowspan="6">Blood biochemistry</td><td>Hepatic and renal function</td><td>Alanine transaminase (ALT), aspartate aminotransferase (AST), alkaline phosphatase (ALP), TBil, DBil, creatinine, urea, total protein, albumin</td></tr>
<tr><td>Blood glucose</td><td>Determination of serum glucose</td></tr>
<tr><td>Blood lipid</td><td>Total cholesterol, triglyceride</td></tr>
<tr><td>Myocardial enzyme</td><td>Creatine Kinase (CK), CK-MB, Lactate dehydrogenase (LDH)</td></tr>
<tr><td>Electrolytes</td><td>Serum potassium, serum sodium and serum chlorine</td></tr>
<tr><td>Others</td><td>Homocysteine</td></tr>
<tr><td colspan="2">Coagulation function</td><td>Prothrombin time (PT), Activated partial thromboplastin time (APTT), International normalized ratio (INR), Fibrinogen (FIB)</td></tr>
<tr><td colspan="2">Urine routine</td><td>Leukocyte, protein, glucose, ketone bodies, occult blood</td></tr>
<tr><td colspan="2">Electrocardiogram</td><td>Standard 12-lead ECG or above</td></tr>
<tr><td colspan="2">Pregnancy test</td><td>Blood or urine HCG pregnancy test</td></tr>
</table>

7.2.2. Laboratory test and evaluation on safety

This study received laboratory data from various sites.

The specific laboratory parameters included in each set of laboratory test (for example, hematology, biochemical tests and urine analysis) are determined by the site and implemented according to the corresponding SOP.

- The investigator must review the laboratory report, record the evaluation and record any clinically relevant changes during the study in the AE section of the case report form (CRF). The laboratory report must be filed with the source file.
- If a non-protocol laboratory evaluation conducted in a local laboratory of the institution results in a change of the subject management procedures or such change

is deemed clinically significant by the investigator (for example, SAE or AE or dose change), the results must be recorded in the corresponding CRF.

- Any laboratory abnormality with clinical significance should be repeatedly evaluated as much as possible until all serious adverse events on test value, and adverse events related to the study or making the subject stop the study treatment are followed up until the event is cured (including returning to the baseline value), and the subject's status changes to long-term stability, the subject is lost to follow-up or died.

7.3. Safety report and evaluation

During the study, the investigator and his/her designated qualified personnel are responsible for detecting and recording all events observed during the study that meet the definition of adverse events or serious adverse events according to the standards and definitions specified in the protocol. The investigator should evaluate the adverse events, monitor the safety status of subjects and take corresponding protective measures to ensure the safety of subjects, and report according to relevant requirements.

7.3.1. Definition of adverse event, serious adverse event and suspected unexpected serious adverse event

Adverse Event (AE) refers to all adverse medical events that occur after the subject receives the investigational product, but not necessarily have causal relationship with the investigational product. It can be manifested as symptoms, signs, diseases or laboratory abnormalities. Therefore, AEs may be any adverse, unintentional physical sign (including abnormal laboratory results), symptom or disease (new or worsening) that is chronologically associated with the use of a certain study drug.

Events that meet the definition of adverse events include:

- Abnormal laboratory test results (hematology, clinical biochemistry or urine analysis) or other safety evaluations (such as ECG, radiological scan, vital sign measurement), including those that are deteriorated relatively to the baseline and are considered clinically significant according to the medical and scientific judgment of the investigator (that is, it's not related to the progress of underlying diseases).

- The deterioration of preexisting diseases, including the increase in the frequency and/or severity of the diseases.
- New symptoms detected or diagnosed after starting the administration of investigational product (even if they may already exist before the study starts).
- Signs, symptoms or clinical sequelae of suspected drug - drug interaction.

Events that do not meet the definition of adverse events include:

- Abnormal laboratory results or other abnormal safety evaluations related to underlying diseases. However, if the investigator believes that the severity is beyond the expected level based on the condition of the subject, it should be considered as an AE.
- The disease under study, or the expected progress, signs or symptoms of the disease under study. However, if the severity is beyond the expected level based on the subject's condition, it shall be considered as an AE.
- Medical or surgical operations (such as endoscopy, appendectomy). Please note that the disease causing such operation is an AE.
- There are no adverse events (hospitalization for social reasons and/or for convenience).
- The underlying disease or condition existing or detected at the beginning of the study is not deteriorated obviously with daily fluctuation within the expected range.
- Existing diseases or signs and/or symptoms not related to the study before the first trial medication. These events should be recorded in the medical history section of eCRFs.

Definition of serious adverse event

Serious Adverse Event (SAE) refers to an adverse medical occurrence in subjects, such as death, life threatening, permanent or serious disability or loss of function, hospitalization required or prolonged hospitalization, congenital abnormalities or birth defects, etc., after receiving the investigational product.

- Result in death
- Life-threatening

  It means that the subject is already in danger of death when the AE occurs, and it does not mean that the AE may cause death if it becomes more severe.
- Permanent or serious disability or loss of function

AE results may cause serious inconvenience or interference to the normal life and activities of subjects.

- Hospitalization required or hospitalization extended

  Due to an AE, the subject has to be hospitalized or his/her hospitalization is prolonged regardless he/she was already to be discharged; It is necessary to clarify that this condition is caused by adverse events, rather than elective operation, non-medical reasons, etc.

- Congenital abnormality or birth defect

  The offspring of the subjects suffer from malformations or congenital functional defects.

- Other significant medical events

  Medical and scientific judgment is required for deciding whether to accelerate the report of other situations. Significant medical events may not immediately endanger his/her life, cause death or hospitalization, but they are usually considered as a serious event if medical measures are needed to prevent the occurrence of one of the above situations. For example, important treatment in the emergency room or allergic bronchospasm at home, cachexia or convulsion without hospitalization, drug dependence or addiction, etc.

When it fails to clearly judge whether it is a SAE, the investigator should consult with the Sponsor and the Ethics Committee**.**

Definition of suspected unexpected serious adverse event (SUSAR)

Suspected Unexpected Serious Adverse Event (SUSAR) refers to the suspected and unexpected serious adverse events whose nature and severity of clinical manifestations are beyond the existing information such as the investigator's brochure of investigational product, the instructions of marketed drugs or the summary of product characteristics.

7.3.2. Time period and frequency of collecting information related to adverse events and serious adverse events

AE collection: This study should start from the signing of the informed consent form by the subject to the time point specified in the study flow diagram. The medical events not related to the study intervention that occur after the signing of the informed consent form and

before the first medication should be recorded in the CRF as medical history/concomitant disease, rather than a part of AEs.

SAE collection: This study should start from the signing of the informed consent form by the subject to the time point specified in the study flow diagram.

The investigator does not have to actively collect AEs or SAEs after the safety follow-up period. However, if the investigator learns of any SAE (including death) at any time after the subject leaves the study and believes that such event is reasonably related to the study intervention or participation in the study, the investigator must collect and report it in a timely manner.

7.3.3. Methods for collecting adverse events and serious adverse events

Please don't introduce bias when detecting AEs and/or SAEs. Open-ended and non-guided oral questioning of subjects is the preferred method to inquire about the occurrence of AEs, for example:

- "How do you feel?"
- "Has your health improved or deteriorated since the last visit?"
- "Since the last visit, have you taken any new drug? Have you stopped or changed any pharmacotherapy you are using?"

7.3.4. Follow-up visit of adverse events and serious adverse events

After the initial AE/SAE report, the investigator needs to follow up each subject at the follow-up visit/contact to obtain more information. For all SAEs, and AEs related to the study or causing the subject to stop the study treatment should be followed up until the event is cured (including returning to the baseline value), the subject's status changes to long-term stability, the subject is lost to follow-up or died, or the investigator believes that it's not necessary to continue the follow-up visits or provides other reasonable explanations. The investigator is responsible for conducting or arranging follow-up visits with medical needs or as required by the Sponsor to fully clarify the nature and causal relationship of AEs or SAEs.

When reporting information of follow-up visits, the investigator needs to update the new information with the original electronic report form, and then print the paper report form and

re-sign the name and date. The time limit and process of report of SAE follow-up visits are the same as the initial report.

7.3.5. Record and evaluation of adverse events and serious adverse events

Record of adverse events and serious adverse events

The investigator is responsible for reviewing all documents related to the event (such as hospital course records, laboratory tests and diagnostic reports) and recording all information related to AEs/SAEs in the case report form. The investigator should try to determine the diagnosis of each event based on physical signs, symptoms and/or other clinical information. If possible, the diagnosis (rather than individual signs/symptoms) should be recorded as an AE/SAE. If the diagnosis is unclear, the named of AE can be temporarily reported by symptoms, signs and abnormal examination, and each symptom, sign and examination shall be recorded separately and updated correspondingly later when the diagnosis is clear.

The investigator, in principle, should not send the copy of medical records of the subject to Jiangsu Simcere Pharmaceutical Group. In some cases, Jiangsu Simcere Pharmaceutical Group may request to provide the copy of medical records of some cases. In this case, the identification information (except the subject code) of all the subjects should be blocked (blackening or other measures) before the copy of the medical record is submitted to Jiangsu Simcere Pharmaceutical Group.

During the collection and evaluation of AEs and SAEs, the name, starting time, ending time or outcome, severity, seriousness, concomitant disease, concomitant medication, event description, causal relationship evaluation, etc. of an AE should be recorded.

Severity of adverse events and serious adverse events

The severity of all AEs is evaluated using the NCI Common Terminology Criteria for Adverse Events (CTCAE) Version 5.0 in this study. For AEs not defined in the NCI-CTCAE, the following rules will be referred to determine the severity:

Grade 1	Mild; asymptomatic or mild symptoms; clinical or diagnostic observations only; intervention not indicated.

Grade 2	Moderate; minimal, local or noninvasive intervention indicated; limiting age-appropriate instrumental activities of daily living, such as preparing meals, shopping for groceries or clothes, using the telephone, managing money, etc.
Grade 3	Severe or medically significant but not immediately life-threatening; hospitalization or prolongation of hospitalization indicated; disabling; limiting self care ADL (referring to bathing, dressing and undressing, feeding self, using the toilet, taking medications, and not bedridden).
Grade 4	Life-threatening consequences; urgent medical intervention indicated.
Grade 5	Death related to an adverse event

Severity is the category used to measure the intensity of an event, and both AEs and SAEs can be evaluated using NCI-CTCAE.

Causality assessment of adverse events and serious adverse events

Causality assessment is one of the criteria used to determine regulatory reporting requirements. The investigator is responsible for assessing the relationship between the study treatment and the occurrence of each AE/SAE. To determine whether the AE is causally related to the drug, the investigator will consult the investigator's brochure or information on the marketed product and consider the following aspects:

- Is there a reasonable temporal relationship between the occurrence of AE and the investigational product? What is the time interval between the onset of AE and the first and last doses?
- Can the symptoms and signs be caused by the mechanism of action of the drug itself or the action of metabolic components?
- Are symptoms/signs relieved or resolved in the absence of other treatments for the AE after dose reduction or discontinuation?
- Do the symptoms/signs reappear or worsen after re-medication?

- Can it be explained by the subject's concomitant diseases, concomitant medications, or other reasons?
- Has similar situation been reported in domestic and foreign literature?

Furthermore, the five-grade method (definitely related, probably related, possibly related, unlikely/remotely related, definitely not related) is used to assess the causality between the reported AE and the investigational product.

The investigator must document and review each AE/SAE, provide causality assessment (although in some cases the investigator may have limited information on the SAE) and update the causality assessment based on follow-up information.

In the assessment of whether the SAE report complies with the standard of accelerated reporting of SUSAR, it is classified as "unlikely/remotely related" in the two-grade method if the causality is assessed to be "not related".

7.3.6. Requirements for regulatory reporting of serious adverse events

Upon becoming aware of any SAE, whether or not it is related to the investigational product or listed in the investigator's brochure, the investigator should report in accordance with regulatory requirements to meet legal obligations and ethical responsibilities for the safety of subjects and the safety of clinical studies. The investigator should keep the written documents of all the above reports to show that each event received has been properly reported.

The Sponsor will comply with country-specific regulatory requirements for safety reporting to regulatory authorities, the Institutional Review Boards (IRB) / the Ethics Committee (IEC), clinical trial institutions and investigators, and notify local and other regulatory authorities of safety information about the investigational product in accordance with requirements and the Sponsor's policies. The investigator will review the safety reports from the Sponsor that describe SUSAR or other safety-specific information, then archive them with the investigator's brochure and notify the Ethics Committee.

The Sponsor will promptly investigate the SAEs occurred with the investigator, and take necessary actions to ensure the safety and rights of the subjects.

7.3.7. Serious adverse event reporting to the Sponsor

The investigator or designee must complete the rapid SAE reporting within 24 hours of being informed of the occurrence of SAE in the subject, which should include as detailed and useful content as possible. Even if not all the information of the SAE is available at that time, it still needs to be reported within 24 hours. The SAE report should be updated by the investigator within 24 hours after additional relevant information is available.

The investigator or designee will fill in the relevant information using the Jiangsu *Simcere Serious Adverse Event (SAE) Report Form*, print and then sign the date and name, and send the scanned copy to the Sponsor by email (safety@simcere.com) within 24 hours.

7.3.8. Pregnancy

All subjects should use reliable contraceptives for 30 days from signing the informed consent form to the last dose to avoid pregnancy of themselves or their female partners. If any pregnancy (including pregnancy confirmed after 90 days) occurs in the subject (or male subject's female partner) within 90 days from the time when the subject receives at least one dose of the investigational product to the last dose, the investigator should examine the safety of the subject and the fetus and take appropriate actions upon discovery; record the pregnancy information in the pregnancy report form and report to the Sponsor within 24 hours after obtaining the information.

All pregnancies should be tracked to outcome until the infant is full term (usually 6 to 8 weeks after the expected date of delivery) or early termination of pregnancy (including spontaneous or selective abortion).

Although pregnancy is not considered an AE or SAE, pregnancy complication or selective termination of pregnancy should be reported as an AE/SAE. Abnormal pregnancy outcomes (e.g., spontaneous abortion, fetal death, stillbirth, congenital anomalies, ectopic pregnancy) are considered SAEs and reported as SAEs (*Simcere Pregnancy Report Form* and *Simcere Serious Adverse Event (SAE) Report Form* are required to be completed at the same time).

In principle, any female subject who becomes pregnant during the study period should immediately discontinue the investigational product.

8. Statistical analysis

8.1. Determination of sample size

The objective of this study is to evaluate the efficacy and safety of Y-2 Sublingual Tablets in the treatment of acute ischemic stroke. The primary efficacy index is the proportion of subjects with mRS score ≤ 1 on Day 90 of treatment. The sample size is calculated based on the primary efficacy index. According to the previous clinical studies[7-12], it is expected that the proportion of subjects with mRS score ≤ 1 on Day 90 of treatment is 50% in the test group and 40% in the control group. Taking bilateral α as 0.05, the power as 80% and the random ratio as 1:1, the sample size for each group is calculated to be 388. Considering the 15% dropout rate, then it is planned to enroll 457 cases in each group, with a total sample size of 914 cases.

An interim analysis is scheduled to be performed after approximately 50% of subjects complete the visit on Day 90. The purpose of interim analysis is to reestimate the sample size based on primary efficacy indexes. It is conducted by the Independent Data Monitoring Committee (IDMC) and whether the sample size should be increased or remain unchanged is recommended according to the regulations of the IDMC. The sample size may be increased to 1.5 times the scheduled sample size at most, and won't be reduced.

8.2. Analysis sets

Full Analysis Set (FAS): According to the basic principles of intention-to-treatment (ITT), all randomized subjects are included in the full analysis set. The primary efficacy evaluation of this study is based on the full analysis set.

Per Protocol Set (PPS): All subjects who have completed the treatment prescribed by the protocol or who have not significantly violated the protocol are included in the per protocol set. The exact definition of a serious protocol deviation will be finalized at the time of data auditing, which may include but is not limited to the following circumstances.

1) Serious violation of inclusion criteria that affects the efficacy evaluation;
2) Treatment that seriously interferes with efficacy evaluation after inclusion;
3) Serious violation of medication regimen, with a total dose intensity of < 80;
4) Poor compliance that seriously affects the efficacy evaluation;

5) Too long medication delaying during the treatment;

6) Lack of primary efficacy index, etc.

PPS is a secondary analysis set for efficacy evaluation.

Safety set (SS): All randomized subjects receiving more than one dose of the investigational product and one safety evaluation are included in the safety set. The safety evaluation of this study is based on the safety set.

8.3. Baseline analysis

Continuous variables were presented as median with interquartile range (IQR) and were compared with Wilcoxon test. Categorical variables were presented as frequency with proportion and were compared with chi-square test.

8.4. Efficacy analysis

All the analyses were performed in the intention-to-treat population. Missing data on the primary outcome was imputed with treatment policy strategy, composite variable strategies, and while on treatment strategies accounting for intercurrent events, as follows:

- Treatment policy strategy

For the following concomitant events: After the subject is successfully randomized and starts to receive the treatment, concomitant medication/treatment is used during treatment (see Section 5.8) and the investigator does not discontinue the treatment or let the subject withdraw from the study early based on the actual situation; or the subject does not withdraw from the study early despite treatment delay, interruption or discontinuation due to AEs; the subject's mRS score can only be obtained remotely due to the pandemic, The primary efficacy outcomes will be treated by the treatment policy strategy, that is, regardless of whether the concomitant events occur. Whether the mRS reaches the standard will be assessed according to the measured mRS score collected (the mRS score of remote visit is considered valid).

- Composite strategy

For the following concomitant events: Randomized subjects, for whatever reason, do not have an mRS score after the start of the treatment;; or the main reason for the subject's early withdrawal from follow-up prescribed by the protocol is AE, the primary efficacy indexes will be treated by the composite strategy, i.e., the occurrence of such events is considered as

having a mRS score of 6 at the end of follow-up (Day 90 after treatment).

- While on treatment strategy

When a subject discontinues the treatment or withdraws from the study early for reasons other than those mentioned in the composite index strategy, the primary efficacy indexes will be treated by the while on treatment strategy, that is, using the most recent mRS score before the withdrawal from the study as the basis for determining whether the mRS meets the target.

Group difference in the primary efficacy outcome was examined using chi-square test or Fisher exact test, and the corresponding 95% confidence intervals (CIs) of the difference between proportions were estimated based on the normal-approximation. Odds ratios (ORs) with 95% CIs were calculated using logistic regression.

Unblinding sample size will be re-estimated in the interim analysis. If sample size adjustment occurs, the CHW method[13] will be used to weight the test statistics in the final analysis, and the *P* value of the hypothesis testing will be estimated accordingly for statistical inference.

Similar approaches were used for binary secondary outcomes, including mRS score ≤2 on day 90, NIHSS score ≤1 on day 14, 30, and 90, and safety outcomes on adverse events and treatment related adverse events. For mRS score on day 90, an ordinal logistic regression analysis was performed, with the results presented as common OR and 95% CI, where a common OR in favor of Y-2 sublingual tablet was >1.0. For changes in NIHSS score from baseline to day 14, means with 95% CIs were calculated for each group, and the mean differences with 95% CI between the groups were estimated by generalized linear regression.

In addition, a post hoc sensitivity analysis was performed using different approaches to impute missing data on the primary efficacy outcome. Finally, the treatment effects on the primary outcome were analyzed among several prespecified subgroups by including the interaction between treatment and subgroup effect into the logistic regression model.

8.5. Safety analysis

Data for safety evaluation include adverse events observed during the study and changes in laboratory data before and after treatment. Adverse event data are processed in statistical analysis after encoding according to the current version of MedDRA at the time of encoding.

Adverse events, adverse events during the study period since the first dose of the drug, major adverse events, unexpected adverse events, adverse event of special interest, serious adverse events, adverse events related to study drugs and serious adverse events related to study drugs are classified by system organ class and preferred term. Among them, significant adverse events are defined as adverse events that require drug therapy or non-drug therapy for the subject reporting the event; adverse events of special interest are divided into three types, i.e., liver function impairment, renal function impairment and hypokalemia. The Sponsor's clinical research associate is responsible for confirming and classifying all adverse events before unblinding.

If multiple adverse events occur in the same subject, the incidence rate is counted as 1 case; When the same AE occurs multiple times, the incidence of this AE is counted as 1. Adverse events will be summarized in frequency tables according to the system organ class (SOC) and preferred term (PT). The incidence is calculated by system, symptom/sign (count of cases: number of subjects who have experienced at least one adverse event).

The list of subjects experiencing adverse events and the list of subjects experiencing SAEs are provided. With respect to the laboratory data, in addition to the mean comparison before and after treatment, details of cases that are normal before treatment and abnormal after treatment or cases with abnormalities before treatment and abnormality exacerbation after treatment are mainly analyzed and listed. Refer to the statistical analysis plan for other details.

8.6. Interim analysis

An interim analysis is scheduled to be performed after approximately 50% of subjects complete the visit on Day 90. The purpose of interim analysis is to reestimate the sample size based on primary efficacy indexes. It is conducted by the Independent Data Monitoring Committee (IDMC) and whether the sample size should be increased or remain unchanged is recommended according to the regulations of the IDMC. The sample size may be increased to 1.5 times the scheduled sample size at most, and won't be reduced.

The re-estimation of sample size in the interim analysis in this study is performed based on the promising zone method of conditional power. Based on the published literature[13,14], in

order to realize the adjustment of type I error rate in the final analysis and control the overall type I error probability of the study at unilateral $\alpha \leq 0.025$, the statistics of the final analysis will be weighed based on the statistics obtained by CHW methods in the interim analysis and thereafter if sample size adjustment occurs, so as to realize the correction of the P value calculation. Refer to the Statistical Analysis Plan and IDMC Charter for details on the statistical method and decision-making process of sample size re-estimation in interim analysis.

An Independent Data Monitoring Committee (IDMC) will be established in the study, and the membership and operating rules of IDMC will be detailed in the IDMC Charter. The interim analysis will be provided to IDMC by a third-party statistical analysis team that is relatively independent of the Sponsor. The IDMC will perform data evaluation in accordance with the IDMC Charter, and provide the decision-making suggestions for the interim analysis (sample size increasing or unchanging) to the Sponsor's decision-making level, and the Sponsor's decision-making level will make decisions. During this process, the clinical investigators, subjects, and the Sponsor's team members do not accept any data or information from interim analysis after blinding, for maintaining blind state and reducing bias.

9. Supporting Documents and Relevant Considerations for Clinical Practice

9.1. Data management

9.1.1. Data acquisition

According to the study flow table, at each scheduled visit time, the investigator will record all important observations in the original medical record, at least including the following items:

- The name of the visit in the study flow chart and the actual date of visit
- Subject's general condition and status, including any important medical findings, such as AEs.
- Previous / concomitant medication / therapy

Follow-up visits for subjects by telephone or other means will also be recorded in the original medical records.

The information in the original medical record needs to be transcribed to the appropriate section of the CRF in a timely manner.

Changes in the information on the original medical records and other original documents must be signed and dated by the investigator or his/her designee on the day of the change. A brief explanation of the change should be made near the change if necessary.

9.1.2. Data recording

Important information about each subject in the clinical trial should be recorded in the CRF, and the investigator should review and agree to the completed CRF, and sign the name and date. The investigator's signature is to certify that the investigator ensures the integrity, accuracy, and authenticity of clinical and laboratory data entry in the case report form. If electronic CRFs are adopted in the study, the review and approval/signature of electronic CRFs will be carried out through an electronic data capture (EDC) system.

Source data refers to all information recorded on the original records or certified copies of clinical trials, including clinical findings, observations, and other relevant activities required to reconstruct and evaluate clinical trials. Source files refer to the original records, documents and data generated in clinical trials, such as hospital medical records, medical images, laboratory records, memoranda, subject diaries or evaluation forms, drug dispensing records, data automatically recorded by instruments, microfiche, photographic plates, magnetic media, X-rays, subject files, and clinical trial-related documents and records kept by pharmacies, laboratories and medical technology departments, including certified copies, etc. Source files include source data, which can exist in paper or electronic form. The information recorded in the case report form should be consistent with the source data recorded in the original records.

The investigator is required to verify that the data entered in the CRF is accurate, complete, readable and timely.

The completion, modification and replacement of all CRFs must be carried out by the investigator or other authorized person. If necessary, the Query generated manually or automatically by the system is loaded in the EDC system. The investigator or other authorized person must correct the CRF (if applicable) and complete the answer to the Query.

If the eCRF needs to be corrected after completion, it can be done in but not limited to the following three ways:

(1) The investigator actively modifies or solves Query on EDC systems using EDC tools.

(2) The clinical research associate generates a Query for the investigator to answer.

(3) The clinical data manager generates a Query for the investigator to answer.

9.1.3. Database locking

The database can be locked when the following conditions are met.

- All data has been collected and stored;
- All medical codes have been checked and confirmed;
- All queries about the data have been resolved;
- The database has passed the quality control test;
- The source data verification has been completed;
- The consistency check of the third-party data has been completed (if any);
- The consistency check of serious adverse events has been completed;
- It has been signed by all investigators;
- Analyzable cases have been defined;
- The statistical analysis plan has been signed.

9.1.4. Data archiving

After the completion of the study, if the electronic CRF is adopted in the study, the subject eCRF needs to be generated by the EDC system and stored on a non-rewritable CD, which will be kept by the Sponsor and all institutions for audit and/or inspection, respectively.

The preservation and management of study data must be carried out in accordance with GCP requirements, and necessary documents for clinical trials should be kept until 5 years after the investigational product is approved for marketing; For clinical trials that have not been used to apply for drug registration, the necessary documents should be kept for at least 5 years after the termination of the clinical trials.

9.2. Ethics

9.2.1. Responsibilities of the investigator

The investigator is responsible for ensuring that the clinical trial is conducted in

accordance with the clinical protocol, current GCP and relevant laws and regulations.

GCP is an internationally recognized ethical and scientific quality specification for the design, implementation, recording and reporting of clinical studies in humans. Studies that comply with this specification are considered to be consistent with the principles set forth in the *Declaration of Helsinki* in protecting the rights, safety and interests of subjects, and the data from the studies is trustworthy.

9.2.2. Ethical requirements

The study is carried out in accordance with the *Good Clinical Practice* (GCP), the *Declaration of Helsinki*, relevant regulations and the review opinions of the Ethics Committee.

The investigator should ensure that the study is reviewed and approved by a qualified Ethics Committee that meets the requirements of the GCP. Prior to the study, the investigator should submit the study protocol, informed consent form and other required materials to the Ethics Committee for review and approval. The Sponsor can only provide the investigational product after receiving approval from the Ethics Committee. At the same time, the Ethics Committee must be informed of follow-up protocol supplements that may affect the safety of the subjects and continued participation in the study, and serious adverse events that occur during the study. The investigator is responsible for reporting the progress of the study to the Ethics Committee. In addition, the investigator must promptly provide the Sponsor with copies of all communications with the Ethics Committee. When reviewing and approving the study protocol, the Ethics Committee must confirm the protocol title, the protocol number, and indicate the protocol document reviewed and the review date. During the study, if there are any new modifications to the study protocol, informed consent form, etc., the written approval opinions of relevant administration units should be obtained again in accordance with the regulations.

9.2.3. Ethical norms for the study

This study protocol needs to be reviewed and approved in writing by the Ethics Committee of the hospital before implementation. The Ethics Committee should be provided with the study protocol, protocol amendment, informed consent form, and other relevant

documents such as recruitment advertisements. This clinical trial shall comply with the *Declaration of Helsinki*, the *Good Clinical Practice* (GCP) issued by the National Medical Products Administration (NMPA) and related regulations. Approval from the hospital's Ethics Committee is required before the start of the study.

Without the consent of both the Sponsor and the investigator, neither party is allowed to unilaterally modify the study protocol. Only in order to eliminate direct, immediate harm to the subject, the investigator may make changes or deviations to the study protocol prior to approval by the Ethics Committee/Institutional Review Board. At the same time, the deviations or changes made, the reasons for them, and the proposed amendments should be submitted to the Ethics Committee/Institutional Review Board for deliberations as soon as possible. The investigator must explain and document any protocol deviations made.

During the clinical study, any modifications to this protocol should be submitted to the Ethics Committee and, if necessary, other study documents should be modified accordingly, and submitted and/or approved as required by the Ethics Committee. The investigator is responsible for submitting the interim report of the study regularly according to the relevant requirements of the Ethics Committee, and should notify the Ethics Committee of the end of the study after it's completed.

9.2.4. Informed consent of subjects

Informed consent is a step that begins before an individual consents to participate in a study and continues throughout his/her participation in the study. The informed consent form needs to be approved by the Institutional Review Board / Ethics Committee (IRB/IEC) and subjects will be required to read and understand the document. The investigator will explain the study to subjects and answer any questions the subjects may have. The investigator will explain verbally to subjects in a way that is appropriate to subjects' understanding: the objective, process, and potential risks of the study, as well as the rights and interests of subjects. Subjects should have sufficient time to carefully read and ask questions before signing the written informed consent form. Subjects should have the opportunity to discuss the study with their family or surrogate or consider it for themselves before agreeing to participate in the study. Subjects will sign the informed consent form before proceeding with

any process specific to the study. Subjects must be informed that participation in the study is voluntary and that they can withdraw from the study at any time without harm. The investigator will provide subjects with a copy of the informed consent document for their preservation. The informed consent process should precede the subject's acceptance of any procedures for the study. The process of informed consent (including dates) should be documented in the source file, and the signed informed consent form should be kept. The investigator must specifically inform subjects that "The quality of medical care for them will not be adversely affected if they refuse to participate in this study" to ensure the rights and welfare of the subjects.

The investigator or qualified designee must obtain written informed consent form from each subject or the legal guardian of each subject.

9.2.5. Confidentiality of subject information

The collected and processed personal data of subjects is only used to study the efficacy, safety, quality and use of the investigational product.

Adequate actions are taken to collect and process data to ensure the data confidentiality and compliance with current laws and regulations on the protection of privacy. Appropriate technical methods and management measures are adopted to protect personal data from breach or unauthorized disclosure or access, accidental or unlawful destruction, accidental loss or tempering. Personnel from the Sponsor with access to personal data are required to maintain the confidentiality of the identity of subjects in the study.

The Sponsor ensures that personal data is:

- Processed in a fair and legal manner;
- Collected for legal purposes stated in the protocol and not processed in any other manner contrary to the purposes.
- Adequate, relevant and appropriate for the purposes stated;
- Accurate and kept up to date where necessary.

9.3. Quality assurance and quality control

9.3.1. Quality control of the study implemented by the Sponsor

1) Set up clinical study quality assurance measures and quality control systems to ensure

the quality of the clinical study;

2) Conduct clinical trials in strict accordance with GCP and protocol requirements to ensure that the study is in line with scientific and ethical principles;

3) Fully respect the authenticity of clinical study data, and do not modify the test data in any way;

4) During the study, send clinical research associates to conduct regular inspection visits to each site to check the informed consent of subjects, the preservation of original data, the filling of electronic case report form, the use and storage of the investigational product, the presence of adverse events and handling, etc., so as to make the implementation process of the study strictly follow the requirements of GCP and other regulations;

5) Send inspectors to review the conduct of the clinical trial if necessary.

9.3.2. Quality control of the study implemented by the investigator

1) The investigator assumes the duties and functions related to the clinical trial. It should be guaranteed that the investigator has the corresponding qualification, and complete procedures have been established to ensure that the investigator performs the duties and functions related to clinical trials, thus producing reliable data. The consent of the Sponsor should be obtained for authorizing a unit other than the clinical site to undertake study-related duties and functions;

2) The investigator should conscientiously implement informed consent, patiently explain to subjects to make them fully understand and cooperate with the study; strengthen health care guidance and monitor subjects' medication compliance;

2) Strictly implement the study protocol, do not use the investigational product for other non-investigational purposes, and carefully record the results of various tests, as well as the adverse events or serious adverse events during the study;

3) During the implementation of the study, it is best to complete the evaluation of various observation indexes of the same subject by the same investigator to reduce subjective bias;

4) After adverse events and/or serious adverse events occur in subjects, the investigator

should actively guide and/or treat the subjects to protect the rights and interests of them;

5) The investigator or the person authorized by the investigator (such as the clinical research coordinator) should record the content of the eCRF truthfully, detailedly and carefully according to the requirements for eCRF filling to ensure that the content is true and reliable;

6) In view of possible dropout, active actions should be taken to control the dropout rate of cases within 20%.

9.3.3. Quality control monitoring

According to the regulations of GCP, the clinical research associate should conscientiously perform the following duties:

1) Ensure that the rights and interests of subjects in the clinical trial are protected, the study data recorded and reported are accurate and intact, and the study follows the approved protocol and relevant regulations.

2) Have an appropriate degree in medicine, pharmacy or related majors, and have undergone necessary training, familiar with the relevant regulations of drug administration, familiar with the preclinical and clinical information about the investigational product as well as the clinical trial protocol and related documents.

3) Follow standard operating procedures and supervise the implementation of the clinical trial to ensure that the clinical trial is performed according to protocol. The specific content includes:

- ✧ Prior to the study, confirm that the Sponsor has appropriate conditions including staffing and training; the laboratory is fully equipped and in good operation, and has all kinds of examination conditions related to the study; a sufficient number of subjects are estimated, and the personnel participating in the study are familiar with the requirements of the protocol.
- ✧ During the study, monitor the investigator's implementation of the protocol, confirm that the informed consent forms of all subjects have been obtained before the study, understand the enrollment rate of the subjects and the study

progress, and confirm that the enrolled subjects are eligible;

- ✧ Verify that all data is recorded and reported correctly and completely, all the case report forms are filled correctly and consistent with the original data. Verify that all errors or omissions have been corrected or noted, signed and dated by the investigator. Confirm and record the treatment change, concomitant medication, intermittent diseases, loss to follow-up, and omission of examination in all subjects. Verify whether the withdrawal and loss to follow-up of enrolled subjects have been described in the eCRF;
- ✧ Confirm that all adverse events are recorded, and serious adverse events are reported and recorded within the specified time;
- ✧ Verify that the investigational product is supplied, stored, distributed, and returned in accordance with relevant regulations, and make corresponding records;
- ✧ Assist the investigator with necessary notification and application matters, and report the study data and results to the Sponsor;
- ✧ Clearly and truthfully record the follow-ups that the investigator fails to perform, the trials and inspections that are not performed, and the correction of errors or omissions;
- ✧ Complete a written report after each visit and submit to the Sponsor, which should contain content including the monitoring date, time, name of the clinical research associate, monitoring findings, etc.

9.3.4. Data quality control

1) Authenticity of the source data: During the clinical trial, the clinical research associate will conduct regular on-site inspection visits to each site to ensure that all content of the study protocol is strictly complied with and that the source data is authentic and reliable.

2) Consistency of eCRF and source data: The eCRF is verified by clinical research associate to ensure that the content in the eCRF is consistent with the original data records.

3) Accuracy of data entry: All kinds of errors related to the filling and calculation are eliminated through the programmed check of the numerical range and logic of the database.

9.4. Data preservation and confidentiality

- To ensure the evaluation and supervision of clinical study by the NMPA and the Sponsor, the investigator should agree to keep all study data, including the original documents of hospitalization, informed consent forms, case report forms, detailed records of drug distribution, etc. The original records and related data will be kept in a special file cabinet with a lock for at least 5 years after the investigational product is approved for production. Non-investigator and unauthorized personnel are not allowed to check the data, and it should be locked into the file cabinet in time after use. The original records are electronically saved in a special computer through photos, and viewing permission is set. All data and information in this clinical study is owned by Jiangsu Simcere Pharmaceutical Group Limited. The documents of the site should not be damaged without prior written agreement between the investigator and the Sponsor. If the investigator chooses to provide the study documentation to another party or transfer it elsewhere, the Sponsor must be informed in advance. Unless requested by the National Medical Products Administration, the investigator shall not provide the information to a third party in any form without the written consent of the Sponsor.
- During statistical analysis, only the subject code is displayed and used as a unique identifier for the subject's identification;
- Subject's personal information will be kept confidential in accordance with regulations and will not appear in publications and conference reports.

9.5. Protocol revision

During the study process, the investigator should obtain the consent and approval of the Sponsor to revise the protocol, and the Sponsor should designate relevant personnel for revision.

The revised protocol must be submitted to the Ethics Committee for approval. The investigator still follows the original study protocol until it is approved by the Ethics

Committee, unless the protocol revision involves immediate elimination of potential harm to the subjects, or the protocol revision involves only changes in study administration (such as a change in telephone numbers, etc.)

9.6. Protocol deviation

In the absence of pre-review and consent granted by the Sponsor or the Sponsor's agent, the investigator shall not violate the protocol unless it is necessary to eliminate immediate harm to subjects in the study under the premise of following the EC and local regulations. When a violation resulting from protocol implementation is deemed necessary for an individual subject, the investigator must contact the Sponsor as soon as possible to obtain the Sponsor's review and confirm the impact of the violation on subjects and/or the study. Any major protocol violation that affects the subject's eligibility and/or safety is subject to the review and/or approval by EC and regulatory authorities; It should be implemented before the protocol implementation if feasible.

9.7. Publication of study results

The results of this study may be published in scientific congresses and scientific journals, and may be signed by principal investigators who have made significant contributions to the implementation and management of this study and personnel who have made significant contributions to the design, interpretation or analysis of this study (such as employees or consultants of Jiangsu Simcere Pharmaceutical Group Limited.). The authorship principles will be based on the requirements for authorship from the International Committee of Medical Journal Editors (ICMJE).

The Company promises to provide the article before publication to the investigator for review before publishing any findings of the study. The investigator is required to obtain the consent of the Sponsor before submitting academic articles or abstracts about this study. The investigator has the right to publish the findings of this study, subject to the protection of confidential information.

10. References

1. Motwani JG, Lipworth BJ: Clinical Pharmacokinetics of Drugs Administered Buccally and Sublingually. Clin Pharmacokinet 1991 Aug; 21:83–94.

2. Sato T, Mizuno K, Ishii F: A novel administration route of edaravone--II: mucosal absorption of edaravone from edaravone/hydroxypropyl-beta-cyclodextrin complex solution including L-cysteine and sodium hydrogen sulfite. Pharmacology 2010;85:88–94.
3. Investigators EAST: Use of anti-ICAM-1 therapy in ischemic stroke: Results of the Enlimomab Acute Stroke Trial. Neurology 2001 Oct 23;57:1428–1434.
4. Yamagishi, K., et al., Plasma fatty acid composition and incident ischemic stroke in middle-aged adults: the Atherosclerosis Risk in Communities (ARIC) Study. Cerebrovasc Dis, 2013. 36(1): p. 38-46.
5. 《中国急性缺血性卒中诊治指南》2018 年版
6. 《中国缺血性脑卒中和短暂性脑缺血发作二级预防指南》2014 年版
7. Lees KR, et al. N Engl J Med. 2006 Feb 9;354(6):588-600. NXY-059 for Acute Ischemic Stroke
8. Shuaib A, et al. N Engl J Med. 2007 Aug 9;357(6):562-71. NXY-059 for the Treatment of Acute Ischemic Stroke
9. Martin RH, et al. Stroke. 2016 Sep;47(9):2355-9. ALIAS (Albumin in Acute Ischemic Stroke) Trials Analysis of the Combined Data From Parts 1 and 2
10. Ehrenreich H, et al. Stroke. 2009 Dec;40(12):e647-56. Recombinant Human Erythropoietin in the Treatment of Acute Ischemic Stroke
11. Lees KR, et al. Stroke. 2013 Mar;44(3):580-4. Results of Membrane-activated Chelator Stroke Intervention Randomized Trial of DP-b99 in Acute Ischemic Stroke
12. Zivin JA, et al. Stroke. 2009 Apr;40(4):1359-64. Effectiveness and Safety of Transcranial Laser Therapy for Acute Ischemic Stroke
13. Cui L, Hung HM, Wang SJ. Modification of sample size in group sequential clinical trials. *Biometrics* 1999; **55**:853--857.
14. Mehta CR, Pocock SJ. Statistics in Medicine 2011, 30:3267-3284. Adaptive Increase in Sample Size when Interim Results are Promising: A Practical Guide with Examples

11. Appendices

Appendix 1 Key Points in Diagnosis of Cerebrovascular Diseases (Excerpt)

I. Transient Ischemic Attack

1. Sudden focal brain or retinal dysfunction, which is consistent with the ischemic manifestations of carotid or vertebral-basilar system, generally recovers completely within 24 hours (most do not exceed 1 h), and may be recurrent.
2. Diffusion-weighted imaging (DWI) of cranial MRI indicates no evidence of acute cerebral infarction, and it is a transient ischemic attack confirmed by imaging. When DWI is performed unconditionally, no corresponding infarct lesion is found in the routine sequence of cranial CT/MRI, which can be used as a basis for clinical diagnosis. When radiographic evidence of responsible foci is not available, the duration of symptoms/signs not more than 24 hours is still regarded as the time limit.
3. Non-ischemic causes are excluded.

II. Stroke

(I) Subarachnoid hemorrhage

(1) Sudden severe headache, which may be accompanied by nausea, vomiting, limb convulsions or varying degrees of disturbance of consciousness, meningeal stimulation sign positive.

(2) Cranial CT/MRI or lumbar puncture shows bloody cerebrospinal fluid in the subarachnoid space.

(3) Clinical or auxiliary examinations confirm the cause associated with this hemorrhage or unknown cause, excluding secondary or traumatic subarachnoid haemorrhage due to other causes.

(II) Cerebral hemorrhage

(1) Sudden focal neurofunction deficit or headache, vomiting, varying degrees of disturbance of consciousness.

(2) Cranial CT/MRI shows intracerebral hemorrhage lesion.

(3) Secondary or traumatic cerebral hemorrhage due to other causes is excluded.

(III) Ischemic stroke (cerebral infarction)

1. Atherosclerotic cerebral infarction

(1) There may be risk factors for atherosclerosis or evidence of systemic atherosclerosis;

(2) Cranial CT/MRI shows single or multiple scattered lesions involving the cerebral cortex, often exceeding the territory supplied by a single blood vessel, with a tendency of hemorrhagic transformation.

(3) Evidence of source of emboli in heart, or other concomitant organ embolisms may be found.

2. Cardioembolic cerebral infarction

(1) It usually occurs abruptly during activity, with immediate peak neurological deficits;

(2) Cranial CT/MRI shows single or multiple scattered lesions involving the cerebral cortex, often exceeding the territory supplied by a single blood vessel, with a tendency of hemorrhagic transformation.

(3) Evidence of source of emboli in heart, or other concomitant organ embolisms may be found.

3. Lacunar infarction

(1) It is often manifested as lacunar syndrome, unconsciousness disorder, or cortical involvement;

(2) Cranial CT/MRI confirmed that there are corresponding lacunar infarcts, most of which are $\leq$ 1.5 cm in diameter and mainly located in the deep white matter, basal ganglia, thalamus or pons;

(3) Cerebral artery imaging shows no significant stenosis of the corresponding cerebral artery, or high-resolution MRI shows no definite atherosclerotic plaque blockage at the opening of the perforating artery.

4. Cerebral infarction due to other causes

(1) Generally, there is no clear common risk factors for cerebrovascular diseases such as hypertension, atherosclerosis or heart disease;

(2) It is manifested as acute onset of focal or general neurological deficits;

(3) Vascular imaging or hematological examination reveals diseases such as moyamoya, cerebral arteritis, cerebral artery dissection, polycythemia vera, thrombocytosis or hypercoagulable state, and confirms that it is related to this cerebral infarction.

5. Cerebral infarction of unknown causes

(1) Generally, there is no risk factors for common cerebrovascular diseases such as hypertension, atherosclerosis or heart disease;

(2) It is manifested as acute onset of focal or general neurological deficits;

(3) No clear cause is detected through a comprehensive standard examination, or two or more causes are found but the true cause cannot be determined, or the causes are not found due to insufficient auxiliary examinations.

-- Excerpted from the *Key Points in Diagnosis of Cerebrovascular Disease*s from the 4th National Conference on Cerebrovascular Diseases organized by the Chinese Medical Association.

Appendix 2 Modified Rankin Scale

Patient's performance	Score
No symptoms	0
No significant disability despite symptoms; able to carry out all usual duties and activities	1
Slight disability: unable to carry out all previous activities but able to look after own affairs without assistance	2
Moderate disability: requiring some help, but able to walk independently	3
Moderately severe disability: unable to walk independently, and unable to attend to own bodily needs without assistance	4
Severe disability: bedridden, incontinent, and requiring constant nursing care and attention	5

The Modified Rankin Scale is used to rate the outcome of functional recovery in patients after stroke. The formal definition of each level is shown in **boldface.** Further guidance to reduce possible errors between different observers is shown in **italics**, but there are no requirements for the structure of the interview. Note that only symptoms that have occurred since stroke are considered. Patients are considered to be able to walk independently if they can walk with the help of certain auxiliary devices without outside assistance.

If both levels seem equally applicable to the patient, and further questioning is unlikely to result in an absolutely correct choice, the more severe level should be chosen.

0-No symptoms at all

Although mild symptoms may appear, the patient is not aware of any new functional limitations and symptoms since the stroke.

1- No significant disability despite symptoms; able to carry out all usual duties and activities

The patient has certain symptoms caused by stroke, either physical or cognitive (e.g., affecting speech, reading and writing; Or physical movement; Or feel; Or vision; Or swallow; Or emotional), but may continue to engage in all work, social and leisure activities prior to stroke.

The key questions used to distinguish between levels 1 and 2 (see below) could be, "Are there things that you used to do but cannot until after stroke?". Activities that are more frequent than once a month are considered "frequent".

2-Slight disability; unable to carry out all previous activities, but able to look after own affairs without assistance

After stroke, the patient can no longer perform activities that were possible before stroke (e.g., driving, dancing, reading or working), but he/she can still take care of himself/herself on a daily basis without assistance from others. The patient can dress, walk, eat, go to the bathroom, prepare simple food, go shopping and travel locally without the help of others. The patient can live without supervision. It is envisaged that patients at this level may be left alone at home for a week or longer time without care.

3-Moderate disability; requiring some help, but able to walk independently

Patients at this level can walk independently (with the help of walking aids) and can dress, go to the bathroom and eat independently, but need the assistance from others for more complex tasks. For example, someone else is needed to do the shopping, cooking or cleaning, and to visit the patients more than once a week to ensure these activities are completed. Assistance is needed not only in taking care of the body, but also in giving advice: For example, patients at this level will need supervision or encouragement to handle finances.

4-Moderately severe disability; unable to walk and attend to bodily needs without assistance

The patient needs help with his/her daily life, whether it's walking, dressing, going to the bathroom, or eating. The patient needs to be cared for at least once a day and often twice or more, or must live close to the caregiver. To distinguish between levels 4 and 5 (see below), whether the patient is able to live alone at an appropriate time of day is considered.

5-Severe disability; bedridden, incontinent and requiring constant nursing care and attention

Although no trained nurse is required, someone is required to look after the patient several times throughout the day and night.

Death is assigned to the score of 5 in this study.

Appendix 3 NIH Stroke Scale (NIHSS)

Record performance in each category after each subscale exam. Do not go back and change scores. Scores should reflect what the patient does, not what the clinician thinks the patient can do. The clinician should record answers while administering the exam and work quickly. Except where indicated, the patient should not be coached (i.e., repeated requests to patient to make a special effort). Some items that are not assessed should be detailed in the form.

Handedness: ○1-Left ○2-Right ○99-Unknown			
	Instructions	Score	Score
1a	Level of Consciousness:<br>The investigator must choose a response if a full evaluation is prevented by such obstacles as an endotracheal tube, language barrier, orotracheal trauma/bandages. A 3 is scored only if the patient makes no movement (other than reflexive posturing) in response to noxious stimulation.	0 = Alert; keenly responsive.<br>1 = Not alert; but arousable by minor stimulation to obey, answer, or respond.<br>2 = Not alert, or is obtunded and requires strong or painful stimulation to make movements (not stereotyped).<br>3 = Responds only with reflex motor or autonomic effects or totally unresponsive, flaccid, and areflexic.	_____
1b	LOC Questions:<br>(Score initial responses only, and no prompt from the investigator)<br>The patient is asked the month and his/her age. The answer must be correct - there is no partial credit for being close. Aphasic and stuporous patients who do not comprehend the questions will score 2. Patients unable to speak because of endotracheal intubation, orotracheal trauma, severe dysarthria from any cause, language barrier, or any other	0 = Answers both questions correctly.<br>1 = Answers one question correctly.<br>2 = Answers neither question correctly.	_____

	problem not secondary to aphasia are given a 1.		
1c	LOC Commands:<br>The patient is asked to open and close the eyes and then to grip and release the non-paretic hand. Use another command (put the tongue out) if the hands cannot be used. Credit is given if an unequivocal attempt is made but not completed due to weakness. If the patient does not respond to the command, the task should be demonstrated to him or her, and the result scored. Patients with trauma, amputation, or other physical impediments should be given suitable one-step commands.	0 = Answers both questions correctly.<br>1 = Performs one task correctly.<br>2 = Performs neither task correctly.	_____
2	Best Gaze:<br>Only horizontal eye movements will be tested. Voluntary or reflexive (oculocephalic) eye movements will be scored. If the patient has a conjugate deviation of the eyes that can be overcome by voluntary or reflexive activity, the score will be 1. If a patient has an isolated peripheral nerve paresis (CN III, IV or VI), score a 1. Gaze is testable in all aphasic patients. Patients with ocular trauma, bandages, pre-existing blindness, or other disorder of visual acuity or fields should be tested with reflexive movements, and a	0 = Normal.<br>1 = Partial gaze palsy; gaze is abnormal in one or both eyes, but forced deviation or total gaze paresis is not present.<br>2 = Forced deviation, or total gaze paresis not overcome by the oculocephalic maneuver.	_____

	choice made by the investigator. Establishing eye contact and then moving about the patient from side to side will occasionally clarify the presence of a partial gaze palsy.		
3	Visual:<br>Visual fields (upper and lower quadrants) are tested by confrontation, using finger counting or visual threat, as appropriate. Patients may be encouraged, but if they look at the side of the moving fingers appropriately, this can be scored as normal. If there is unilateral blindness or enucleation, visual fields in the remaining eye are scored. Score 1 only if a clear-cut asymmetry, including quadrantanopia, is found. If patient is blind from any cause, score 3. Double simultaneous stimulation is performed at this point. If there is extinction, patient receives a 1, and the results are used to respond to item 11.	0 = No visual loss.<br>1 = Partial hemianopia.<br>2 = Complete hemianopia.<br>3 = Bilateral hemianopia (blind including cortical blindness).	____
4	Facial Palsy:<br>Ask - or use pantomime to encourage - the patient to show teeth or raise eyebrows and close eyes. Score symmetry of grimace in response to noxious stimuli in the poorly responsive or non-comprehending patient. If facial trauma/bandages, orotracheal tube, tape or other physical barriers obscure the	0 = Normal.<br>1 = Minor paralysis (flattened nasolabial fold, asymmetry on smiling).<br>2 = Partial paralysis (total or near-total paralysis of lower face).<br>3 = Complete paralysis of one or both sides (absence of facial movement in the upper and lower face).	____

	face, these should be removed to the extent possible.		
5	Motor Arm:<br>The limb is placed in the appropriate position: extend the arms (palms down) 90 degrees (if sitting) or 45 degrees (if supine). Drift is scored if the arm falls before 10 seconds. The aphasic patient is encouraged using urgency in the voice and pantomime, but not noxious stimulation. The investigator can lift the patient's upper limb to the required position and encourage the patient to persevere. Only the affected side was assessed.	0 = No drift; limb holds in the appropriate position for full 10 seconds.<br>1 = Drift; limb drifts down before full 10 seconds; does not hit bed or other support.<br>2 = Limb cannot get to or maintain (if cued) 90 (or 45) degrees, drifts down to bed, but has some effort against gravity.<br>3 = No effort against gravity; limb falls.<br>4 = No movement.<br>9 = Amputation or joint fusion, explain: __________	5a. Left Arm ____<br>5b. Right Arm ____
6	Motor Leg:<br>Hold the leg at 30 degrees (always tested supine). Drift is scored if the leg falls before 5 seconds. The aphasic patient is encouraged using urgency in the voice and pantomime, but not noxious stimulation. The investigator can lift the patient's upper limb to the required position and encourage the patient to persevere. Only the affected side was assessed.	0 = No drift; leg holds in the appropriate position for full 5 seconds.<br>1 = Leg falls by the end of the 5-second period but does not hit bed.<br>2 = Leg falls to bed by 5 seconds, but has some effort against gravity.<br>3 = No effort against gravity; leg falls to bed immediately.<br>4 = No movement.<br>9 = Amputation or joint fusion, explain: ____________	6a. Left Leg _____<br>6b. Right Leg _____
7	Limb Ataxia:<br>This item is aimed at finding evidence of a unilateral cerebellar lesion. Test with eyes open. In case of visual defect, ensure testing	0 = Absent.<br>1 = Present in one limb.<br>2 = Present in two limbs.<br>9 = Amputation or joint fusion, explain:	_____

	is done in intact visual field. The finger-nose-finger and heel-shin tests are performed on both sides, and ataxia is scored only if present out of proportion to weakness. Ataxia is absent in the patient who cannot understand or is paralyzed. In case of blindness, test by having the patient touch nose from extended arm position. Only in the case of amputation or joint fusion, the examiner should record the score as 9, and clearly write the explanation for this choice.	____________	
8	Sensory:<br>Test with pin. Sensation or grimace to pinprick when tested, or withdrawal from noxious stimulus in the obtunded or aphasic patient. Only sensory loss attributed to stroke is scored as abnormal. The examiner should test as many body areas (arms [not hands], legs, trunk, face) as needed to accurately check for hemisensory loss. A score of 2, "severe or total sensory loss" should only be given when a severe or total loss of sensation can be clearly demonstrated. Stuporous and aphasic patients will, therefore, probably score 1 or 0. The patient with brainstem stroke who has bilateral loss of sensation is scored 2. If the patient does not respond and is quadriplegic,	0 = Normal; no sensory loss.<br>1 = Mild-to-moderate sensory loss; the patient feeling pinprick is less sharp or is dull on the affected side; or there is a loss of superficial pain with pinprick, but patient is aware of being touched.<br>2 = Severe to total sensory loss; patient is not aware of being touched in the face, arm, and leg.	_____

	score 2. Patients in a coma (item 1a=3) are automatically given a 2 on this item.		
9	Best Language:<br>Name and reading examination. The patient is asked to name the items on the attached naming sheet and to read from the attached list of sentences. Comprehension is judged from responses here, as well as to all of the commands in the preceding general neurological exam. If visual loss interferes with the tests, ask the patient to identify objects placed in the hand, repeat, and produce speech. The intubated patient should be asked to write. The patient in a coma (item 1a=3) will automatically score 3 on this item. The examiner must choose a score for the patient with stupor or limited cooperation, but a score of 3 should be used only if the patient is mute and follows no one-step commands.	0 = No aphasia; normal.<br>1 = Mild-to-moderate aphasia; some obvious loss of fluency or facility of comprehension, without significant limitation on ideas expressed or form of expression.<br>2 = Severe aphasia; all communication is through fragmentary expression; great need for inference, questioning, and guessing by the listener. Range of information that can be exchanged is limited; listener carries burden of communication.<br>3 = Mute, global aphasia; no usable speech or auditory comprehension.	____
10	Dysarthria:<br>Do not tell the patient why he or she is being tested. Ask the patient to read or repeat words from the attached list. If the patient has severe aphasia, the clarity of articulation of spontaneous speech can be rated. Only if the patient is intubated or has other physical barriers to producing speech, the examiner should record the score as 9. Clearly write	0 = Normal.<br>1 = Mild-to-moderate dysarthria; patient slurs at least some words and, at worst, can be understood with some difficulty.<br>2 = Severe dysarthria; patient's speech is so slurred as to be unintelligible.<br>9 = Intubated or other physical barrier, explain:__________________	____

	an explanation for this choice.		
11	Extinction and Inattention (formerly Neglect):<br>If the patient has a severe visual loss preventing visual double simultaneous stimulation, and the cutaneous stimuli are normal, the score is normal. If the patient has aphasia but does appear to attend to both sides, the score is normal. The patient's ability to recognize cutaneous sensations and visual stimuli occurring simultaneously on the left and right sides is tested to determine whether the patient has inattention. Show the standard picture to the patient and ask him/her to describe it. The patient is encouraged to look at the picture carefully and identify the features on the right and left. If the patient is unable to recognize part of the picture, it is considered abnormal. Then, the patient is asked to close his/her eyes to test the upper or lower leg acupuncture sensation separately to check the bilateral cutaneous sensation. If the patients have sensory neglect on one side, it will be considered abnormal.	0 = No abnormality.<br>1 = Visual, tactile, auditory, spatial, or personal inattention or extinction to bilateral simultaneous stimulation in one of the sensory modalities.<br>2 = Profound hemi-inattention or extinction to more than one modality; does not recognize own hand or orients to only one side of space.	_____
12	**Additional item, not a part of the NIH Stroke Scale score.**<br>Distal motor function:<br>The patient's hand is held up at the forearm	0 = Normal (No flexion after 5 seconds)<br>1 = At least some extension after 5 seconds, but not fully extended. Any movement of the fingers which is not command is not scored.	Left Arm<br>_____<br>Right

	by the examiner and patient is asked to extend his/her fingers as much as possible. If the patient can't or doesn't extend the fingers the examiner places the fingers in full extension and observes for any flexion movement for 5 seconds. The patient's first attempts only are graded. Repetition of the instructions or of the testing is prohibited.	2 = No voluntary extension after 5 seconds. Movements of the fingers at another time are not scored.	Arm ____

Attached: Pictures for test items 9 and 10

Reading test picture

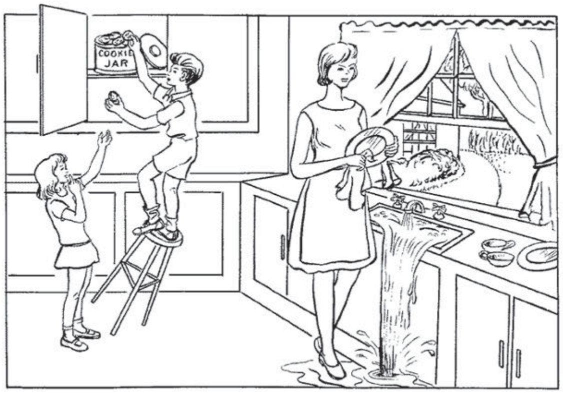

Reading test picture 2

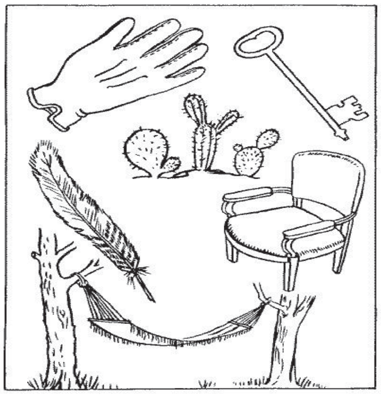

Reading test picture 3

Please read the following sentences:

You know how.

Down to earth.

Go home and cook.

Review lessons at school.

Make a wonderful speech.

Reading test picture 4

Please read the following the words:

MAMA

Earth

Plane

Silk

Start work on time

Spit no grape skins while eating grapes.

How to evaluate NIHSS score in coma patients?

Items should be evaluated one by one for patients with a score less than 3 in 1a.

When the patient has no response at all to any harmful stimuli (sternum rubbing, orbital compression, etc.) and responds only with reflex motor, the score of 1a is 3.

If 1a=3, other items should be scored as:

- 1b- LOC Questions: 2
- 1c- LOC Commands: 2

- 2-Best Gaze: It is scored according to whether it can be overcome by the oculocephalic reflex. The score is 1 if it can be overcome by the oculocephalic reflex, and 2 if not.
- 3-Visual: It is scored using perceived threats.
- 4- Facial Palsy: 3
- 5, 6-Motor Arm and Leg: 4 for each
- 7-Limb Ataxia: Only in the case of ataxia, the examiner should record the score. If the patient's muscle strength is reduced and he/she can't complete the examination of the fingers, nose, heels, knees and shins, it will be scored as 0.
- 8-Sensory: 2
- 9-Best Language: 3
- 10-Dysarthria: 2
- 11-Extinction and Inattention (formerly Neglect): Coma means loss of all cognitive abilities, so it will be scored as 2.

How to calculate the total score of NIHSS?

The following items should not be included in the total score when the total score is calculated:

- "9 = Amputation or joint fusion" in the 5, 6-Motor Arm and Leg
- Items identifying the ataxic site in the 7-Limb Ataxia, namely "Left upper limb 1 = Yes 2 = No 9 = Amputation or joint fusion, explain: "(Registration is not required to be filled).
- Additional item - Distal motor function. (Registration is not required to be filled)

Appendix 4 SSS TOAST Classification

I. Large Artery Atherosclerosis

- Evident (both)
  - ✧ Occlusion or stenosis ≥50% caused by atherosclerosis in the clinically relevant extra-/intracranial arteries
  - ✧ The absence of acute infarction in vascular territories other than the stenotic or occluded artery.
- Probable (one of the three)
  - ✧ History of ≥ 1 amaurosis fugax, TIA or stroke from the territory of the index artery affected by atherosclerosis within the last month.
  - ✧ Evidence of near-occlusive stenosis or non-chronic complete occlusion judged to be due to atherosclerosis in the clinically relevant extra-/intracranial arteries.
  - ✧ The presence of ipsilateral border-zone infarctions (BZI) or multiple infarctions only within the territory of the affected artery.
- Possible (one of the two)
  - ✧ History of ≥ 2 (at least one attack occurred within the last month) amaurosis fugax, TIA or stroke from the territory of index artery affected by atherosclerosis; Detectable atherosclerotic plaques in a clinically relevant extra-/intracranial artery that protruded into the lumen and led to mild stenosis (50%).
  - ✧ Evidence of extensive artery atherosclerosis in the absence of complete diagnostic investigation for other mechanisms.

II. Cardioaortic Embolism

- Evident
  - ✧ The presence of a high-risk cardiac source of cerebral embolism.
- Probable (one of the two)
  - ✧ Evidence of systemic embolism (including pulmonary embolism, mesenteric artery embolism, renal artery embolism, or skin embolism, etc.).
  - ✧ Presence of multiple acute infarctions that have occurred closely related in time within both right and left anterior or both anterior and posterior circulations in the

absence of occlusion or near-occlusive stenosis of all relevant vessels; other diseases that can cause multifocal ischemic brain injury such as vasculitides, vasculopathies, and hemostatic or hemodynamic disturbances must not be present.

- Possible (one of the two)
  - ✧ The presence of a cardiac condition with a low or uncertain risk of cerebral embolism.
  - ✧ Evidence of cardio-aortic embolism in the absence of complete diagnostic investigation for other mechanisms.

III. Small-Artery Occlusion

- Evident
  - ✧ Imaging evidence of a single, clinically relevant acute infarction < 20 mm in greatest diameter within the territory of penetrating arteries. Absence of significant pathology in the parent artery at the site of the origin of the penetrating artery (most commonly focal atherosclerosis, aortic dissection, vasculitis, vasospasm, etc.)
- Probable
  - ✧ Presence of a classical lacunar syndrome, stereotypic lacunar TIAs within the past week.
- Possible (one of the two)
  - ✧ Classical lacunar syndrome in the absence of imaging that is sensitive enough to detect small infarctions.
  - ✧ Evidence for small artery occlusion in the absence of complete diagnostic investigation for other mechanisms.

IV. Other Causes

- Evident
  - ✧ Presence of a specific disease process that involves clinically appropriate brain arteries.
- Probable

  - ✧ A specific disease process that has occurred in clear and close temporal relation to the onset of brain infarction, such as arterial dissection, cardiac or arterial surgery, and cardiovascular interventions.
- Possible
  - ✧ Evidence for an evident other cause of stroke in the absence of complete diagnostic evaluation for mechanisms listed above.

V. Undetermined Causes

- No identified cause (not meeting the "Evident" or "Possible" diagnostic criteria listed above)
  - ✧ Cryptogenic embolism (one of the three)
    1) Evidence of abrupt cutoff consistent with a blood clot within otherwise angiographically normal-looking intracranial arteries.
    2) Evidence of complete recanalization of the previously occluded artery.
    3) Presence of multiple acute infarctions that have occurred closely related in time without detectable abnormality in the relevant vessels.
  - ✧ Other cryptogenic strokes
    1) Those not fulfilling the criteria for cryptogenic embolism.
  - ✧ Incomplete evaluation
    1) The absence of diagnostic tests that would have been essential to uncover the underlying cause.
- Unclassified
  - ✧ The presence of >1 evident mechanism; either there is probable evidence for each, or no single cause can be reliably established.

Summary of protocol amendments

Protocol changes version 1.0 (20 Jun, 2020) to 2.0 (9 March, 2022)
Highlight in yellow was the new added content

**Protocol Version 1.0**	**Protocol Version 2.0**
**Abstract (Statistical analysis):** An interim analysis is scheduled to be performed after approximately 50% of patients complete the visit on Day 90. The purpose of interim analysis is to reestimate the sample size based on primary efficacy indexes. It is conducted by the Independent Data Monitoring Committee (IDMC) and whether the sample size should be increased or remain unchanged is recommended according to the regulations of the IDMC. The sample size may be increased to 1.5 times the scheduled sample size at most, and won't be reduced.	**Abstract (Statistical analysis):** An interim analysis is scheduled to be performed after approximately 50% of patients complete the visit on Day 90. The purpose of interim analysis is to reestimate the sample size based on primary efficacy indexes. It is conducted by the Independent Data Monitoring Committee (IDMC) and whether the sample size should be increased or remain unchanged is recommended according to the regulations of the IDMC. The sample size may be increased to 1.5 times the scheduled sample size at most, and won't be reduced. If the sample size is adjusted, the method proposed by Cui et al. 1999 (hereinafter referred to as the CHW method) will be used to control type I errors in the final analysis.
**Common unblinding regulations:** The one-time unblinding method is adopted in this trial. After blind verification, the data will be locked and	**Common unblinding regulations:** The one-time unblinding method is adopted in this trial. After blind verification, the data will be locked and

unblinded, and the corresponding group of each subject will be defined.	unblinded, and the corresponding group of each subject will be defined. For interim analysis, the unblinded data will be centrally reviewed by IDMC members according to IDMC regulations. Statistical analysis of the data will be completed by third-party unblinded statisticians independent of the Sponsor, so as to keep blinding during the trial.
**Interim analysis:** The sample size re-estimation will be estimated according to conditional power calculations as indicated by the promising zone method. According to the IDMC regulations, it is recommended to increase or maintain the sample size, The increase in sample size can be up to 1.5 times the original planned sample size, and the sample size will not decrease.	**Interim analysis:** The sample size re-estimation will be estimated according to conditional power calculations as indicated by the promising zone method. To manage the overall type I error=0.025 (one-sided), the significance level will be adjusted. If the sample size was adjusted, the final statistical analysis of the primary efficacy outcome and the correction of p value will be performed based on the CHW approach using a weighted statistic. The IDMC will conduct interim analysis.

Summary of protocol amendments

Protocol changes version 2.0 (9 March, 2022) to 3.0 (22 Jun, 2022)
Highlight in yellow was the new added content

Protocol Version 2.0	Protocol Version 3.0
**Main estimated target:** NA	**Main estimated target:** The main clinical concerns of this study: Based on treatment policy strategy, the efficacy of Y-2 Sublingual Tablets versus placebo in patients with acute ischemic stroke was assessed by the proportion of participants with an mRS score ≤1 at Day 90 after treatment, regardless of whether the participants had delayed or discontinued the administration, or used protocol-allowed concomitant medications (as specified in Section 5.8.1) due to adverse events.<br>The main estimated target consists of the following attributes:<br>• Population: adult patients with acute ischemic stroke. The study population in this trial is determined by the inclusion and exclusion criteria defined in Section 4;<br>• Treatment: Y-2 Sublingual Tablets 36 mg (containing 30 mg

	of edaravone and 6 mg of dexborneol) or Y-2 placebo control (containing 60 μg of dexborneol to simulate the cool taste of sublingual administration of the investigational product) BID for 14 consecutive days, 28 times in total;<br>• Endpoint: treatment compliance status derived from the mRS score at Day 90 after treatment (compliance: mRS score ≤ 1);<br>• Handling of other concomitant events:<br>○ Use of concomitant medications/therapies during the treatment period but continued with investigational product treatment and follow-up after investigator assessment; or delay, interruption of administration, or discontinuation of the treatment due to AEs, but no early withdrawal from the study; or mRS scores at required time points could only be obtained through remote visit due to the impact of COVID-19: Use of treatment policy strategy (regardless of the occurrence of such events);<br>○ No mRS score after initiation of the treatment for any reason; or early withdrawal from protocol-specified

	follow-up due to AEs: use of a composite strategy (occurrence of such events was counted as a substandard mRS score);<br>○ Early discontinuation of the treatment or withdrawal from the study for reasons other than those mentioned above: use of the while-on-treatment strategy (i.e., mRS compliance status was evaluated based on the mRS score closest to the withdrawal from the study).<br>• Population-level summary: The proportion of patients whose mRS score ≤1 in the Y-2 Sublingual Tablets treatment group and the placebo treatment group was summarized based on the mRS score at Day 90 after the treatment for 14 days in line with the established treatment protocol, and the rate difference between the two groups was calculated.
**Efficacy evaluation:**<br>✧ For the overall outcome scale, it is assessed by the Modified Rankin Scale (mRS), which measures the global disability. See Appendix 2 for details.	**Efficacy evaluation:**<br>✧ For the overall outcome scale, it is assessed by the Modified Rankin Scale (mRS), which measures the global disability. See Appendix 2 for details.

✧ Neurological Disability Score (NDS) scale. See Appendix 3 for the NIH Stroke Scale (NIHSS).<br>✧ SSS TOAST classification	✧ Neurological Disability Score (NDS) scale. See Appendix 3 for the NIH Stroke Scale (NIHSS).
**Efficacy analysis:** All the analyses were performed in the intention-to-treat population. Group difference in the primary efficacy outcome was examined using chi-square test or Fisher exact test, and the corresponding 95% confidence intervals (CIs) of the difference between proportions were estimated based on the normal-approximation. Odds ratios (ORs) with 95% CIs were calculated using logistic regression.	**Efficacy analysis:** All the analyses were performed in the intention-to-treat population. Group difference in the primary efficacy outcome was examined using chi-square test or Fisher exact test, and the corresponding 95% confidence intervals (CIs) of the difference between proportions were estimated based on the normal-approximation. Odds ratios (ORs) with 95% CIs were calculated using logistic regression. Missing data on the primary outcome was imputed with treatment policy strategy, composite variable strategies, and while on treatment strategies accounting for intercurrent events, as follows:<br>• Treatment policy strategy<br>For the following concomitant events: After the subject is successfully randomized and starts to receive the treatment, concomitant medication/treatment is used during treatment (see Section 5.8) and the investigator does not discontinue the treatment or let the

	subject withdraw from the study early based on the actual situation; or the subject does not withdraw from the study early despite treatment delay, interruption or discontinuation due to AEs; the subject's mRS score can only be obtained remotely due to the pandemic, The primary efficacy outcomes will be treated by the treatment policy strategy, that is, regardless of whether the concomitant events occur. Whether the mRS reaches the standard will be assessed according to the measured mRS score collected (the mRS score of remote visit is considered valid).<br>• Composite strategy<br>For the following concomitant events: Randomized subjects, for whatever reason, do not have an mRS score after the start of the treatment;; or the main reason for the subject's early withdrawal from follow-up prescribed by the protocol is AE, the primary efficacy indexes will be treated by the composite strategy, i.e., the occurrence of such events is considered as having a mRS score of 6 at the end of follow-up (Day 90 after treatment).<br>• While on treatment strategy

	When a subject discontinues the treatment or withdraws from the study early for reasons other than those mentioned in the composite index strategy, the primary efficacy indexes will be treated by the while on treatment strategy, that is, using the most recent mRS score before the withdrawal from the study as the basis for determining whether the mRS meets the target.
**Safety analysis:** Adverse events, adverse events during the study period, major adverse events, unexpected adverse events, adverse event of special interest, serious adverse events, adverse events related to study drugs and serious adverse events related to study drugs are classified by system organ class and preferred term.	**Safety analysis:** Adverse events, adverse events during the study period since the first dose of the drug, major adverse events, unexpected adverse events, adverse event of special interest, serious adverse events, adverse events related to study drugs and serious adverse events related to study drugs are classified by system organ class and preferred term.

Statistical Analysis Plan

Protocol Name:	Phase III Clinical Trial of Sublingual Edaravone Dexborneol in the Treatment of Acute Ischemic Stroke - a Multicenter, Randomized, Double-blind, Parallel, Placebo-controlled Phase III Clinical Trial
Investigational Product:	Sublingual edaravone dexborneol (also named Y-2 sublingual tablet as below)
Sponsor:	Jiangsu Simcere Pharmaceutical Co., Ltd.
Author:	Yuanping Yue
Version/Date:	V1.0/25JUL2022

Contents

List of Abbreviations **4**

1 Introduction **5**

2 Study Introduction **5**

2.1 Study objectives 5

2.2 Study Design 5

2.3 Study Endpoints 5

2.4 Primary Estimated Target 6

2.5 Sample Size Determination 8

2.6 Randomization and Blinding 8

2.7 Modification to the statistical analysis in the protocol 9

3 Analysis Dataset **9**

4 Statistical Analysis **10**

4.1 General Principles 10

4.1.1 Significant Level 11

4.1.2 Estimation of Missing Values 11

4.1.3 Data Arrangement 11

4.2 Study Population 13

4.2.1 Subject disposition 13

4.2.2 Protocol Violation/Deviation 13

4.2.3 Demographic Characteristics and Disease Baseline Conditions 13

4.2.4 Medical History 14

4.3 Efficacy analysis 14

4.3.1 Primary Efficacy Endpoint and Analyses 14

4.3.2 Secondary Efficacy Endpoints 17

4.4 Safety analysis 18
4.4.1 Drug Exposure and Compliance Analysis 18
4.4.2 Adverse Events 18
4.4.3 Severe Adverse Events 19
4.4.4 Vital Signs and Laboratory Test Examination 19
4.5 Interim Analyses 19
4.5.1 Unblinded Sample Size Re-estimation 20

List of Abbreviations

Abbreviations and Terms	Definition
AE	Adverse Events
ANCOVA	Analysis of Covariance
CRF	Case Report Form
ECG	Electrocardiogram
EGFR	Glomerular clearance
FAS	Full Analysis Set
ICH	International Conference on Harmonisation of Technical Requirements for Registration of Pharmaceuticals for Human Use
IDMC	Independent Data Monitoring Committee
ITT	Intent-to-treat analysis
MedDRA	Medical Dictionary for Regulatory Activities Terminology
MRS	Modified Rankin Scale
NIHSS	National Institutes of Health Stroke Scale
OR	Odds ratio
PPS	Per-Protocol Set
SAE	Serious Adverse Events
SAP	Statistical Analysis Plan
SAS	Statistical Analysis Software
SS	Safety Analysis Set
TEAEs	Treatment-Emergent Adverse Events
AE	Adverse Events

1 Introduction

The planned analyses and corresponding statistical methods for Study are provided in this Statistical Analysis Plan (SAP).

2 Study Introduction

2.1 Study objectives

To evaluate the efficacy and safety of Y-2 sublingual tablet in patients with acute ischemic stroke.

2.2 Study Design

This study is a multicenter, randomized, double-blind, parallel, placebo-controlled trial.

The study is divided into three periods: Screening/Baseline Period, Treatment Period, and Follow-up Period.

Screening/Baseline Period: Subjects enter the Screening/Baseline Period after signing the informed consent form.

Treatment period: Screened eligible subjects are randomized to receive Y-2 sublingual tablets (investigational group) or Y-2 placebo (control group) for 14 consecutive days. Performing protocol-required tests and assessing safety during treatment.

Follow-up Period: Subjects who finish treatment enter the follow-up period and are followed up until Day 90 from treatment. Stroke related scale scores are performed at 14, 30, and 90 days from the start of the first study drug use, and adverse events are recorded during follow-up to further assess safety.

2.3 Study Endpoints

- **Primary Efficacy Endpoint**
    - The proportion of patients with mRS score ≤ 1 on Day 90 after randomization.
- **Secondary Efficacy Endpoints**

- mRS score on Day 90 of treatment;
- the proportion of patients achieving a good functional outcome (mRS ≤2) on day 90;
- the change in NIHSS score from baseline to 14 days.
- the proportion of patients achieving an NIHSS score ≤1 on day 14, 30 and 90 days after randomization.

- **Safety Endpoints**

  Safety evaluation include:

  - adverse events within day 90.
  - treatment related adverse events within day 90.
  - changes in vital signs and laboratory data before and after treatment.

According to the protocol, exploratory biomarker studies will also be conducted in parallel in this study, and data collection and analysis will be handled separately and not included in this statistical analysis plan.

2.4 Primary Estimated Target

The primary clinical concern of this study is to assess the efficacy of Y-2 sublingual tablet versus placebo in patients with acute ischemic stroke based on the treatment strategy, whether or not the subject experienced a dose delay, interruption, or use of a protocol-allowed concomitant medication (as defined in Protocol Section 5.8.1) due to an adverse event, in terms of the proportion of subjects with mRS score ≤ 1 at Day 90 after start of treatment.

The primary estimated target attributes are shown in Table 2- 1.

Table 2- 1: Primary Estimated Target Attributes

**Attributes**	**Descriptions**
**Target population**	Adult patients with acute ischemic stroke, the study is limited to all subjects who meet the inclusion and exclusion criteria for the trial and are successfully randomized.
**Treatment measures**	Y-2 sublingual tablets 36 mg (containing edaravone 30 mg and dexborneol 6 mg) or Y-2 placebo (containing 60 micrograms dexborneol to mimic the cool taste when administered like the investigated drug) BID for 14 consecutive

	days for a total of 28 times.<br>Concomitant medications/therapies may be administered during the study (from signing of informed consent through last follow-up) as specified in Section 5.8 of the protocol.
**Study Endpoints**	Treat-to-target status was derived based on the mRS score at day 90 after the start of treatment, i.e., a mRS score ≤ 1 was considered to be treat-to-target.
**Intercurrent Events and Addressing**	• **The following intercurrent events were managed using a treatment policy strategy, i.e., regardless of whether they occurred or not:**<br>o Use of concomitant medication/therapy during the treatment period (regardless of whether concomitant medication/therapy is permitted or prohibited), but continue trial drug treatment and follow-up after investigator 's assessment;<br>o Delayed, interrupted, or discontinued treatment due to an AE, but did not prematurely withdraw from the study;<br>o Because of the impact of the new coronavirus epidemic, the required time point mRS score could only be obtained by remote visits.<br>• **A composite variable strategy was used to address the following intercurrent events, i.e., the occurrence of such events would be considered as suboptimal mRS scores:**<br>o No mRS score after start of treatment regardless of cause;<br>o Premature withdrawal from protocol-specified follow-up due to AEs.<br>• **While-on-treatment strategy, defined as mRS compliance status based on the mRS score closest to the event, was used to handle premature treatment discontinuation or study withdrawal for reasons other than those mentioned above.**
**Population Level Summary Indicators**	The proportion of patients achieving a mRS score of ≤ 1 was summarized for the Y-2 sublingual tablet group and the placebo group based on the mRS score at day 90 after the start of treatment according to the established treatment regimen for 14 days, and the rate difference between the two groups was calculated.

2.5 Sample Size Determination

The sample size for this study was based on the primary efficacy measure, the proportion of subjects with mRS score ≤ 1 at Day 90 of treatment. According to previous clinical studies (see Protocol Reference 7-12), it is estimated that the proportion of subjects with mRS score ≤ 1 on Day 90 of treatment will be 50% in the investigational group and 40% in the control group, taking a two-sided α of 0.05, a power of 80%, and a randomization ratio of 1:1. After calculation, 388 subjects are required in each group. Considering a 15% dropout rate, 457 subjects are planned to be randomized in each group, with a total sample size of 914.

An interim analysis is scheduled to be performed after approximately 50% of patients complete the visit on Day 90. The purpose of interim analysis is to re-estimate the sample size based on primary efficacy indexes. It is conducted by the Independent Data Monitoring Committee (IDMC) and whether the sample size should be increased or remain unchanged is recommended according to the regulations of the IDMC. The sample size may be increased to 1.5 times the scheduled sample size at most, and won't be reduced. If the sample size is adjusted, the method proposed by Cui et al. 1999 (hereinafter referred to as the CHW method) will be used to control type I errors in the final analysis.

Interim analyses and sample size re-estimation were performed by an Independent Data Monitoring Committee (IDMC) as detailed in Section 4.5 of this plan and in the IDMC charter.

2.6 Randomization and Blinding

All eligible subjects will be randomly assigned to the investigational or control group according to the ratio of 1:1. The random number table is generated by the standardized software. The detailed parameter settings are set by the randomization plan.

In this trial, a centralized competitive enrollment randomization method will be used, and subjects will be assigned a "screening number" after signing the informed consent form and before performing study-related tests. Subject screening numbers must not be reused.

After the subjects passed screening, the investigator or designee obtained the information of randomization number and corresponding drug number through the randomization system, and distributed the corresponding study drugs according to the randomization number and drug number.

Randomization will be stratified by:

- Onset time: ≤ 24 hours vs > 24 hours;
- Investigational site.

The color, description, and packaging (including batch number and shelf life) of the investigational drug (Y-2 sublingual tablet) and placebo control drug (Y-2 sublingual tablet placebo) were identical. Blinding process was completed by statisticians unrelated to this clinical trial. After blinding, the blinding codes will be sealed in duplicate and stored in the leading site and the sponsor, respectively.

The trial was unblinded just once. After the last subject completes the 90-day follow-up in the whole trial, after blind verification, the data is locked and unblinding is performed, that is, the corresponding group of each subject is identified. For interim analysis, according to IDMC charter, unblinded data will be reviewed centrally by IDMC members and their statistical analysis will be performed by a third party unblinded statistician independent of the study project team to ensure blinding during the conduct of the trial.

When a serious adverse event suspected to be related to the investigational drug occurs during the trial, the principal investigator at the site should report to the sponsor to decide whether to initiate the emergency unblinding procedure. Once the blind was broken emergently, the case was treated as a dropout and medication was stopped if the blind was broken emergently during treatment.

This double-blind trial was invalidated if the total blind codes were leaked or emergency unblinding exceeded 20% during the study.

2.7 Modification to the statistical analysis in the protocol

Not available.

3 Analysis Dataset

At the end of the trial, after data cleaning, the study project team will review the data collected during the trial and delimit the analysis dataset according to the following rules.

- **Full Analysis Set (FAS)**

According to the basic principle of intention-to-treat (Intention-To-Treatment, ITT), all subjects who were randomized were included in the full analysis set. The primary efficacy evaluation for

this study was based on the full analysis set.

Treatment assignment for the Full Analysis Set was based on randomization.

- **Per-Protocol Set (PPS)**

Includes all subjects who completed protocol-specified therapy or who did not substantially violate the protocol. The exact definition of a serious protocol violation will be finalized at the time of data review and may generally include (but is not limited to) the following:

1) Significant violation of inclusion criteria, affecting the efficacy evaluation;
2) There are treatments that seriously interfere with the efficacy evaluation after inclusion;
3) Significant drug regimen violation, total dose intensity < 80%;
4) Poor compliance seriously affects the efficacy evaluation;
5) Delayed medication for too long during treatment;
6) Lack of evaluation of primary efficacy indicators.

The PPS was the secondary analysis dataset for efficacy evaluation, and treatment assignment was based on randomization.

- **Safety Analysis Set (SS)**

Defined as all subjects who were randomized and received at least one dose of trial drug and had one safety evaluation. The safety evaluation for this study was based on the Safety Analysis Set.

The treatment groups of the safety analysis set was based on the drug actually received. For the control group, data analysis will be performed by the investigational group as long as the subjects have received one dose of the test drug.

4 Statistical Analysis

4.1 General Principles

All statistical analyses for this study will be performed using the statistical analysis software SAS version 9.4 or higher. All data collected in Case Report Form (CRF) will be statistically calculated by treatment groups. For continuous variables, the number of subjects with non-missing data, mean, standard deviation, median, minimum, and maximum were calculated and listed. The number of decimal places for the minimum and maximum values will be

consistent with that recorded in the database. Means, medians, and standard deviations will be rounded to one more decimal place than the raw data recorded in the database. For discrete variables, frequencies and percentages were calculated and presented. Percentages will be presented to one decimal place.

4.1.1 Significant Level

Unless otherwise specified, all efficacy analyses will be statistically tested at a 2-sided significance level $\alpha = 0.05$ to calculate nominal p values, point estimates of efficacy differences, and 2-sided 95% confidence intervals.

4.1.2 Estimation of Missing Values

Missing values were not imputed except for the primary efficacy measure. Missing value imputation methods for the primary efficacy measure are detailed in Text Section 4.3.1.

4.1.3 Data Arrangement

1) **Wrong grouping convention:** Following conventions will be made for the cases with wrong drug used during the trial:
   - FAS: In case of any inconsistency between randomization and drug grouping, randomization shall prevail
   - PPS set: Randomization inconsistent with drug use will be excluded.
   - SS set: Statistical analysis will be performed according to actual drug group when randomization is inconsistent with drug grouping.
2) **Missing dates:** If previous dates are incomplete and affect subsequent date calculations, impute as follows if there is no conflict with other dates:
   - Date associated with adverse event
     - o Day, Month, and Year of event onset date are all missing: do not impute, date is missing;
     - o Missing day and month of AE onset date: If the year of onset date is the same as the year of first dose date, fill in as first dose date; if the year of onset date is not the same as the year of first dose date, fill in as January 1.
     - o Date of AE onset is only missing for day: If the date of AE onset is same as the date of first dose, fill as the date of first dose; if the date of AE onset is different from

month and year, fill as day 1.

- Date of AE end is missing: Date of end is not imputed and will be considered missing.
- Missing day and month of stop date in adverse event: end date imputed as 31 December.
- End date in Adverse Event is only missing for Day: Impute as last day of month.

- Dates associated with concomitant medications
  - Day, Month, and Year of start of concomitant medication are all missing: do not impute, date is missing;
  - Missing day and month of concomitant medication start date: If the year of start date is the same as the year of first medication date, fill in as first medication date; if the year of start date is not the same as the year of first medication date, fill in as January 1.
  - Concomitant medication start date is missing only for Day: If "Month and Year" of start date are the same as "Month and Year" of first medication date, fill in as first medication date; if "Month and Year" of start date are not the same as "Month and Year" of first medication date, fill in as day 1.
  - Date of end of concomitant medication is missing: End date is not imputed and agreed as continuous use.
  - Day and month of concomitant medication end date are missing: end date is imputed as 31 December.
  - Concomitant medication end date is only missing for Day: Impute as last day of month.
  - Date of first dose appointment: Date of start of treatment period

Dates in the data listings were listed as completed on the eCRF.

3) Definition of baseline: The last non-missing data before the first dose is baseline. If a subject took trial medication but did not have a date of first dose, the last non-missing data prior to the date of enrollment was taken as baseline.

4) Change from baseline was defined as: test value at post-treatment follow-up – baseline test value.

5) Data derivation and transformation:

- Age: Year is used as unit, = (date of informed consent – date of birth)/365.25, rounded.
- Time from treatment to onset = time of first dose – time of stroke onset in hours (h) rounded to one decimal place.
- Stroke onset time is defined as the time at which the patient 's symptoms begin, and if it starts during sleep, the time at which the last manifestation is normal should be used as the time of onset. If stroke onset time is completely missing, impute with 0h0m on day of onset; if only minutes are missing, impute with 0m.
- Estimated glomerular filtration rate (eGFR):
- eGFR (ml/min/1.73m^2) = 186 * (Scr/88.4)$^{-1.154}$ * (age)$^{-0.203}$ * 0.742 (if female) * 1.233

  Where Scr is serum creatinine (umol/L), age is age, and female is female.

  Renal function: normal renal function (baseline eGFR ≥ 90), mild renal impairment (6 0 ≤ baseline eGFR < 90), and moderate to severe renal impairment (baseline e GFR < 60).

4.2 Study Population

4.2.1 Subject disposition

Descriptive statistics will be performed for subjects screened, randomized, treated, dropped out and reasons for dropout by treatment groups, and the number and percentage of subjects in each category will be calculated. If necessary, summary statistics by site will be provided.

Descriptive statistics will be performed on the subjects' enrollment in each analysis dataset and reasons for exclusion, and the number of cases and percentage will be calculated.

Screening, randomization, and treatment of subjects in the trial were tabulated.

4.2.2 Protocol Violation/Deviation

The number and percentage of protocol violations/deviations will be summarized by category for each treatment groups. Major protocol violations/deviations will be listed by subject.

4.2.3 Demographic Characteristics and Disease Baseline Conditions

Demographic characteristics and disease baseline conditions will be summarized by treatment groups on the FAS. Number of non-missing values, mean, standard deviation, median, minimum,

and maximum were calculated for continuous variables. Discrete variables were calculated as frequency and percentage.

Demographic characteristics included age, sex, height, weight, body mass index. Vital signs including supine systolic blood pressure, and diastolic blood pressure, will also be listed and summarized in each treatment group. Baseline disease conditions included mRS score, NIHSS score, stroke history, stroke classification, and renal function.

4.2.4 Medical History

Medical history will be statistically analyzed by treatment groups based on FAS set.

Medical history was coded using the applicable version of MedDRA at the time of data analysis and summarized by system organ class and preferred term by treatment groups.

Medical history will be detailed in listings.

4.3 Efficacy analysis

4.3.1 Primary Efficacy Endpoint and Analyses

The primary efficacy measure in this trial was the proportion of subjects with mRS score ≤ 1 at Day 90 of treatment. The main estimated target attributes under the estimated target framework are presented in Section 2.4, and the different coping strategies proposed for the intercurrent events are as follows:

- **Treatment Policy Strategy**

  When subjects participating in the trial were successfully randomized and treated, if concomitant medication/therapy was used during the treatment period (see Section 5.8 of the protocol), the investigator did not terminate the treatment or allow them to withdraw prematurely from the study according to the actual situation; or the subject did not withdraw prematurely from the study despite dose delay, interruption, or termination due to an AE; or because of a new coronavirus outbreak, the subject mRS score could only be obtained remotely. For such intercurrent events, the primary efficacy measure will be handled by the treatment policy strategy, i.e., the mRS achievement or failure will be discriminated according to the collected measured mRS score (remote visit mRS score will be considered valid) regardless of whether the intercurrent event occurred.

- **Composite Variable Strategy**

When a randomized subject had no mRS score after starting treatment for any reason; or the subject prematurely withdrew from protocol-specified follow-up because of an AE. Such intercurrent events will be handled for the primary efficacy measure using a composite variable strategy, i.e., the occurrence of such events will be considered as having a mRS score > 1 at the end of follow-up (Day 90 post-treatment).

- **While-on-Treatment Strategy**

When subjects participating in the trial prematurely discontinue treatment or withdraw from the study for reasons other than those mentioned in the above strategy, the on-treatment strategy will be used to deal with the primary efficacy indicators, that is, the mRS score closest to the time of withdrawal will be used as the basis for judging whether the mRS is up to standard.

Missing values for the primary efficacy measure will be handled accordingly by different handling strategies for the above intercurrent events, and subjects who prematurely discontinue treatment or withdraw from follow-up due to emergency unblinding resulting in missing mRS scores on Day 90 will be handled as one of the coping strategies for the above intercurrent events for reasons documented in the CRF.

Primary efficacy endpoint analysis will be performed on FAS and PPS separately.

Primary Estimated Method:

Descriptive statistics were performed on the proportion of subjects with mRS score ≤ 1 at Day 90 by treatment groups, the number and percentage of subjects with mRS score ≤ 1 at Day 90 of treatment were calculated, and the rate difference between the two groups (investigational group - control group) and 95% confidence interval were calculated based on the normal-approximation. Odds ratios (ORs) with 95% CIs were calculated using logistic regression.

Considering that the interim analysis (done by IDMC and independent statistical team) will be performed in this study after 50% of subjects have completed the 90-day visit, and an unblinded sample size re-estimation will be performed, and in case of sample size adjustment, p values will be calculated based on the statistics obtained after correction by the CHW method, and ORs and 95% confidence intervals will be further estimated. If the lower limit of the 95% confidence interval of the OR was > 1, the investigational group was considered to have better efficacy than the control group. The main analysis of the primary efficacy indicators is mainly based on the FAS results, and the inconsistent FAS results and PPS results need to be further explored.

- Sensitivity Analysis

Sensitivity analyses were performed on the FAS only.

Sensitivity Analysis 1:

Missing data on the primary efficacy outcome was imputed with best case analysis (assume any subject missing 90-day outcome to be mRS score ≤1). Logistic regression analysis was performed to calculate the OR and 95% confidence interval.

Sensitivity Analysis 2:

Missing data on the primary efficacy outcome was imputed with Worst case analysis (assume any subject missing 90-day outcome to be mRS score >1). Logistic regression analysis was performed to calculate the OR and 95% confidence interval.

Sensitivity Analysis 3:

Missing data on the primary efficacy outcome was imputed with LOCF combined with best case analysis (assume any subject missing 90-day outcome after LOCF to be mRS score ≤1). Logistic regression analysis was performed to calculate the OR and 95% confidence interval.

Sensitivity Analysis 4:

Missing data on the primary efficacy outcome was imputed with LOCF combined with worst case analysis (assume any subject missing 90-day outcome after LOCF to be mRS score >1). Logistic regression analysis was performed to calculate the OR and 95% confidence interval.

Sensitivity Analysis 5:

Logistic regression analysis was performed to calculate the OR and 95% confidence interval between the investigational and control groups using treatment as the analysis variable and baseline time from actual treatment to onset (≤ 24 hours, > 24 hours), and study site as covariates.

- Subgroup analysis

The primary efficacy endpoint will be analyzed in subgroups according to the following factors:

  - Age (≤ 65, > 65)
  - Gender (male, female)
  - Actual treatment to Onset Category 1 (≤ 24 hours, > 24 hours)

- Premorbid mRS score (0, 1)
- Pre-treatment NIHSS total score classification (≤ 7, > 7)
- History of stroke (Yes, No)
- History of hypertension (Yes, No)
- History of hyperlipidemia (Yes, No)
- History of diabetes (Yes, No)
- History of heart disease (yes, no)
- Stroke etiology classification
- Renal function class (normal, mild impairment and moderate to severe impairment as assessed by baseline eGFR)

4.3.2 Secondary Efficacy Endpoints

Secondary efficacy endpoints were analyzed on the FAS and PPS without imputation of missing values.

- mRS score on Day 90 of treatment

Descriptive statistics were performed by treatment groups on mRS scores at Day 90 of treatment to calculate the number of non-missing subjects, mean, standard deviation, median, minimum, and maximum, and at the same time, the number and percentage of subjects with different mRS scores (including missing values) at Day 90 of treatment were calculated. an ordinal logistic regression analysis was performed, with the results presented as common OR and 95% CI, where a common OR in favor of Y-2 sublingual tablet was >1.0

- Proportion of subjects with mRS score ≤ 2 at Day 90 of treatment

Descriptive statistics were performed on the number and percentage of subjects with mRS score ≤ 2 at Day 90 of treatment as previously described by treatment groups. Comparisons were made between treatment groups using the same logistic regression model as for the primary analysis.

- NIHSS score change from baseline at Day 14 of treatment

Observed values and changes from baseline in NIHSS score at each visit will be summarized descriptively by treatment groups and the number of non-missing subjects, mean, standard

deviation, median, minimum, and maximum will be calculated. Difference in NIHSS score from baseline to Day 14 was be calculated the means with 95% CIs were calculated for each group, and the mean differences with 95% CI between the groups were estimated by generalized linear regression.

- The proportion of NIHSS score of 0-1 at 14, 30, and 90 days of treatment;

The number and percentage of subjects with NIHSS scores of 0 to 1 at 14, 30, and 90 days of treatment will be calculated by treatment groups. Comparisons were made between treatment groups using the same logistic regression model as for the primary analysis.

- Other Efficacy Measures

Not applicable.

4.4 Safety analysis

All safety analyses were based on SS unless otherwise specified.

4.4.1 Drug Exposure and Compliance Analysis

Days on medication and total doses taken during the treatment period will be summarized by treatment groups, and the number of non-missing data, mean, standard deviation, median, minimum, and maximum will be calculated.

Medication compliance was calculated according to the following rules:

Medication compliance = (actual dose/planned dose) * 100%

Frequency and percentage of medication compliance will be calculated as < 80%, 80% to 100%, and > 100% in addition to the number, mean, standard deviation, median, minimum, and maximum calculated for medication compliance by continuous variables.

Summaries of drug exposure will also be summarized separately by age (≤ 65, > 65) and renal function class (normal, mildly impaired, and moderately to severely impaired).

Subjects will also be listed for study drug intake during the trial.

4.4.2 Adverse Events

Adverse event data will be coded according to the current version of MedDRA. Treatment Emergent Adverse Event (TEAE) will be used in the analysis, while adverse events that start prior to the first dose of study medication (pre-treatment) will not be involved into the safety

analysis. Adverse events on study medication (during treatment) and after the last dose of study medication (post-treatment) will be analyzed.

The differences in the proportions of patients with AE between treatment groups and their corresponding 95% CIs will be estimated based on Newcombe–Wilson. The ORs and 95% CIs will be estimated with the logistic regression. Chi-squared test or Fisher's Exact test will be used to compare the number of each AE combination according to system organ class and preferred term between treatment groups.

4.4.3 Severe Adverse Events

The differences in the proportions of patients with SAE between treatment groups and their corresponding 95% CIs will be estimated based on Newcombe–Wilson. The ORs and 95% CIs will be estimated with the logistic regression. Chi-squared test or Fisher's Exact test will be used to compare the number of each SAE combination according to system organ class and preferred term between treatment groups.

4.4.4 Vital Signs and Laboratory Test Examination

Within each treatment group, the number and percentage of subjects with elevated in vital signs from baseline to day 14 after randomization will be described and Fisher's Exact test will be used to compare the difference between treatment groups. The baseline measurements and changes after treatment of blood routine and bloodbiochemistry will be described as "abnormal and clinically significant" will be determined by the researchers. Within each treatment group, the number and percentage of subjects with an "abnormal and clinically significant" value will be summarized and Fisher's Exact test will be used to compare the difference between treatment groups.

4.5 Interim Analyses

The study planned an interim analysis after approximately 50% of participants, i.e. 456 participants, completed the 90 day visit, with the primary endpoint of unblinded sample size re-estimation based on the observed proportion of participants with mRS score ≤ 1 at Day 90 of treatment (primary efficacy endpoint). At the same time, the basic information and some safety

data of the enrolled subjects will also be initially reviewed. Interim analyses will be conducted by the IDMC and an independent statistical team supporting the IDMC, as specified in the IDMC charter, unblinded data will be kept confidential to the sponsor, and recommendations resulting from the interim data review meeting will be communicated to the sponsor in addition to aid project decision-making.

The IDMC meeting for this study was held on 29 April 2022 and the decision was made not to increase the sample size.

4.5.1 Unblinded Sample Size Re-estimation

Sample size re-estimation for interim analysis is performed using promising zone based on conditional power method, setting sample size re-estimation to be performed when 50% of subjects provide primary efficacy indicator data, while limiting the adjusted maximum sample size to only 1.5 times sample size of the initial plan without sample size reduction plan. Meanwhile, it is necessary to ensure that the power of final analysis is maintained at least 80%. According to Mehta CR , Pocock SJ 2011(Cui L, Hung HM, Wang SJ. Modification of sample size in group sequential clinical trials. Biometrics 1999; 55:853--857), to ensure that the Type I error probability (α level) at the final analysis of the trial is not inflated, the conditional power *CP* (z_1, n_2) was calculated based on Equation (1), And divided into three intervals *CP* $(z_1, n_2) <$ 0.41, $0.41 \leq$ *CP* $(z_1, n_2) < 0.8$ and *CP* $(z_1, n_2) \geq 0.8$ for sample size re-estimation.

$$CP_{(z1,n2)} = \mathrm{P}\left(z_2 \geq z_{1-\alpha/2} \middle| z_1\right) = 1 - \emptyset\left(\frac{z_{1-\alpha/2}\sqrt{N} - z_1\sqrt{n1}}{\sqrt{n2}} - \frac{z_1\sqrt{n2}}{\sqrt{n1}}\right) \qquad (1)$$

In equation (1) z_1 represents the *z* statistic at the interim analysis and z_2 represents the *z* statistic at the final analysis. $Z_{1-\alpha/2}$ represents the margin corresponding to the (1-α/2) percentile under the standard normal distribution, where α = 0.05. Φ(x) represents the probability that the observed value ≤ x under the standard normal distribution. N represents the initial planned sample size, n1 represents the sample size at the interim analysis, n2 represents the sample size after the interim analysis in the initial plan, where $N = n1 + n2$.

Interim analyses of the primary efficacy measure will be performed using the same logistic regression model (see Text Section 4.3.1) as the primary analysis of the primary efficacy measure according to Glenn A. Walker, Jack Shostak 2010 (Mehta CR, Pocock SJ. Statistics in Medicine 2011, 30:3267-3284. Adaptive Increase in Sample Size when Interim Results are Promising: A Practical Guide with Examples), The *Z* statistic Z_1 at the interim analysis was calculated from the treatment effect coefficient β_1 in the logistic regression model and the corresponding standard error $SE_{\beta 1}$, That is $Z_1 = \beta_1/SE_{\beta 1}$.

Substituting Z_1 calculated from the logistic regression model into equation (1), the conditional power $CP(z_1, n_2)$ can be calculated, depending on its interval, with subsequent sample size adjustments according to Table 4-1.

Table 4- 1: Sample Size Adjustment Rules for Different Conditional Power

Interval Name	Decision Rule	Sample Size Adjustment Instructions
Unfavorable interval	$CP_{(z1,n2)} < 0.41$	No sample size adjustment was performed and testing continued to the initial planned sample size.
Expected interval	$0.41 \le CP_{(z1,n2)} < 0.8$	Sample size re-estimation was performed according to equation (2), and if the final total sample size exceeded 1.5 times the initial planned sample size, the final sample size was adjusted to 1.5 times the initial planned sample size.
Favorable interval	$CP_{(z1,n2)} \ge 0.8$	No sample size adjustment was performed and testing continued to the initial planned sample size.

When the conditional power falls within the expected interval, the sample size after analysis in the trial period is calculated according to formula (2), in which β is 0.2, to ensure that the adjusted sample size can still meet the condition of 80% power.

$$n2^* = \left(\frac{n1}{{z_1}^2}\right)\left(\frac{z_{1-\frac{\alpha}{2}}\sqrt{N} - z_1\sqrt{n1}}{\sqrt{N-n1}} + z_{1-\beta}\right)^2 \qquad (2)$$

In Equation (2), $n2^*$ represents the sample size after interim analysis, recorded as $N^* = n1 + n2^*$, If $N^* \ge 1.5*N$, take $n2^* = 1.5*N - n1$, Thus, the final total sample size was 1.5 times the initial planned total sample size, which was limited to a maximum possible total sample size of 1370 considering a 1:1 randomization ratio for this study. In case of sample size adjustment, the statistics will be corrected for statistical inference using the CHW method (see Section 4 .3.1) at the final analysis.

SUMMARY OF DELIBERATIONS BY THE BAST DSMB

The First Independent Data Monitoring Committee (IDMC)

Meeting Minutes

Meeting Date: 2021-9-22

Meeting Time: 10:00 – 12:00

Attendees:

IDMC Members: Jielai Xia, Zhihong Zhao, Xiaoshan Wang

Trial Team Representatives: Qin Huang, Wei Wang

Meeting Location: Video conference

Meeting Minutes by: Wei Wang

1 Trial Overview

**Protocol Name:**	Phase III Clinical Trial of Y-2 Sublingual Tablet in the Treatment of Acute Ischemic Stroke - a Multicenter, Randomized, Double-blind, Parallel, Placebo-controlled Phase III Clinical Trial
**Protocol No.:**	SIM1911-01-Y-2-301
**Investigational Product:**	Y-2 sublingual tablet
**Sponsor:**	Simcere Pharmaceutical Co., Ltd.<br>Yantai YenePharma Co., Ltd.

2 Discussion

**Recruitment Status:**	209 patients enrolled out of the target 914 from 32 participating sites.
**Participant Characteristics:**	Demographics: Diverse patient population, representative of the intended study population.<br>Baseline Characteristics: Balanced distribution of stroke severity, comorbidities, and demographic factors.<br>Trial Progress: Enrollment is on track; challenges related to site-specific variations are being addressed through ongoing communication and training initiatives.
**Safety Data**	No major safety concerns identified; ongoing monitoring and proactive safety measures are in place.<br>Adherence to the study protocol is robust, ensuring data reliability.
**Data Quality**	High data quality maintained through centralized monitoring, regular site visits, and rigorous data management procedures.

3 Decisions Made

**Recruitment Plan :**	Outreach efforts have been effective, with ongoing strategies such as site-specific training sessions and patient engagement initiatives.
**Follow-up Plan :**	Good patient retention; exploratory analyses indicate high compliance with follow-up procedures

4 Future Plans

Next DSMB Meeting Plan: Date on which 50% of the participants completed the follow-up.

The Second Independent Data Monitoring Committee (IDMC)

Meeting Minutes

Meeting Date: 2022-4-29

Meeting Time: 16:00 – 18:00

Attendees:

IDMC Members: Jielai Xia, Zhihong Zhao, Xiaoshan Wang

Trial Team Representatives: Qin Huang, Wenjie Song, Wei Wang

Meeting Location: Video conference

Meeting Minutes by: Wei Wang

1 Trial Overview

**Protocol Name:**	Phase III Clinical Trial of Y-2 Sublingual Tablet in the Treatment of Acute Ischemic Stroke - a Multicenter, Randomized, Double-blind, Parallel, Placebo-controlled Phase III Clinical Trial
**Protocol No.:**	SIM1911-01-Y-2-301
**Investigational Product:**	Y-2 sublingual tablet
**Sponsor:**	Simcere Pharmaceutical Co., Ltd.<br>Yantai YenePharma Co., Ltd.

2 Discussion

**Recruitment Status:**	456 patients enrolled out of the target 914 from 39 participating sites.
**Safety Data**	Subject 020002 was diagnosed with meningitis/brain glioma during the screening phase (prior to initial drug administration) based on the MRI findings. However, clinical confirmation is currently pending. Further follow-up is required to ascertain the diagnosis for this subject.<br><br>It is recommended to reassess the degree of hepatic impairment and, following standardized principles to categorize adverse events uniformly. It is suggested to designate mild hepatic impairment as "Hepatic Function Impairment."<br><br>Three subjects were diagnosed with cerebrovascular stenosis based on CT/MRI conduct during the screening phase. This condition was attributed to medical history and was judged as unrelated to the investigational drug.

3 Decisions Made

**Follow-up Plan :**	Based on the existing analysis results, there is no need to increase the sample size. The trial will continue as planned.

4 Future Plans

Next DSMB Meeting Plan: Date on 100% of the participants completed the follow-up.

The Third Independent Data Monitoring Committee (IDMC)

Meeting Minutes

Meeting Date: 2022-12-25

Meeting Time: 10:00 – 12:00

Attendees:

IDMC Members: Jielai Xia, Zhihong Zhao, Xiaoshan Wang

Trial Team Representatives: Danny Chen, Wei Wang

Meeting Location: Video conference

Meeting Minutes by: Wei Wang

1 Trial Overview

**Protocol Name:**	Phase III Clinical Trial of Y-2 Sublingual Tablet in the Treatment of Acute Ischemic Stroke - a Multicenter, Randomized, Double-blind, Parallel, Placebo-controlled Phase III Clinical Trial
**Protocol No.:**	SIM1911-01-Y-2-301
**Investigational Product:**	Y-2 sublingual tablet
**Sponsor:**	Simcere Pharmaceutical Co., Ltd.<br>Yantai YenePharma Co., Ltd.

2 Discussion

**Recruitment Status:**	914patients enrolled out of the target 914 from 39 participating sites. All the subjects have finished the follow-up.
**Trial Progress Overview:**	Brief presentation on overall trial progress, including enrollment status, participant demographics, and any notable developments.<br><br>Brief overview of quality assurance measures implemented throughout the trial.<br><br>Summary of monitoring activities to ensure protocol adherence and data reliability
**Safety Review:**	Safety data for both treatment and control groups were presented.<br>No major safety concerns reported.<br><br>Discussion on any adverse events and their resolution.
**Protocol Adherence:**	Confirmation that the trial team has adhered to the established protocol.

	Discussion on any deviations and the corresponding corrective actions taken.

3 Decisions Made

Agree to lock the database.
