# Supplementary material for: Sublingual Edaravone Dexborneol for the Treatment of Acute Ischemic Stroke: The TASTE-SL Randomized Clinical Trial
Source: JAMA Neurol. 2024 Feb 19;81(4):319–26. doi: 10.1001/jamaneurol.2023.5716 (PMC10877503; doi:10.1001/jamaneurol.2023.5716)
Supplement: Supplement 2. — eAppendix 1. Committees and Coordinating Centers in the TASTE-SL Trial eAppendix 2. Clinical Sites in Order of Participants Enrolled eTable 1. Inclusion and Exclusion Criteria eTable 2. Concomitant Medication Use Within 90 Days eTable 3. Sensitivity Analyses for the Primary Analysis eTable 4. Efficacy and Safety Outcomes in the Per-Population Analysis eTable 5. Number of Patients With Adverse Events (by System Organ Class ) During 90 Days of Follow-Up eTable 6. Number of Patients With Severe Adverse Events (by System Organ Class ) During 90 Days of Follow-Up eTable 7. Number of Patients With Treatment Related Adverse Events During 90 Days of Follow-Up eTable 8. Number of Patients With Clinically Significant Changes in Vital Signs and Laboratory Data on Day 90 of Follow-Up eTable 9. Laboratory Data on Day 90 After Randomization eTable 10. Laboratory Data on Day 30 After Randomization eTable 11. Laboratory Data on Day 14 After Randomization eTable 12. Laboratory Data on Day 7 After Randomization [file jamaneurol-e235716-s002.pdf]

Supplementary Online Content

Fu Y, Wang A, Tang R, et al. Sublingual edaravone dexborneol for the treatment of acute ischemic stroke: the TASTE-SL randomized clinical trial. *JAMA Neurol*. Published online February 19, 2024. doi:10.1001/jamaneurol.2023.5716

eAppendix 1. Committees and Coordinating Centers in the TASTE-SL Trial

eAppendix 2. Clinical Sites in Order of Participants Enrolled

eTable 1. Inclusion and Exclusion Criteria

eTable 2. Concomitant Medication Use Within 90 Days

eTable 3. Sensitivity Analyses for the Primary Analysis

eTable 4. Efficacy and Safety Outcomes in the Per-Population Analysis

eTable 5. Number of Patients With Adverse Events (by System Organ Class) During 90 Days of Follow-Up

eTable 6. Number of Patients With Severe Adverse Events (by System Organ Class) During 90 Days of Follow-Up

eTable 7. Number of Patients With Treatment Related Adverse Events During 90 Days of Follow-Up

eTable 8. Number of Patients With Clinically Significant Changes in Vital Signs and Laboratory Data on Day 90 of Follow-Up

eTable 9. Laboratory Data on Day 90 After Randomization

eTable 10. Laboratory Data on Day 30 After Randomization

eTable 11. Laboratory Data on Day 14 After Randomization

eTable 12. Laboratory Data on Day 7 After Randomization

This supplementary material has been provided by the authors to give readers additional information about their work.

© 2024 Fu Y et al. *JAMA Neurology.*

eAppendix 1. Committees and Coordinating Centers in the TASTE-SL Trial

Listing of Committees and Coordinating Centers in the TASTE-SL Trial

Steering Committee: Dongsheng Fan, Yu Fu, Renhong Tang, Jinsheng Ren, Shibao Yang

Independent Data Monitoring Committee: Jielai Xia (Department of Health Statistics, Air Force Medical University of PLA), Zhihong Zhao (Department of Neurology, Henan Provincial People's Hospital), Xiaoshan Wang (Department of Neurology, Henan Provincial People's Hospital)

Independent Statistician: Donghua Lou (Department of Biostatistics, School of Public Health, Nanjing Medical University), Yuanping Yue (Nanjing Yike PowerData Medical Technology Co. Ltd)

© 2024 Fu Y et al. *JAMA Neurology.*

eAppendix 2. Clinical Sites in Order of Participants Enrolled

Clinical Sites (Principal Investigator) In Order of Participants Enrolled

Clinical sites	Principal investigators
Harrision International Peace Hospital	Yan Wei
Daqing Oilfied General Hospital	Xueshuang Dong
Nanshi Hospital of Nanyang	Yun Ling
Pingxiang People's Hospital	Fei Yi
The First Affiliated Hospital of Nanyang Medical College	Qian Deng
Liaocheng People's Hospital	Cunju Guo
The First People's Hospital of Shenyang	Yi Sui
Mei He Kou Central Hospital	Shugen Han
Hainan General Hospital	Guoqiang Wen
Xuzhou Central Hospital	Chuanling Li
Cangzhou Central Hospital	Aiqin Dong
The First Hospital of Jilin University	Xin Sun
Taizhou First People's Hospital	Zhimin Wang
Anqing Municipal Hospital	Xueying Shi
The First Affiliated Hospital Baotou Medical College	Bo Liu
Cangzhou People's Hospital	Huagang Wang
Shanghai Pudong New Area People's Hospital	Xioahui Zhao
The First Affiliated Hospital of University of South China	Heng Wu
Zhongda Hospital Southeast University	Fuling Yan
General Hospital of Northern Theater Command	HuishengChen
Tianjin Huanhu Hospital	Yong Ji
Liuzhou Worker's Hospital	Hong Yang
Peking University Third Hospital	Dongsheng Fan
Linyi People's Hospital	Hongxing Han
People's Hospital of Deyang City	Chun Wang
The First Affiliated Hospital of Wenzhou Medical University	Jincai He
Baotou City Central Hospital	Baojun Wang
The Second Affiliated Hospital of Nanjing Medical University	Jin Wu
Xianyang Hospital of Yan'an University	Li Ji
Jinan Central Hospital	Hong Bian
The Second People's Hospital of Huai'an	Liandong Zhao
Ningbo First Hospital	Jianhong Yang
Taizhou Hospital of Zhejiang Province	Shaofa Ke

© 2024 Fu Y et al. *JAMA Neurology.*

eTable 1. Inclusion and Exclusion Criteria

Inclusion Criteria

- Age ≥ 18 years old and ≤ 80 years old, regardless of gender;
- After the onset of the disease, the National Institutes of Stroke Scale score: 6 ≤ NIHSS ≤ 20, and the sum of the fifth upper limb score and the sixth lower limb score was ≥2;
- The onset time is within 48 hours (including 48 hours);
- Patients diagnosed as ischemic stroke according to "key points for diagnosis of all kinds of major cerebrovascular diseases in China 2019", with good prognosis after the first attack or the last attack (MRS score ≤ 1 before this attack);
- The informed consent approved by the ethics committee was voluntarily signed by the patient or his legal representative.

Exclusion Criteria

- Intracranial hemorrhagic diseases seen in head imaging: hemorrhagic stroke, epidural hematoma, intracranial hematoma, intraventricular hemorrhage, subarachnoid hemorrhage, etc; If it is only oozing blood, the researcher can judge whether it is suitable for inclusion
- Severe disturbance of consciousness: the item score of La consciousness level of NIHSS was more than 1;
- Transient ischemic attack (TIA)
- Systolic blood pressure was still higher than 220mmhg or diastolic blood pressure was higher than 120mmhg after blood pressure control;
- 5. Patients with severe mental disorders and dementia;
- 6. Severe active liver diseases have been diagnosed, such as acute hepatitis, chronic active hepatitis, cirrhosis, etc; Or ALT or AST > 2.0 × ULN;
- 7. Severe active kidney disease and renal insufficiency have been diagnosed; Or serum creatinine > 1.5 × ULN;
- 8. After the onset of the disease, the drugs with neuroprotective effect in the manual have been used
- 9. Embolectomy or interventional therapy has been used or planned after the onset of the disease;
- 10. Complicated with malignant tumor or undergoing anti-tumor treatment; For the subjects diagnosed with malignant tumor after enrollment, whether to continue to participate in the study can be judged by the researcher and the willingness of the subjects;
- 11. Suffering from severe systemic diseases, the estimated survival time is less than 90 days;
- 12. Allergic to d-borneol or edaravone or excipients;
- 13. Patients during pregnancy, lactation and planned pregnancy;
- 14. Major operation history within 4 weeks before enrollment;
- 15. Have participated in other clinical studies or are participating in other clinical studies within 30 days before randomization;
- 16. The researcher thinks that it is not suitable to participate in this clinical study

Abbreviations: ALT, alanine aminotransferase; AST, aspartate aminotransferase; NIHSS, National Institutes of Health Stroke Scale.

© 2024 Fu Y et al. *JAMA Neurology.*

eTable 2. Concomitant Medication Use Within 90 Days.

**Concomitant medication**	**Edaravone dexborneol group (N=450)**	**Placebo group (N=464)**	***P* value**
Hormonal agents (except sex hormones or insulin)	23 (5.1)	26 (5.6)	.74
Medications on sense organ	19 (4.2)	11 (2.4)	.12
Medications on respiratory system	89 (19.8)	97 (20.9)	.67
Medications on musculoskeletal system	64 (14.2)	67 (14.4)	.93
Medications on Antineoplastic and immunomodulatory disease	5 (1.1)	1 (0.2)	.12*
Medications on skin disease	27 (6.0)	17 (3.7)	.10
Medications on nervous system	260 (57.8)	252 (54.3)	.29
Medications on genitourinary system and sex hormones	30 (6.7)	31 (6.7)	.99
Medications on anti-infection	63 (14.0)	71 (15.3)	.58
Medications on digestive tract and metabolism	402 (89.3)	417 (89.9)	.80
Medications on cardiovascular disease	450 (100.0)	464 (100.0)	NA
Blood products and medications on hematopoietic organs	450 (100.0)	464 (100.0)	NA
Other	85 (18.9)	90 (19.4)	.85

NA denotes not applicant.

* Fisher exact test

© 2024 Fu Y et al. *JAMA Neurology.*

eTable 3. Sensitivity Analyses for the Primary Analysis

Sensitivity analysis	N	Edaravone dexborneol group	Placebo group	Risk difference (95% CI)	Odds ratio (95% CI)	*P* value
Best case analysis (assume any subject missing 90 day outcome to be mRS score ≤1)	914	323 (71.8)	283 (61.0)	10.79(4.70-16.87)	1.63(1.23-2.15)	<.001
Worst case analysis (assume any subject missing 90 day outcome to be mRS score >1)	914	283 (62.9)	251 (54.1)	8.79(2.43-15.16)	1.44(1.10-1.87)	.007
LOCF combined with best case analysis (assume any subject missing 90 day outcome after LOCF to be mRS score ≤1)	914	306 (68.0)	276 (59.5)	8.52(2.31-14.72)	1.45(1.10-1.90)	.008
LOCF combined with worst case analysis (assume any subject missing 90 day outcome after LOCF to be mRS score >1)	914	290 (64.4)	254 (54.7)	9.70(3.37-16.03)	1.50(1.15-1.95)	.003
Adjusted for clinical sites and time to randomization	914	290 (64.4)	254 (54.7)	9.70(3.37-16.03)	1.50(1.15-1.95)	.003
Complete case analysis	842	283 (69.0)	251(58.1)	10.92(4.47-17.38)	1.61(1.21-2.13)	0.001

Abbreviations: CI, confidence interval; LOCF, last observation carried forward; mRS, modified Rankin Scale

© 2024 Fu Y et al. *JAMA Neurology.*

eTable 4. Efficacy and Safety Outcomes in the Per-Population Analysis

Outcomes	Edaravone dexborneol group (N=390)	Placebo group (N=403)	Measurement of effect size	Effect size (95% CI)	*P* value
**Primary outcome**					
mRS score≤ 1 on day 90, n (%)	277 (71.0)	240 (59.6)	Risk difference	11.47(4.90 to 18.05)	
			Odds ratio	1.66(1.24 to 2.24)	<.001
**Secondary outcomes**					
mRS score on day 90, median (IQR)	1(0-2)	1(0-2)	Common odds ratio	1.41(1.10 to 1.82)	.009
mRS score≤ 2 on day 90, n (%)	322 (82.6)	316 (78.4)	Risk difference	4.15(-1.35 to 9.66)	
			Odds ratio	1.30(0.92 to 1.86)	.14
Changes of NIHSS score from baseline to day 14, mean (95% CI)*	-3(-4 to -3)	-3(-4 to -3)	Mean difference	0.03(-0.34 to 0.41)	.86
NIHSS score ≤ 1 on day 14, n (%)*	58 (14.9)	69 (17.2)	Risk difference	-2.25(-7.36 to 2.86)	
			Odds ratio	0.85(0.58 to 1.24)	.39
NIHSS score ≤ 1 on day 30, n (%)*	138 (36.2)	128 (32.6)	Risk difference	3.65(-3.04 to 10.34)	
			Odds ratio	1.18(0.87 to 1.58)	.29
NIHSS score ≤ 1 on day 90, n (%)*	224 (58.3)	207 (53.1)	Risk difference	5.26(-1.73 to 12.25)	
			Odds ratio	1.24(0.93 to 1.64)	.14
**Safety outcomes**					
AE within 90 days, n (%)	352 (90.3)	360 (89.3)	Risk difference	0.93(-3.29 to 5.14)	
			Odds ratio	1.11(0.70 to 1.75)	.67
TRAE within 90 days, n (%)	52 (13.3)	39 (9.7)	Risk difference	3.66(-0.78 to 8.10)	
			Odds ratio	1.44(0.92 to 2.23)	.11
SAE within 90 days, n (%)	25 (6.4)	31 (7.7)	Risk difference	-1.28(-4.84-2.28)	
			Odds ratio	0.82(0.48-1.42)	.48

© 2024 Fu Y et al. *JAMA Neurology.*

Abbreviations: AE, adverse event; IQR, interquartile range; mRS, modified Rankin Scale; NIHSS, National Institute of Health stroke scale; SAE, severe adverse events;TRAE, treatment related adverse events

*The number of patients with missing data on NIHSS score on day 14 was 1 in the edaravone dexborneol group, and 1 in the placebo group; missing data on NIHSS score on day 30 was 9 in the edaravone dexborneol group, and 10 in the placebo group; missing data on NIHSS score on day 90 was 6 in the edaravone dexborneol group, and 13 in the placebo group.

© 2024 Fu Y et al. *JAMA Neurology.*

eTable 5. Number of Patients with Adverse Events (by System Organ Class) During 90 Days of Follow-up*†

Adverse events	Edaravone dexborneol group (N=450)	Placebo group (N=464)	*P* value
Metabolism and nutrition disorders	208 (46.2)	223 (48.1)	.58
Ear and labyrinth disorders	2 (0.4)	2 (0.4)	1.00‡
Hepatobiliary disorders	82 (18.2)	80 (17.2)	.70
Infections and infestations	100 (22.2)	117 (25.2)	.29
Investigations	134 (29.8)	140 (30.2)	.90
Nervous system disorders	126 (28.0)	135 (29.1)	.71
Injury, poisoning and procedural complications	15 (3.3)	10 (2.2)	.28
Musculoskeletal and connective tissue disorders	33 (7.3)	45 (9.7)	.20
Congenital, familial and genetic disorders	2 (0.4)	4 (0.7)	.67‡
Respiratory, thoracic and mediastinal disorders	36 (8.0)	53 (11.4)	.08
Psychiatric disorders	86 (19.1)	82 (17.7)	.58
Neoplasms benign, malignant and unspecified (inclcysts and polyps)	3 (0.7)	1 (0.2)	.37‡
Immune system disorders	3 (0.7)	6 (1.3)	.51‡
Endocrine disorders	9 (2.0)	3 (0.7)	.07‡
Skin and subcutaneous tissue disorders	18 (4.0)	19 (4.1)	.94
General disorders and administration site conditions	41 (9.1)	36 (7.8)	.46
Renal and urinary disorders	53 (11.8)	55 (11.9)	.97
Reproductive system and breast disorders	9 (2.0)	11 (2.4)	.70
Gastrointestinal disorders	182 (40.4)	202 (43.5)	.34
Cardiac disorders	79 (17.6)	90 (19.4)	.47
Vascular disorders	35 (7.8)	52 (11.2)	.08
Blood and lymphatic system disorders	48 (10.7)	30 (6.5)	.02
Eye disorders	17 (3.8)	4 (0.9)	.003‡

*Adverse events did not include serious adverse events. Includes adverse events with an onset date on or after the date of first dose and up to the date of last dose of study medication.

† Patients with multiple events of one type were counted once.

‡ Fisher exact test.

© 2024 Fu Y et al. *JAMA Neurology.*

eTable 6. Number of Patients with Severe Adverse Events (by System Organ Class) During 90 Days of Follow-up*

**Severe adverse events**	**Edaravone dexborneol group (N=450)**	**Placebo group (N=464)**	***P* value**
Ear and labyrinth disorders	0 (0.0)	1 (0.2)	1.00†
Hepatobiliary disorders	2 (0.4)	0 (0.0)	.24†
Infections and infestations	4 (0.9)	1 (0.2)	.21†
Investigations	0 (0.0)	1 (0.2)	1.00†
Nervous system disorders	27 (6.0)	25 (5.4)	.71
Injury, poisoning and procedural complications	4 (0.9)	3 (0.7)	.72†
Musculoskeletal and connective tissue disorders	1 (0.2)	3 (0.7)	.62†
Congenital, familial and genetic disorders	1 (0.2)	2 (0.4)	1.00†
Respiratory, thoracic and mediastinal disorders	5 (1.1)	7 (1.5)	.79
Psychiatric disorders	1 (0.2)	0 (0.0)	.49†
Endocrine disorders	0 (0.0)	1 (0.2)	1.00†
General disorders and administration site conditions	3 (0.7)	3 (0.7)	1.00†
Renal and urinary disorders	0 (0.0)	1 (0.2)	1.00†
Gastrointestinal disorders	0 (0.0)	3 (0.7)	.25†
Cardiac disorders	2 (0.4)	5 (1.1)	.45†
Vascular disorders	1 (0.2)	0 (0.0)	.49†
Blood and lymphatic system disorders	1 (0.2)	0 (0.0)	.49†
Eye disorders	2 (0.4)	0 (0.0)	.24†

* Patients with multiple events of one type were counted once. Includes adverse events with an onset date on or after the date of first dose and up to the date of last dose of study medication.

† Fisher exact test.

© 2024 Fu Y et al. *JAMA Neurology.*

eTable 7. Number of Patients with Treatment Related Adverse Events During 90 Days of Follow-up*

Treatment related adverse events	Y-2 (N=450)	Placebo (N=464)
Hepatic function abnormal	18(4.0)	16(3.4)
Hypokalaemia	8(1.8)	2(0.4)
Constipation	7(1.6)	2(0.4)
Hypoproteinaemia	6(1.3)	4(0.9)
Hyperuricaemia	3(0.7)	0(0.0)
Anaemia	3(0.7)	2(0.4)
Hypertriglyceridaemia	2(0.4)	0(0.0)
Hyperhomocysteinaemia	2(0.4)	3(0.6)
Alanine aminotransferase increased	2(0.4)	2(0.4)
Aspartate aminotransferase increased	2(0.4)	2(0.4)
Blood fibrinogen increased	2(0.4)	0(0.0)
Transaminases increased	2(0.4)	2(0.4)
Abdominal discomfort	2(0.4)	0(0.0)
Urinary tract infection	2(0.4)	1(0.2)
Hepatic failure	1(0.2)	0(0.0)
Drug-induced liver injury	1(0.2)	0(0.0)
Gamma-glutamyltransferase increased	1(0.2)	1(0.2)
Protein urine present	1(0.2)	0(0.0)
Urinary occult blood positive	1(0.2)	1(0.2)
Renal function test abnormal	1(0.2)	0(0.0)
Blood creatinine increased	1(0.2)	1(0.2)
Blood uric acid increased	1(0.2)	0(0.0)
Blood glucose increased	1(0.2)	0(0.0)
Platelet count increased	1(0.2)	0(0.0)
Lipoprotein(a) increased	1(0.2)	1(0.2)
Lipoprotein increased	1(0.2)	0(0.0)
Gastrointestinal haemorrhage	1(0.2)	0(0.0)
Dyspepsia	1(0.2)	0(0.0)
Restless legs syndrome	1(0.2)	0(0.0)
Cerebral infarction	1(0.2)	1(0.2)
Cerebrovascular accident	1(0.2)	0(0.0)
Head discomfort	1(0.2)	0(0.0)
Headache	1(0.2)	0(0.0)
Infection	1(0.2)	1(0.2)
Gastroenteritis	1(0.2)	0(0.0)
Proteinuria	1(0.2)	0(0.0)
Haemorrhage urinary tract	1(0.2)	0(0.0)
Renal impairment	1(0.2)	3(0.6)
Haematuria	1(0.2)	0(0.0)
Rhabdomyolysis	1(0.2)	0(0.0)

© 2024 Fu Y et al. *JAMA Neurology.*

Treatment related adverse events	Y-2 (N=450)	Placebo (N=464)
Pain in extremity	1(0.2)	0(0.0)
Myocardial damage	1(0.2)	0(0.0)
Arrhythmia	1(0.2)	0(0.0)
Insomnia	1(0.2)	1(0.2)
Hypersensitivity	1(0.2)	0(0.0)
Hyperhidrosis	1(0.2)	0(0.0)
Ocular discomfort	1(0.2)	0(0.0)
Hyperbilirubinaemia	0(0.0)	2(0.4)
Hypoalbuminaemia	0(0.0)	1(0.2)
Hyperlipidaemia	0(0.0)	1(0.2)
White blood cell count decreased	0(0.0)	1(0.2)
White blood cell count increased	0(0.0)	1(0.2)
Myocardial necrosis marker increased	0(0.0)	2(0.4)
Creatine phosphokinase MB increased	0(0.0)	2(0.4)
Platelet count decreased	0(0.0)	1(0.2)
Diarrhoea	0(0.0)	1(0.2)
Vomiting	0(0.0)	1(0.2)
Upper gastrointestinal haemorrhage	0(0.0)	1(0.2)
Haemorrhagic transformation stroke	0(0.0)	1(0.2)
Genitourinary tract infection	0(0.0)	1(0.2)
Arteriosclerosis coronary artery	0(0.0)	1(0.2)
Ventricular extrasystoles	0(0.0)	1(0.2)
Sinus bradycardia	0(0.0)	1(0.2)
Rash	0(0.0)	1(0.2)
Urticaria	0(0.0)	1(0.2)
Productive cough	0(0.0)	1(0.2)

* Patients with multiple events of one type were counted once. Includes adverse events with an onset date on or after the date of first dose and up to the date of last dose of study medication.

© 2024 Fu Y et al. *JAMA Neurology.*

eTable 8. Number of Patients with Clinically Significant Changes in Vital Signs and Laboratory Data on Day 90 of Follow-up*

Variables	Edaravone dexborneol group (N=450)	Placebo group (N=464)	*P* value
**Vital Signs**			
Temperature	3 (0.7)	5 (1.1)	.50†
Systolic blood pressure	80 (17.8)	77 (16.6)	.64
Diastolic blood pressure	72 (16.0)	86 (18.5)	.31
Heart rate	24 (5.3)	29 (6.3)	.55
Breath	0 (0.0)	0 (0.0)	NA
**Laboratory Data**			
White blood cell	16 (3.6)	18 (3.9)	.8
Neutrophil count	16 (3.6)	15 (3.2)	.79
Lymphocyte count	3 (0.7)	2 (0.4)	.63†
Hemoglobin	25 (5.6)	16 (3.4)	.12
Platelet	10 (2.2)	8 (1.7)	.59
Urinary white blood cells	37 (8.2)	47 (10.1)	.32
Urine protein	29 (6.5)	35 (7.5)	.52
Urine glucose	25 (5.6)	26 (5.6)	.98
Urine ketone body	13 (2.9)	9 (1.9)	.35
Urine occult blood	40 (9.9)	40 (9.6)	.88
International normalized ratio	3 (0.7)	5 (1.1)	.50†
Prothrombin time	3 (0.7)	4 (0.9)	.73†
Activated partial thromboplastin time	5 (1.1)	3 (0.6)	.45†
Fibrinogen	22 (4.9)	16 (3.4)	.28
Alanine aminotransferase	75 (16.7)	72 (15.5)	.64
Glutamic transaminase	53 (11.8)	65 (14.0)	.31
Alkaline phosphatase	10 (2.2)	4 (0.9)	.09†
Total bilirubin	5 (1.1)	9 (1.9)	.31
Direct bilirubin	9 (2.0)	13 (2.8)	.43
Creatinine	11 (2.4)	17 (3.7)	.29
Urea	12 (3.4)	7 (1.9)	.21
Urea nitrogen	0 (0.0)	2 (2.1)	.16†
Total protein	20 (4.4)	27 (5.8)	.35
Albumin	26 (5.8)	30 (6.5)	.66
Glucose	54 (12.1)	53 (11.4)	.77
Total cholesterol	18 (4.0)	13 (2.8)	.32
Triglyceride	48 (10.7)	57 (12.3)	.44
Creatine kinase	9 (2.0)	6 (1.3)	.40
Creatine kinase isoenzyme	10 (2.2)	15 (3.2)	.35
Lactate dehydrogenase	10 (2.2)	6 (1.3)	.29
Serum potassium	47 (10.5)	57 (12.3)	.39
Serum sodium	9 (2.0)	19 (4.1)	.07

© 2024 Fu Y et al. *JAMA Neurology.*

Serum chlorine	5 (1.1)	8 (1.7)	.44
Homocysteine	56 (12.5)	53 (11.4)	.62

NA denotes not applicable

*Clinically significant changes indicated that the parameter reached a clinically significance abnormal level at the visit on day 90.

† Fisher exact test.

© 2024 Fu Y et al. *JAMA Neurology.*

eTable 9. Laboratory data on day 90 after randomization

Variables	Edaravone dexborneol group (N=450)	Placebo group (N=464)	*P* value
**Vital Signs**			
Temperature	36.4(36.3-36.5)	36.4(36.3-36.5)	0.38
Systolic blood pressure	133(128-142)	135(130-145)	0.03
Diastolic blood pressure	81(77-87)	82.5(79-89)	0.02
Heart rate	72(63-80)	72(64-79)	0.99
Breath	19(18-20)	19(18-20)	0.70
**Laboratory Data**			
White blood cell	6.7(5.7-8)	6.5(5.6-7.8)	0.18
Neutrophil count	4.2(3.3-5.2)	4(3.3-4.9)	0.10
Lymphocyte count	1.8(1.5-2.3)	1.9(1.4-2.3)	0.88
Hemoglobin	141(131-150)	142(132-151)	0.40
Platelet	241(203-274)	230(190-266)	0.01
International normalized ratio	1(0.9-1)	1(0.9-1)	0.51
Prothrombin time	11.6(10.9-12.4)	11.7(11-12.4)	0.41
Activated partial thromboplastin time	29.8(26.5-33.6)	29.6(26.6-34.1)	0.98
Fibrinogen	3.2(2.8-3.7)	3.2(2.7-3.7)	0.59
Alanine aminotransferase	22(16-35.7)	22.7(16-33.6)	0.99
Glutamic transaminase	21(17-27)	21(17-27)	0.98
Alkaline phosphatase	85.5(71-103)	85(71.4-101)	0.76
Total bilirubin	12.5(9.2-15.4)	12.5(9.8-16.8)	0.23
Direct bilirubin	3.8(2.8-5.1)	3.9(2.8-5.3)	0.35
Creatinine	68(56.6-80)	69.8(58-80.2)	0.6
Urea	5.3(4.3-6.3)	5(4.4-6.1)	0.36
Urea nitrogen	5.2(4.4-6.2)	5.3(4.7-6.8)	0.27
Total protein	72.9(69.6-75.3)	72.5(69.2-75.4)	0.27
Albumin	45.2(42.9-47.1)	44.7(42.6-46.7)	0.21
Glucose	5.8(5.2-7.2)	5.7(5.2-6.9)	0.48
Total cholesterol	3.8(3.2-4.5)	3.7(3.2-4.4)	0.24
Triglyceride	1.3(0.9-1.8)	1.4(1-1.8)	0.28
Creatine kinase	80(58-111)	80(59-113)	0.83
Creatine kinase isoenzyme	13.3(11-17)	13.4(11-17)	0.84
Lactate dehydrogenase	174(152-199)	178(155-201)	0.28
Serum potassium	4.2(4-4.4)	4.2(4-4.5)	0.96
Serum sodium	141(139-142.2)	141(139.2-142.6)	0.41
Serum chlorine	103.6(101.8-105.3)	103.8(101.9-105.7)	0.55
Homocysteine	11.9(10.2-14.7)	12.2(10.2-14.9)	0.56

© 2024 Fu Y et al. *JAMA Neurology.*

eTable 10. Laboratory data on day 30 after randomization

Variables	Edaravone dexborneol group (N=450)	Placebo group (N=464)	*P* value
**Vital Signs**			
Temperature	36.4(36.2-36.5)	36.4(36.2-36.5)	0.74
Systolic blood pressure	135(129.5-145)	136(129-145)	0.99
Diastolic blood pressure	82(78-88)	82.5(78-89)	0.66
Heart rate	72(64-80)	72(63-80)	0.83
Breath	19(18-20)	19(18-20)	0.91
**Laboratory Data**			
White blood cell	6.9(6-8.2)	6.8(5.7-7.8)	0.07
Neutrophil count	4.4(3.7-5.5)	4.2(3.4-5.1)	0.06
Lymphocyte count	1.8(1.5-2.2)	1.8(1.4-2.2)	0.93
Hemoglobin	145(132-154)	143(133-153)	0.76
Platelet	236(197-274)	231(193.5-275)	0.23
International normalized ratio	1(0.9-1)	1(0.9-1)	0.78
Prothrombin time	11.8(10.9-12.5)	11.7(10.9-12.5)	0.87
Activated partial thromboplastin time	29.7(26.7-34.5)	29.9(26.4-33.7)	0.36
Fibrinogen	3.3(2.9-3.9)	3.4(3-3.9)	0.39
Alanine aminotransferase	25.9(17-41)	25.1(17.6-37)	0.88
Glutamic transaminase	23(18.2-28.8)	23(18.2-29.2)	0.61
Alkaline phosphatase	87(74-108)	90(73-107)	0.98
Total bilirubin	12.1(9-15.2)	12.3(9.5-16.3)	0.09
Direct bilirubin	3.7(2.7-5.2)	3.8(2.9-5.2)	0.19
Creatinine	68(58-80)	69(57.1-80.3)	0.77
Urea	5.1(4.2-6.3)	5.1(4.2-6.2)	0.72
Urea nitrogen	5.2(4.3-6.5)	5.7(4.7-6.6)	0.11
Total protein	72.5(69.4-75.7)	72.7(69.4-75.3)	0.80
Albumin	44.7(42.1-46.8)	44.4(42.4-46.9)	0.72
Glucose	5.9(5.2-7.8)	5.8(5.1-7.1)	0.05
Total cholesterol	3.7(3.1-4.3)	3.5(3.1-4.2)	0.04
Triglyceride	1.3(1-1.9)	1.3(1-1.8)	0.50
Creatine kinase	74(52-107)	72(51-101)	0.62
Creatine kinase isoenzyme	13.9(11-17.4)	14(11-17.8)	0.86
Lactate dehydrogenase	179(156-204)	181(157.3-205.5)	0.61
Serum potassium	4.3(4-4.5)	4.2(4-4.5)	0.06
Serum sodium	140.6(139-142.3)	140.8(139-142.3)	0.59
Serum chlorine	103.4(101.2-105.1)	103(101.3-105.1)	0.96
Homocysteine	12.2(10-14.5)	11.9(9.7-14.4)	0.24

© 2024 Fu Y et al. *JAMA Neurology.*

eTable 11. Laboratory data on day 14 after randomization

Variables	Edaravone dexborneol group (N=450)	Placebo group (N=464)	*P* value
**Vital Signs**			
Temperature	36.4(36.2-36.5)	36.4(36.2-36.5)	.21
Systolic blood pressure	135(128-147)	137(130-146)	.43
Diastolic blood pressure	81(76-88)	82(77-89)	.56
Heart rate	72(64-80)	72(64-80)	.88
Breath	19(18-20)	19(18-20)	.32
**Laboratory Data**			
White blood cell	7.1(6.1-8.5)	7.1(6-8.2)	.27
Neutrophil count	4.6(3.7-5.7)	4.5(3.6-5.6)	.19
Lymphocyte count	1.8(1.4-2.2)	1.8(1.4-2.2)	.48
Hemoglobin	141.5(130-152)	141.5(130-151)	.82
Platelet	250(212.5-292.5)	242(205-287)	.05
International normalized ratio	1(0.9-1)	1(0.9-1)	.38
Prothrombin time	11.8(11-12.6)	11.9(11-12.7)	.69
Activated partial thromboplastin time	30(26.8-34)	30(26.7-33.7)	.41
Fibrinogen	3.5(2.9-4.2)	3.4(2.9-4.1)	.24
Alanine aminotransferase	23(16.1-35)	24(17-33.6)	.49
Glutamic transaminase	22(17.7-28)	23.3(18-29)	.21
Alkaline phosphatase	87(71-104)	84(69-101)	.56
Total bilirubin	11.6(9.2-15.2)	12(9.3-15.4)	.40
Direct bilirubin	3.8(2.7-5.1)	3.7(2.7-5.2)	.66
Creatinine	67(57-77.8)	68.5(57-80.6)	.42
Urea	5.1(4-6.3)	5.1(4.2-6.2)	.91
Urea nitrogen	5.3(4.4-6.5)	5.6(4.5-6.9)	.23
Total protein	69.3(65-73.8)	68.5(64.4-73.2)	.09
Albumin	42.3(39.4-45.5)	42(39-44.8)	.08
Glucose	5.6(4.8-7.1)	5.5(4.8-6.7)	.33
Total cholesterol	3.4(2.9-4)	3.4(2.8-3.9)	.18
Triglyceride	1.2(0.9-1.6)	1.2(0.9-1.7)	.89
Creatine kinase	69(49-104)	71(52-105)	.73
Creatine kinase isoenzyme	15(11.5-18.3)	14.4(11-19.4)	.74
Lactate dehydrogenase	181(160-210)	184(161-211)	.39
Serum potassium	4.1(3.8-4.4)	4.1(3.8-4.3)	.45
Serum sodium	141(139-142.7)	141(139.4-142.6)	.92
Serum chlorine	103.6(101.4-105.1)	103.7(101.6-105.3)	.65
Homocysteine	12.3(10.2-15.4)	12.5(10.2-15.2)	.80

© 2024 Fu Y et al. *JAMA Neurology.*

eTable 12. Laboratory data on day 7 after randomization

Variables	Edaravone dexborneol group (N=450)	Placebo group (N=464)	*P* value
**Vital Signs**			
Temperature	36.4(36.3-36.5)	36.4(36.3-36.5)	.98
Systolic blood pressure	139(130-151)	140(130-154)	.16
Diastolic blood pressure	82(75-90)	83(76-90)	.15
Heart rate	69(62-77)	70(62-78)	.24
Breath	19(18-20)	19(18-20)	.71
**Laboratory Data**			
White blood cell	6.9(5.9-8.5)	6.8(5.8-8.1)	.18
Neutrophil count	4.4(3.6-5.6)	4.3(3.4-5.4)	.11
Lymphocyte count	1.7(1.4-2.2)	1.7(1.3-2.1)	.46
Hemoglobin	138(127-148)	138(129-149)	.52
Platelet	226(191-265)	219(181-258)	.10
International normalized ratio	1(0.9-1)	1(0.9-1)	.38
Prothrombin time	11.7(10.8-12.7)	11.8(10.9-12.7)	.44
Activated partial thromboplastin time	29.6(26.8-34.2)	29.2(26.2-33.3)	.11
Fibrinogen	3.2(2.7-3.9)	3.2(2.8-3.8)	.84
Alanine aminotransferase	18.3(13-28)	19.4(14-27.9)	.23
Glutamic transaminase	20(16.4-25.7)	21(17-26.8)	.19
Alkaline phosphatase	81(66-93)	80.2(67-95)	.53
Total bilirubin	11.9(8.8-15.5)	12.1(8.9-16.1)	.56
Direct bilirubin	3.7(2.7-5.1)	3.7(2.6-5.4)	.85
Creatinine	66(54-77)	67(56-78)	.37
Urea	4.9(4-5.9)	4.7(3.9-6)	.75
Urea nitrogen	5.1(4.1-6.2)	4.9(4-6.2)	.82
Total protein	65.4(61-69.1)	65.3(61.4-68.8)	.82
Albumin	39.9(37.5-42.2)	39.5(37.2-41.9)	.35
Glucose	5.3(4.6-7)	5.3(4.7-6.6)	.92
Total cholesterol	3.6(3.1-4.2)	3.5(3-4.1)	.33
Triglyceride	1.3(0.9-1.7)	1.2(1-1.7)	.63
Creatine kinase	64(48-92)	69(50-92)	.16
Creatine kinase isoenzyme	15(11.8-19.9)	14.8(11.1-20.1)	.94
Lactate dehydrogenase	174.5(154-203.5)	177.1(159-209)	.12
Serum potassium	3.9(3.7-4.2)	3.9(3.6-4.2)	.20
Serum sodium	141.1(139.8-143)	141.3(139.3-142.9)	.83
Serum chlorine	104.5(102.2-106)	104.1(102-106)	.34
Homocysteine	13(10.5-17.1)	13(10.7-16.9)	.93

© 2024 Fu Y et al. *JAMA Neurology.*
