# Supplementary material for: Sublingual Edaravone Dexborneol for the Treatment of Acute Ischemic Stroke: The TASTE-SL Randomized Clinical Trial
Source: JAMA Neurol. 2024 Feb 19;81(4):319–26. doi: 10.1001/jamaneurol.2023.5716 (PMC10877503; doi:10.1001/jamaneurol.2023.5716)
Supplement: Supplement 3. — Data Sharing Statement. [file jamaneurol-e235716-s003.pdf]

Data Sharing Statement

Fu. Sublingual Edaravone Dexborneol for the Treatment of Acute Ischemic Stroke. *JAMA Neurol*. Published February 19, 2024. doi:10.1001/jamaneurol.2023.5716

Data
Data available: No
